# Transcribed-ultra conserved region expression profiling from low-input total RNA

**DOI:** 10.1186/1471-2164-11-149

**Published:** 2010-03-03

**Authors:** Paola Scaruffi, Sara Stigliani, Simona Coco, Franscesca Valdora, Carla De Vecchi, Stefano Bonassi, Gian Paolo Tonini

**Affiliations:** 1 Translational Paediatric Oncology, National Cancer Research Institute (IST), Largo R Benzi 10, Genoa, 16132, Italy; 2 Department of Oncology and Genetics (DOBIG), University of Genoa, Largo R Benzi 10, Genoa, 16132, Italy; 3 Clinical and Molecular Epidemiology, IRCCS San Raffaele Pisana, Via della Pisana 235, Roma, 00163, Italy

## Abstract

**Background:**

Ultra Conserved Regions (UCRs) are a class of 481 noncoding sequences located in both intra- and inter-genic regions of the genome. The recent findings that they are significantly altered in adult chronic lymphocytic leukemias, carcinomas, and pediatric neuroblastomas lead to the hypothesis that UCRs may play a role in tumorigenesis.

**Results:**

We present a novel application of Ribo-SPIA™ isothermal linear amplification of minute RNA quantities for quantifying Transcribed-UCR (T-UCR) expression by quantitative PCR. Direct comparison of non-amplified with amplified cDNA in two neuroblastoma cell lines showed that the amplification approach increases sensitivity and repeatability in T-UCR quantification. It is noteworthy that the Ribo-SPIA™ step allowed us to analyze all 481 T-UCRs by using 150 ng of RNA, while introducing a minimal bias and preserving the magnitude of relative expression. Only the less abundant T-UCRs have high intra-assay variability, consistently with the Poisson distribution statistics and stochastic effects on PCR repeatability.

**Conclusions:**

We demonstrated that the quantification procedure shown here is an accurate and reliable technique for genome-wide non coding gene (i.e., UCRs) profiling using small amounts of RNA. This issue is particularly important because studies of transcription regulation are increasingly conducted in small homogeneous samples, such as laser capture microdissected or sorted cell populations.

## Background

Ultra Conserved Regions (UCRs) are a class of noncoding sequences discovered in 2004 by Bejerano and colleagues [1,2]. The UCRs are located in both intra- and inter-genic regions and 53% of them are nonexonic, whereas the remaining 47% are either overlapping mRNAs or possibly exonic. Since UCRs represent a group of 481 sequences (200-779 bp in length) that are 100% conserved among orthologous regions of human, mouse, and rat genomes, it has been suggested they may be involved in ontogeny and phylogeny of mammals and other vertebrates [3]. Previous studies identified a functional role for these noncoding sequences both in regulation of alternative splicing and in promoting the expression of several genes [3]. Calin et al. [4] demonstrated that UCRs are significantly altered at both genomic and expression levels in adult chronic lymphocytic leukemias, colorectal and hepatocellular carcinomas. We recently profiled both T-UCRs and microRNAs in a cohort of 34 high-risk neuroblastoma (NB) tumors, with the aim to investigate their putative role as sensitive markers of outcome prediction in children with stage 4 NB [5]. Our study identified for the first time a signature based on T-UCR expression that is associated with good outcome in non infant patients diagnosed with metastatic NB. Moreover, our findings strongly suggest that a deregulation of the microRNA/T-UCR network may play an important role in the pathogenesis of neuroblastoma.

Microarrays, Northern blot and reverse transcription-quantitative real-time PCR (RT-qPCR) have been successfully used to detect T-UCR expression [4]. However, these methods preclude the use of many samples of potential clinical interest, such as tumor biopsies, laser microdissected tissues, and sorted cell populations, because of the high quantities of RNA required for expression analysis of all 481 T-UCRs. On this account, accurate high-throughput profiling of T-UCRs starting from low-input RNA is an emerging challenge in molecular biology.

Ribo-SPIA™ technology [6], developed by NuGEN™ Technologies, enables a fast, simple and sensitive method for preparing amplified cDNA from total RNA to detect low, medium, and high abundance transcripts. The amplified product is single-stranded cDNA in the antisense direction of the starting RNA and it is ready for qPCR without any purification. Such RNA amplification procedure is suitable for expression analysis of both eukaryotic and prokaryotic genes [7,8]. For the first time here we present a novel application of the Ribo-SPIA™ isothermal linear amplification of minute RNA quantities for quantifying non-coding protein gene expression.

## Methods

### Cell lines and RNA isolation

Two NB cell lines (LAN-5 and GI-ME-N) (provided by Interlab Cell Line Collection (ICLC), http://www.iclc.it) were cultured at 37°C and 5% CO_2 _in RPMI 1640 medium (Lonza, Basel, Switzerland), supplemented with 2 mM L-glutamine, 1% non-essential amino acids, and 10% foetal bovine serum. Total RNA was extracted by using the PerfectPure™ RNA Cell Kit (5Prime, Hamburg, Germany), including on-column RNase-free DNase I treatment (Ambion, TX, USA). Total and small RNAs were quantified and quality control was assessed by RNA 6000 Nano^® ^and Small RNA^® ^assays, respectively, on the 2100 Bioanalyzer (Agilent Technologies, Santa Clara, CA). RNA samples showed a RNA integrity number = 9.2. Both RNA samples have been validated as DNA-free by a no-reverse transcription assay for Eukaryotic 18S rRNA gene.

### Reverse transcription of total RNA

RNA (1 μg) was reverse transcribed using 20 pmoles of random hexamers (Eppendorf, Hamburg, Germany) and 200 U of SuperScript II enzyme (Invitrogen Life Technologies, Carlsbad, CA) in a total reaction volume of 20 μl. The mixture was incubated in a Mastercycler^® ^epGradient S (Eppendorf) at 25°C for 10 minutes, 42°C for 60 minutes, and 85°C for 5 minutes to stop the reaction. cDNA was diluted 10-fold in molecular biology grade water to a final concentration of 5 ng/ul, assuming 100% reverse transcription efficiency.

### Reverse transcription and amplification of total RNA

RNA was reverse transcribed and amplified by the WT-Ovation™ RNA Amplification System kit (NuGEN Technologies, San Carlos, CA) following manufacture's protocol and using the Mastercycler^® ^epGradient S (Eppendorf). In each reaction RNA input was 50 ng and cDNA product was diluted 1:25 in molecular biology grade water.

### Quantification of T-UCRs by qPCR

T-UCRs were quantified by pre-optimized Transcribed Ultra Conserved Regions real-time PCR assays (PrimerDesign Ltd, Hants, UK) using SYBR^® ^green chemistry. Each assay was individually validated by PrimerDesign Ltd and shown to be 100% specific and close to 100% efficient. The qPCR reactions were carried out in a total volume of 10 μl, containing 2 μl of diluted cDNA, 2.5× RealMaster Mix SYBR ROX (5Prime) and 150 nM of the specific T-UCR primer mix. Quantification of 18S rRNA, used as reference gene, was performed by using VIC-labeled TaqMan^® ^Gene Expression assay (Applied Biosystems, Foster City, CA) in a total volume of 10 μl, containing 2 μl of diluted cDNA, 2.5× RealMaster Mix Probe (5Prime) and 20× primer/probe mix. Reactions were setup in 96-white-well Twin.tec^® ^real-time plates (Eppendorf) by means of EpMotion 5070 Liquid Handling Workstation (Eppendorf). All reactions were performed in duplicate on the Mastercycler^® ^epRealPlex^4 ^S system (Eppendorf). Cycling conditions were as follows: 95°C for 2 minutes, 40 cycles at 95°C for 15 seconds and at 60°C for 1 minutes, followed by a melting curve (ramping from 60°C to 95°C in 20 minutes) to ensure the presence of the specific amplicon. Specificity of qPCR reactions has been assessed by melting curve analysis and single runs were excluded when the melting curves revealed unintended amplification products (e.g., primer dimers): curves with more than one peak, or one single peak but with a melting temperature different from the expected one (calculated by PrimerDesign's design software, accounting salt conditions of the mastermix). Raw qPCR data, melting temperatures and a checklist according to the Minimum Information for Publication of Quantitative Real-Time PCR Experiments (MIQE) [9,10] are submitted as Supplemental data (see additional file 1: Raw Cq data; additional file 2: Tm data; additional file 3: MIQE checklist).

### Data analysis

RealPlex software v. 2.0 (Eppendorf) was used to determine quantification cycle (Cq) in non-amplified (Cq_NA_) and amplified (Cq_A_) total RNA samples. For each cDNA, the duplicate T-UCR Cq values were averaged, and the normalized Cq (dCq) was calculated by subtracting the mean Cq value for 18S rRNA from each T-UCR mean Cq value. Potential bias introduced upon the cDNA amplification step and reliability of differential T-UCR expression were assessed as previously described by Mestdagh et al. [11]. Briefly, differential T-UCR expression between LAN-5 and GI-ME-N cell lines (ΔCq = dCq_GI-ME-N _- dCq_LAN-5_) was determined for each method, with (ΔCq_A_) or without (ΔCq_NA_) amplification. Then, we have calculated the difference in differential T-UCR expression (ΔΔCq = ΔCq_A _- ΔCq_NA_). Optimal reliability of differential T-UCR expression upon cDNA amplification should result in a difference lower than 1 PCR cycle (|ΔΔCq| < 1). The MedCalc^® ^software (Mariakerke, Belgium) was used for statistical analyses. Significance tests were two tailed.

## Results

### Amplification approach increases sensitivity in T-UCR quantification

In this study we analyzed expression of 481 UCRs in LAN-5 and GI-ME-N cell lines by RT-qPCR either with or without cDNA amplification. Since it is known that a single copy target is generally detected around cycle 35, those T-UCRs showing a Cq_NA _above 35 were considered as not expressed. In qPCR assays performed on non-amplified GI-ME-N and LAN-5 cDNAs the quantification rates were 384 and 357 T-UCRs, respectively, whereas upon amplification the quantification rate increased on average of 12% (up to 438 and 423 T-UCRs in GI-ME-N and LAN-5, respectively). The results above demonstrate the improvement of informative data interrogation by RT-qPCR of the amplified samples as compared to those generated using reverse transcription of total RNA. It is noteworthy that the qPCR reaction inputs were equivalent between amplified and non-amplified samples. In fact, assuming 100% amplification efficiency according to the WT-Ovation™ technical reports http://www.nugeninc.com, amplification of 50 ng total RNA should result in about 4 μg of cDNA. The cDNA in 40 μl was diluted 1:25, then 2 μl (approximately cDNA equivalents of 8 ng) were added per qPCR run. Similarly, cDNA equivalents of 10 ng were used for non-amplified cDNA.

### Amplification approach increases repeatability in T-UCR quantification

**Figure 1:**
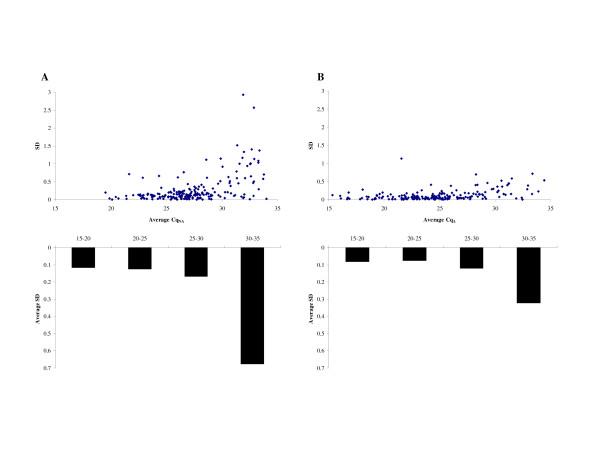
**Plots showing the relation between the average Cq value and the standard deviation for T-UCRs in LAN-5 cell line, using reverse transcriptase of total RNA (A) and amplification system (B)**. Bar plots display the mean SD value for T-UCRs with an average Cq value ranging between 15-20, >20-25, >25-30, and >30-35 cycles.

We validated qPCR results by measuring intra-assay variation. We performed duplicate qPCR runs and rough Cq values were used to calculate the standard deviation (SD) for the Cq variance of each T-UCR. Highly and moderately expressed T-UCRs (Cq_NA _≤ 30) have low SD in both non-amplified (average SD = 0.16) and amplified (average SD = 0.09) LAN-5 templates (Figure 1). The SD values increase for low expressed T-UCRs (30 < Cq_NA _≤ 35), for both amplified and non-amplified cDNA, showing a relevant PCR variability only at the lowest abundant ranges. Similar results were obtained in GI-ME-N cell line (see additional file 4: Figure S1). In LAN-5 cells, the number of assessable duplicated T-UCR runs with a SD ≤ 1 significantly increases upon the amplification process (378 out of 398 in non-amplified cDNA *versus *372 out of 373 in amplified cDNA, p < 0.0001, Fisher's exact test). Similarly, 348 out of 359 T-UCRs show a SD ≤ 1 in non-amplified GI-ME-N cDNA compared to 358 out of 359 T-UCRs in amplified cDNA (p = 0.006, Fisher's exact test).

### Minimal bias due to the amplification approach

**Figure 2:**
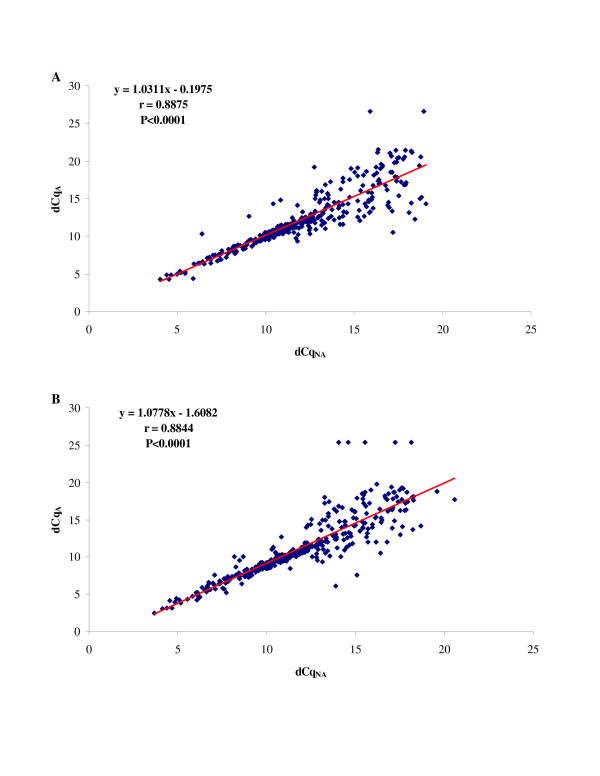
**Scatter plots showing the linear correlation (*r*) between normalized Cq values obtained with the SPIA™ system (dCq_A_) and by reverse transcriptase of total RNA (dCq_NA_) in LAN-5 (A) and GI-ME-N (B) cell lines**.

In order to assess the potential bias introduced upon the amplification step in each NB cell line, normalized Cq values obtained with the WT-Ovation™ system (dCq_A_) were compared with those obtained by reverse transcriptase of total RNA (dCq_NA_). Scatter plots for both LAN-5 and GI-ME-N samples (Figure 2) show a linear correlation between normalized Cq with or without amplification with correlation coefficient r ≅ 0.88, indicating that the amplification approach provides a reliable representation of T-UCR transcript abundance in non-amplified cDNA. In order to evaluate why data sets do not perfectly fit with a straight line correlation model, we divided T-UCRs into three groups: highly (Cq_NA _≤ 25), moderately (25 < Cq_NA _≤ 30), and low expressed (30 < Cq_NA _≤ 35) T-UCRs. Upon amplification step in LAN-5 cell line, linear correlation analysis shows high biases for the low abundant T-UCRs (r = 0.2696) as compared to the moderately (r = 0.7494) and highly (r = 0.8880) expressed T-UCRs. Similar results were obtained in GI-ME-N cell line: r = 0.4508, 0.7259, and 0.9056 for low, moderately and highly expressed T-UCRs, respectively.

### Amplification method preserves differential T-UCR expression

**Figure 3:**
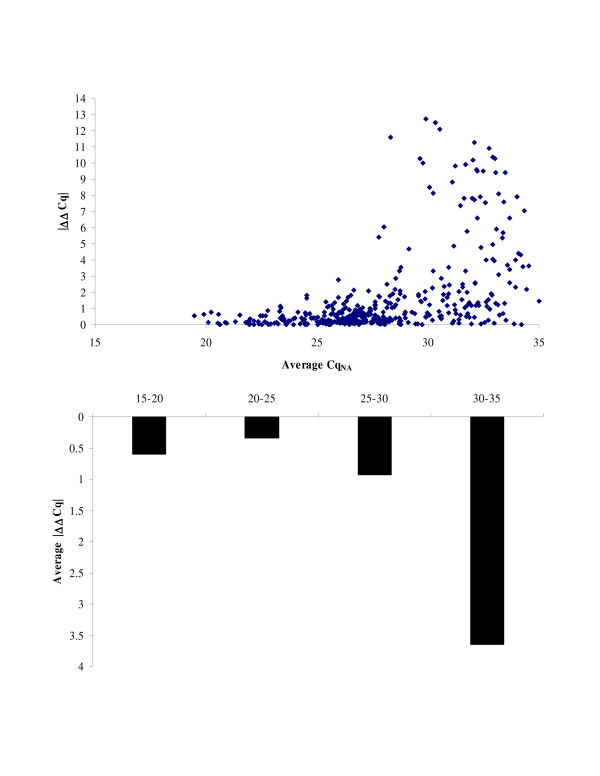
**Plot of the difference in differential T-UCR expression between LAN-5 and GI-ME-N cell lines against each mean dCq_NA _value**. Bar plot shows the mean |ΔΔCq| value for T-UCRs with an average Cq_NA _value ranging between 15-20, >20-25, >25-30, and >30-35 cycles.

We wondered whether upon amplification step the level of T-UCR expression was maintained. For this purpose, we calculated the difference between LAN-5 and GI-ME-N in T-UCR expression measured both with and without amplification. The plot of the differential T-UCR expression against each dCq_NA _value shows that 65% of the detectable T-UCRs display a |ΔΔCq| ≤ 1 (Figure 3). Moreover, our results show a near-perfect correlation in differential T-UCR expression (|ΔΔCq| = 0.4) for the most abundant T-UCRs, whereas the average |ΔΔCq| value increases for the moderately expressed T-UCRs (average |ΔΔCq| = 0.9), and further for the low expressed T-UCRs (average |ΔΔCq| = 3.6). Consistently, by setting the Cq_NA _cutoff at 30, 81% of the detectable T-UCRs display a |ΔΔCq| ≤ 1.

## Discussion

In this study, we evaluated the sensitivity, repeatability, and reliability of a cDNA amplification procedure for estimating T-UCR expression by RT-qPCR. This approach comprises steps for cDNA synthesis followed by SPIA™-based linear, isothermal amplification initiated both at the 3' end and randomly throughout the whole transcriptome [6]. Quantification of the corresponding single-stranded cDNA product is based on the SYBR^® ^Green intercalating chemistry. We directly compared non-amplified with amplified cDNAs of LAN-5 and GI-ME-N neuroblastoma cell lines. It is noteworthy that we accurately investigated the advantages of the amplification approach by using equivalent cDNA inputs (10 ng) for each amplified and non-amplified sample. The superior sensitivity of a total RNA amplification prior to qPCR is demonstrated, in fact about 12% more T-UCRs were reliably and reproducibly quantified by the SPIA™ system than using reverse transcriptase of total RNA, independently of the cell line.

Three separate RNA reverse transcriptions and three amplification reactions were performed per sample to interrogate all 481 T-UCRs. Therefore, to minimize the bias in RNA processing efficiency, we separately pooled non-amplified and amplified cDNAs necessary for all subsequent qPCR runs. However, we measured intra-assay variation due to i.e. robotic liquid handling and PCR equipments by calculating the amount of error when T-UCR assays were performed in duplicate. Standard deviation values showed an high repeatability of sample replicates for highly and moderately expressed T-UCRs, independently of the quantification method, indicating the unbiased amplification procedure. Only the lowest abundant T-UCRs have high intra-assay variability, consistently with the influence of Poisson distribution statistics and stochastic effects on PCR repeatability [12].

The major risk of an amplification procedure is the loss of the proportional representation of T-UCR expression levels in biological samples. Previous studies reported the successful use of the SPIA™-basedprocedure in expression profiling of both eukaryotic and prokaryotic genes [6], but so far the effects of such linear amplification on non-coding protein genes have not been evaluated. We therefore assessed the potential bias of the WT-Ovation™ system by comparison of T-UCR expression in LAN-5 and GI-ME-N cell lines with or without cDNA amplification. Correlation coefficient values showed a very good linear relationship among paired amplified and non-amplified samples for highly and moderately expressed T-UCRs. The SPIA™ system thus provides a reliable representation of the most abundant T-UCR transcripts. Variation was high only for rare T-UCRs, that displayed considerable amplification bias affecting their differential expression. Our results are consistent with the influence of stochastic models, which significantly contribute to evaluate the accuracy of transcript quantification at low levels [13]. In particular, the Poisson's statistics predict the frequency distribution of the number of RNA molecules that will be present for measurement in each PCR tube. In this context, stochastic fluctuations influence the sensitivity when only a small amount of quantifiable transcripts is available. This implies that the number of measurement repetitions is relevant in the quantification of rare transcripts and that repeated measurements of a transcript at low level should exactly reflect the Poisson type of distribution. For example, with two replicates (which is the number of qPCR runs that we conducted per T-UCR) there is 86.5% chance to detect at least one positive score when the expected value is 1. Under the Poisson assumption, 8 replicates would detect the remaining 13.5% [14]. However, it is questionable that this improvement in accuracy is worth the cost. As a practical matter, it should be unrealistic to perform all necessary replicate assays to determine the exact amount of rare T-UCR transcripts, also taking into account that our analysis revealed that the trend of differential expression is always preserved for the low abundant T-UCRs.

It is noteworthy that the amplification approach allowed us to significantly reduce the amount of RNA (150 ng) required to analyze all 481 T-UCRs per each sample, preserving in the meantime the magnitude of relative expression. Therefore, the quantification procedure described here is both an accurate and reliable technique for genome-wide T-UCR profiling when minimal amounts of starting material are available. This requirement is particularly important because tumor sample size is often limited and studies of transcription regulation are increasingly conducted in small homogeneous samples, such as laser capture microdissected or sorted cell populations. It is noteworthy that the biological implications of the scientific advance presented in this manuscript have been recently published [5]. In fact, we applied the linear isothermal Ribo-SPIA™ amplification method on 34 NB biopsies and this enabled us to establish a T-UCR-expression signature predictive of outcome in non infant patients diagnosed with metastatic NB.

## Conclusions

We have demonstrated that the SPIA™ system global amplification of minute amounts of RNA prior to qPCR enables sensitive and accurate interrogation of all 481 T-UCRs, while expression data should be considered with caution for rare T-UCRs only. We strongly believe that our results open the way to use the method presented here for an accurate high-throughput profiling of non coding protein genes in those samples of potential clinical interest, such as tumor biopsies, laser microdissected tissues, and sorted cell populations, when limited RNA amounts are available.

## Abbreviations

(T-UCRs): Transcribed-Ultra Conserved Regions; (RT-qPCR): reverse transcription-quantitative real-time PCR; (NB): neuroblastoma; (Cq): quantification cycle; (SD): standard deviation.

## Authors' contributions

PS developed the study design, carried out the preparation of RNA, the quantification and assessment of transcript integrity, the RNA amplifications and reverse transcriptions, supervised the T-UCR qPCR reactions, participated in data analysis, drafted the manuscript. SS, SC, FV, CDV performed the T-UCR qPCR reactions. SB participated in data analysis and critical revision of the manuscript. GPT participated in study conception and critical revision of the manuscript. All authors read and approved the final manuscript.

## Supplementary Material

Additional file 1 **Raw Cq data**. Raw Cq values for each qPCR reaction, according to the Real-time PCR Data Markup Language (RDML). Click here for file

Additional file 2 **Tm data**. Melting temperature values for each qPCR reaction. Click here for file

Additional file 3 **MIQE checklist**. Checklist according to the Minimum Information for Publication of Quantitative Real-Time PCR Experiments. Click here for file

Additional file 4 **Figure S1**. Plots showing the correlation between the average Cq value and the standard deviation for T-UCRs in GI-ME-N cell line, using reverse transcriptase of total RNA (**A**) and amplification system (**B**). Bar plots display the mean SD value for T-UCRs with an average Cq value ranging between 15-20, >20-25, >25-30, and >30-35 cycles. Click here for file
