# Supplementary material for: Transcribed-ultra conserved region expression profiling from low-input total RNA
Source: BMC Genomics. 2010 Mar 3;11:149. doi: 10.1186/1471-2164-11-149 (PMC2838852; doi:10.1186/1471-2164-11-149)
Supplement: Additional file 2 — Tm data. Melting temperature values for each qPCR reaction. [file 1471-2164-11-149-S2.PDF]

reactionId	sampleId	targetId	Predicted product Tm (°C)	No. Measured Tm	Measured Tm (°C)
C3	GI-ME-N_amplified	TUCR1	76.4	1	76.3
C4	GI-ME-N_amplified	TUCR1	76.4	1	76.5
A3	GI-ME-N_not amplified	TUCR1	76.4	1	76.7
A4	GI-ME-N_not amplified	TUCR1	76.4	1	76.8
D4	LAN5_amplified	TUCR1	76.4	0	
D3	LAN5_amplified	TUCR1	76.4	1	76.4
B3	LAN5_not amplified	TUCR1	76.4	1	76.5
B4	LAN5_not amplified	TUCR1	76.4	1	76.7
G10	GI-ME-N_amplified	TUCR10	78.7	1	77.4
G9	GI-ME-N_amplified	TUCR10	78.7	1	77.5
E10	GI-ME-N_not amplified	TUCR10	78.7	1	77.4
E9	GI-ME-N_not amplified	TUCR10	78.7	1	77.5
H10	LAN5_amplified	TUCR10	78.7	1	77.5
H9	LAN5_amplified	TUCR10	78.7	1	77.6
F10	LAN5_not amplified	TUCR10	78.7	1	73.3
F9	LAN5_not amplified	TUCR10	78.7	1	77.5
C1	GI-ME-N_amplified	TUCR100	74.9	1	75.4
C2	GI-ME-N_amplified	TUCR100	74.9	1	75.2
A1	GI-ME-N_not amplified	TUCR100	74.9	1	75.7
A2	GI-ME-N_not amplified	TUCR100	74.9	1	75.6
D5	LAN5_amplified	TUCR100	74.9	1	75.4
D6	LAN5_amplified	TUCR100	74.9	1	75.3
B5	LAN5_not amplified	TUCR100	74.9	1	75.6
B6	LAN5_not amplified	TUCR100	74.9	1	75.5
C3	GI-ME-N_amplified	TUCR101	76.2	1	75.9
C4	GI-ME-N_amplified	TUCR101	76.2	1	76
A3	GI-ME-N_not amplified	TUCR101	76.2	1	76.3
A4	GI-ME-N_not amplified	TUCR101	76.2	1	76.4
D7	LAN5_amplified	TUCR101	76.2	1	76
D8	LAN5_amplified	TUCR101	76.2	1	76
B7	LAN5_not amplified	TUCR101	76.2	1	76.1
B8	LAN5_not amplified	TUCR101	76.2	1	76.2
C10	GI-ME-N_amplified	TUCR102	77.5	1	77.9
C9	GI-ME-N_amplified	TUCR102	77.5	1	78
A10	GI-ME-N_not amplified	TUCR102	77.5	1	78.3
A9	GI-ME-N_not amplified	TUCR102	77.5	1	78.4
D10	LAN5_amplified	TUCR102	77.5	1	77.9
D9	LAN5_amplified	TUCR102	77.5	1	78
B10	LAN5_not amplified	TUCR102	77.5	1	78
B9	LAN5_not amplified	TUCR102	77.5	1	78.2
C7	GI-ME-N_amplified	TUCR103	73.8	1	74.9
C8	GI-ME-N_amplified	TUCR103	73.8	1	75.2
A7	GI-ME-N_not amplified	TUCR103	73.8	1	75.4
A8	GI-ME-N_not amplified	TUCR103	73.8	1	75.5
D11	LAN5_amplified	TUCR103	73.8	1	74.9
D12	LAN5_amplified	TUCR103	73.8	1	75.1
B11	LAN5_not amplified	TUCR103	73.8	1	75.1
B12	LAN5_not amplified	TUCR103	73.8	1	75.3
F1	GI-ME-N_amplified	TUCR104	76.7	1	76.9
F2	GI-ME-N_amplified	TUCR104	76.7	1	76.7
E1	GI-ME-N_not amplified	TUCR104	76.7	1	76.7
E2	GI-ME-N_not amplified	TUCR104	76.7	1	76.5
G1	LAN5_amplified	TUCR104	76.7	1	76.9
G2	LAN5_amplified	TUCR104	76.7	1	76.7
H1	LAN5_not amplified	TUCR104	76.7	1	77.2
H2	LAN5_not amplified	TUCR104	76.7	1	77
F3	GI-ME-N_amplified	TUCR105	77.4	1	79.7
F4	GI-ME-N_amplified	TUCR105	77.4	1	79.6
E3	GI-ME-N_not amplified	TUCR105	77.4	0	
E4	GI-ME-N_not amplified	TUCR105	77.4	0	
G3	LAN5_amplified	TUCR105	77.4	1	79.3
G4	LAN5_amplified	TUCR105	77.4	1	79.4
H3	LAN5_not amplified	TUCR105	77.4	0	
H4	LAN5_not amplified	TUCR105	77.4	0	

G5	GI-ME-N_amplified	TUCR106	76.8	1	77.1
G6	GI-ME-N_amplified	TUCR106	76.8	1	76.9
E5	GI-ME-N_not amplified	TUCR106	76.8	1	76.9
E6	GI-ME-N_not amplified	TUCR106	76.8	1	76.9
H5	LAN5_amplified	TUCR106	76.8	1	77.3
H6	LAN5_amplified	TUCR106	76.8	1	77
F5	LAN5_not amplified	TUCR106	76.8	1	77
F6	LAN5_not amplified	TUCR106	76.8	1	76.9
G7	GI-ME-N_amplified	TUCR107	78.4	1	78.5
G8	GI-ME-N_amplified	TUCR107	78.4	1	78.4
E8	GI-ME-N_not amplified	TUCR107	78.4	0	
E7	GI-ME-N_not amplified	TUCR107	78.4	2	79.0, 82.4
H7	LAN5_amplified	TUCR107	78.4	1	77.4
H8	LAN5_amplified	TUCR107	78.4	2	74.3, 82.8
F8	LAN5_not amplified	TUCR107	78.4	0	
F7	LAN5_not amplified	TUCR107	78.4	1	82.3
G10	GI-ME-N_amplified	TUCR108	81.5	1	80.3
G9	GI-ME-N_amplified	TUCR108	81.5	1	80.5
E10	GI-ME-N_not amplified	TUCR108	81.5	1	80.3
E9	GI-ME-N_not amplified	TUCR108	81.5	1	80.5
H10	LAN5_amplified	TUCR108	81.5	0	
H9	LAN5_amplified	TUCR108	81.5	1	80.7
F10	LAN5_not amplified	TUCR108	81.5	0	
F9	LAN5_not amplified	TUCR108	81.5	1	80.5
G10	GI-ME-N_amplified	TUCR109	78	1	77
G11	GI-ME-N_amplified	TUCR109	78	1	77.2
E10	GI-ME-N_not amplified	TUCR109	78	1	77
E11	GI-ME-N_not amplified	TUCR109	78	1	77.1
H11	LAN5_amplified	TUCR109	78	1	77.2
H12	LAN5_amplified	TUCR109	78	1	77.5
F11	LAN5_not amplified	TUCR109	78	1	76.9
F12	LAN5_not amplified	TUCR109	78	1	77.2
C1	GI-ME-N_amplified	TUCR110	83.3	1	82.3
C2	GI-ME-N_amplified	TUCR110	83.3	1	82.1
A1	GI-ME-N_not amplified	TUCR110	83.3	1	82.5
A2	GI-ME-N_not amplified	TUCR110	83.3	1	82.4
D1	LAN5_amplified	TUCR110	83.3	1	82.4
D2	LAN5_amplified	TUCR110	83.3	1	82.2
B1	LAN5_not amplified	TUCR110	83.3	1	82.4
B2	LAN5_not amplified	TUCR110	83.3	1	82.1
C3	GI-ME-N_amplified	TUCR111	87.6	1	85.2
C4	GI-ME-N_amplified	TUCR111	87.6	1	85.4
A3	GI-ME-N_not amplified	TUCR111	87.6	1	85.5
A4	GI-ME-N_not amplified	TUCR111	87.6	1	85.7
D3	LAN5_amplified	TUCR111	87.6	1	85.3
D4	LAN5_amplified	TUCR111	87.6	1	85.4
B3	LAN5_not amplified	TUCR111	87.6	1	85.2
B4	LAN5_not amplified	TUCR111	87.6	1	85.5
C5	GI-ME-N_amplified	TUCR112	78.3	1	77.3
C6	GI-ME-N_amplified	TUCR112	78.3	1	77.1
A5	GI-ME-N_not amplified	TUCR112	78.3	1	77.6
A6	GI-ME-N_not amplified	TUCR112	78.3	1	77.6
D5	LAN5_amplified	TUCR112	78.3	1	77.3
D6	LAN5_amplified	TUCR112	78.3	2	77.0, 79.6
B5	LAN5_not amplified	TUCR112	78.3	1	77.4
B6	LAN5_not amplified	TUCR112	78.3	1	77.3
C7	GI-ME-N_amplified	TUCR113	80.1	1	73.9
C8	GI-ME-N_amplified	TUCR113	80.1	1	74.3
A7	GI-ME-N_not amplified	TUCR113	80.1	1	74.9
A8	GI-ME-N_not amplified	TUCR113	80.1	1	75
D7	LAN5_amplified	TUCR113	80.1	1	74.5
D8	LAN5_amplified	TUCR113	80.1	1	73.8
B7	LAN5_not amplified	TUCR113	80.1	1	74.8
B8	LAN5_not amplified	TUCR113	80.1	1	74.9
C10	GI-ME-N_amplified	TUCR114	82.1	0	

C9	GI-ME-N_amplified	TUCR114	82.1	1	81.2
A10	GI-ME-N_not amplified	TUCR114	82.1	0	
A9	GI-ME-N_not amplified	TUCR114	82.1	1	81.4
D10	LAN5_amplified	TUCR114	82.1	0	
D9	LAN5_amplified	TUCR114	82.1	1	81.1
B10	LAN5_not amplified	TUCR114	82.1	0	
B9	LAN5_not amplified	TUCR114	82.1	1	81.4
C11	GI-ME-N_amplified	TUCR115	74.8	1	74.2
C12	GI-ME-N_amplified	TUCR115	74.8	1	74.6
A11	GI-ME-N_not amplified	TUCR115	74.8	1	74.7
A12	GI-ME-N_not amplified	TUCR115	74.8	1	74.9
D11	LAN5_amplified	TUCR115	74.8	1	74.5
D12	LAN5_amplified	TUCR115	74.8	1	74.7
B11	LAN5_not amplified	TUCR115	74.8	0	
B12	LAN5_not amplified	TUCR115	74.8	0	
G1	GI-ME-N_amplified	TUCR116	78.4	1	79.5
G2	GI-ME-N_amplified	TUCR116	78.4	1	79.1
E2	GI-ME-N_not amplified	TUCR116	78.4	0	
E1	GI-ME-N_not amplified	TUCR116	78.4	1	79.3
F1	LAN5_amplified	TUCR116	78.4	1	80.8
F2	LAN5_amplified	TUCR116	78.4	1	85
H1	LAN5_not amplified	TUCR116	78.4	1	79.4
H2	LAN5_not amplified	TUCR116	78.4	1	79.1
G3	GI-ME-N_amplified	TUCR117	79.9	1	78.8
G4	GI-ME-N_amplified	TUCR117	79.9	2	75.5, 80.2
E4	GI-ME-N_not amplified	TUCR117	79.9	1	79.9
E3	GI-ME-N_not amplified	TUCR117	79.9	2	72.2, 79.9
F3	LAN5_amplified	TUCR117	79.9	0	
F4	LAN5_amplified	TUCR117	79.9	0	
H4	LAN5_not amplified	TUCR117	79.9	0	
H3	LAN5_not amplified	TUCR117	79.9	2	76.3, 78.1
G5	GI-ME-N_amplified	TUCR118	79.9	1	74.6
G6	GI-ME-N_amplified	TUCR118	79.9	2	73.8, 79.0
E5	GI-ME-N_not amplified	TUCR118	79.9	1	74.5
E6	GI-ME-N_not amplified	TUCR118	79.9	1	79
H5	LAN5_amplified	TUCR118	79.9	1	73.9
H6	LAN5_amplified	TUCR118	79.9	1	74.3
F6	LAN5_not amplified	TUCR118	79.9	0	
F5	LAN5_not amplified	TUCR118	79.9	1	74.1
G7	GI-ME-N_amplified	TUCR119	78.9	1	79.1
G8	GI-ME-N_amplified	TUCR119	78.9	1	79.1
E7	GI-ME-N_not amplified	TUCR119	78.9	1	79
E8	GI-ME-N_not amplified	TUCR119	78.9	1	79
H7	LAN5_amplified	TUCR119	78.9	1	79.3
H8	LAN5_amplified	TUCR119	78.9	1	79.4
F7	LAN5_not amplified	TUCR119	78.9	1	79
F8	LAN5_not amplified	TUCR119	78.9	1	79
G11	GI-ME-N_amplified	TUCR12	76.7	1	77.4
G12	GI-ME-N_amplified	TUCR12	76.7	1	77.5
E11	GI-ME-N_not amplified	TUCR12	76.7	1	77.4
E12	GI-ME-N_not amplified	TUCR12	76.7	1	77.5
H11	LAN5_amplified	TUCR12	76.7	1	77.5
H12	LAN5_amplified	TUCR12	76.7	1	77.6
F11	LAN5_not amplified	TUCR12	76.7	1	77.3
F12	LAN5_not amplified	TUCR12	76.7	1	77.5
G10	GI-ME-N_amplified	TUCR120	82.1	1	80.8
G9	GI-ME-N_amplified	TUCR120	82.1	1	81
E10	GI-ME-N_not amplified	TUCR120	82.1	1	80.7
E9	GI-ME-N_not amplified	TUCR120	82.1	1	80.9
H10	LAN5_amplified	TUCR120	82.1	0	
H9	LAN5_amplified	TUCR120	82.1	1	81
F10	LAN5_not amplified	TUCR120	82.1	1	80.7
F9	LAN5_not amplified	TUCR120	82.1	1	80.9
G11	GI-ME-N_amplified	TUCR121	76.5	1	75.9
G12	GI-ME-N_amplified	TUCR121	76.5	1	76.2

E11	GI-ME-N_not amplified	TUCR121	76.5	1	75.9
E12	GI-ME-N_not amplified	TUCR121	76.5	1	76.1
H11	LAN5_amplified	TUCR121	76.5	1	76.2
H12	LAN5_amplified	TUCR121	76.5	1	76.3
F11	LAN5_not amplified	TUCR121	76.5	1	75.8
F12	LAN5_not amplified	TUCR121	76.5	1	76.2
C1	GI-ME-N_amplified	TUCR122	83.6	1	82.9
C2	GI-ME-N_amplified	TUCR122	83.6	1	82.7
A1	GI-ME-N_not amplified	TUCR122	83.6	1	83.1
A2	GI-ME-N_not amplified	TUCR122	83.6	1	82.8
D1	LAN5_amplified	TUCR122	83.6	1	83.1
D2	LAN5_amplified	TUCR122	83.6	1	82.9
B1	LAN5_not amplified	TUCR122	83.6	1	83
B2	LAN5_not amplified	TUCR122	83.6	1	82.7
C3	GI-ME-N_amplified	TUCR123	76.6	1	76.8
C4	GI-ME-N_amplified	TUCR123	76.6	1	77
A3	GI-ME-N_not amplified	TUCR123	76.6	1	77.2
A4	GI-ME-N_not amplified	TUCR123	76.6	1	77.3
D3	LAN5_amplified	TUCR123	76.6	1	77.4
D4	LAN5_amplified	TUCR123	76.6	1	77.3
B3	LAN5_not amplified	TUCR123	76.6	1	77
B4	LAN5_not amplified	TUCR123	76.6	1	77.2
C5	GI-ME-N_amplified	TUCR124	81.3	1	80.6
C6	GI-ME-N_amplified	TUCR124	81.3	1	80.6
A6	GI-ME-N_not amplified	TUCR124	81.3	0	
A5	GI-ME-N_not amplified	TUCR124	81.3	1	80.7
D6	LAN5_amplified	TUCR124	81.3	0	
D5	LAN5_amplified	TUCR124	81.3	1	77.9
B5	LAN5_not amplified	TUCR124	81.3	1	80.6
B6	LAN5_not amplified	TUCR124	81.3	1	80.6
C7	GI-ME-N_amplified	TUCR125	77	0	
C8	GI-ME-N_amplified	TUCR125	77	1	75.8
A8	GI-ME-N_not amplified	TUCR125	77	0	
A7	GI-ME-N_not amplified	TUCR125	77	1	75
D7	LAN5_amplified	TUCR125	77	0	
D8	LAN5_amplified	TUCR125	77	1	80.3
B7	LAN5_not amplified	TUCR125	77	1	76.5
B8	LAN5_not amplified	TUCR125	77	1	76.6
C10	GI-ME-N_amplified	TUCR126	77.8	1	77.3
C9	GI-ME-N_amplified	TUCR126	77.8	1	77.3
A10	GI-ME-N_not amplified	TUCR126	77.8	1	77.5
A9	GI-ME-N_not amplified	TUCR126	77.8	1	77.7
D10	LAN5_amplified	TUCR126	77.8	1	77.2
D9	LAN5_amplified	TUCR126	77.8	1	77.4
B10	LAN5_not amplified	TUCR126	77.8	1	77.3
B9	LAN5_not amplified	TUCR126	77.8	1	77.5
C11	GI-ME-N_amplified	TUCR127	77.9	1	78.2
C12	GI-ME-N_amplified	TUCR127	77.9	1	78.4
A11	GI-ME-N_not amplified	TUCR127	77.9	1	78.8
A12	GI-ME-N_not amplified	TUCR127	77.9	1	75.5
D11	LAN5_amplified	TUCR127	77.9	1	78.3
D12	LAN5_amplified	TUCR127	77.9	1	78.4
B11	LAN5_not amplified	TUCR127	77.9	1	78.6
B12	LAN5_not amplified	TUCR127	77.9	2	74.9, 78.5
G1	GI-ME-N_amplified	TUCR128	76	1	77.2
G2	GI-ME-N_amplified	TUCR128	76	1	76.9
E1	GI-ME-N_not amplified	TUCR128	76	1	76.9
E2	GI-ME-N_not amplified	TUCR128	76	1	76.8
F1	LAN5_amplified	TUCR128	76	1	77.1
F2	LAN5_amplified	TUCR128	76	1	77
H1	LAN5_not amplified	TUCR128	76	1	77.4
H2	LAN5_not amplified	TUCR128	76	1	77.3
G3	GI-ME-N_amplified	TUCR129	84.9	1	84.8
G4	GI-ME-N_amplified	TUCR129	84.9	1	84.9
E3	GI-ME-N_not amplified	TUCR129	84.9	1	84.5

E4	GI-ME-N_not amplified	TUCR129	84.9	1	84.9
F3	LAN5_amplified	TUCR129	84.9	1	84.6
F4	LAN5_amplified	TUCR129	84.9	1	84.7
H3	LAN5_not amplified	TUCR129	84.9	1	85.1
H4	LAN5_not amplified	TUCR129	84.9	1	85.1
C1	GI-ME-N_amplified	TUCR13	82.1	1	82.9
C2	GI-ME-N_amplified	TUCR13	82.1	1	82.7
A1	GI-ME-N_not amplified	TUCR13	82.1	1	83.2
A2	GI-ME-N_not amplified	TUCR13	82.1	1	83.1
D1	LAN5_amplified	TUCR13	82.1	1	83.1
D2	LAN5_amplified	TUCR13	82.1	1	82.8
B1	LAN5_not amplified	TUCR13	82.1	1	83.1
B2	LAN5_not amplified	TUCR13	82.1	1	82.8
G5	GI-ME-N_amplified	TUCR130	81.4	0	
G6	GI-ME-N_amplified	TUCR130	81.4	0	
E5	GI-ME-N_not amplified	TUCR130	81.4	0	
E6	GI-ME-N_not amplified	TUCR130	81.4	0	
H5	LAN5_amplified	TUCR130	81.4	1	80.9
H6	LAN5_amplified	TUCR130	81.4	1	80.7
F5	LAN5_not amplified	TUCR130	81.4	0	
F6	LAN5_not amplified	TUCR130	81.4	0	
G7	GI-ME-N_amplified	TUCR131	81.1	1	80.8
G8	GI-ME-N_amplified	TUCR131	81.1	1	80.9
E7	GI-ME-N_not amplified	TUCR131	81.1	1	80.8
E8	GI-ME-N_not amplified	TUCR131	81.1	1	80.7
H7	LAN5_amplified	TUCR131	81.1	1	81
H8	LAN5_amplified	TUCR131	81.1	1	81.1
F7	LAN5_not amplified	TUCR131	81.1	1	80.7
F8	LAN5_not amplified	TUCR131	81.1	1	80.8
G10	GI-ME-N_amplified	TUCR132	80.9	1	79.4
G9	GI-ME-N_amplified	TUCR132	80.9	1	79.5
E10	GI-ME-N_not amplified	TUCR132	80.9	1	79.4
E9	GI-ME-N_not amplified	TUCR132	80.9	1	79.5
H10	LAN5_amplified	TUCR132	80.9	1	79.5
H9	LAN5_amplified	TUCR132	80.9	1	79.8
F10	LAN5_not amplified	TUCR132	80.9	1	79.4
F9	LAN5_not amplified	TUCR132	80.9	1	79.6
G11	GI-ME-N_amplified	TUCR133	79.5	1	78.6
G12	GI-ME-N_amplified	TUCR133	79.5	1	78.8
E11	GI-ME-N_not amplified	TUCR133	79.5	1	78.6
E12	GI-ME-N_not amplified	TUCR133	79.5	1	78.7
H11	LAN5_amplified	TUCR133	79.5	1	78.8
H12	LAN5_amplified	TUCR133	79.5	1	79
F11	LAN5_not amplified	TUCR133	79.5	1	78.5
F12	LAN5_not amplified	TUCR133	79.5	1	78.7
C1	GI-ME-N_amplified	TUCR134	79.6	1	79.3
C2	GI-ME-N_amplified	TUCR134	79.6	1	79.1
A1	GI-ME-N_not amplified	TUCR134	79.6	1	79.6
A2	GI-ME-N_not amplified	TUCR134	79.6	1	79.5
D1	LAN5_amplified	TUCR134	79.6	1	79.5
D2	LAN5_amplified	TUCR134	79.6	1	79.2
B1	LAN5_not amplified	TUCR134	79.6	1	79.5
B2	LAN5_not amplified	TUCR134	79.6	1	79.3
C3	GI-ME-N_amplified	TUCR135	77.1	1	77.6
C4	GI-ME-N_amplified	TUCR135	77.1	1	77.8
A3	GI-ME-N_not amplified	TUCR135	77.1	1	78
A4	GI-ME-N_not amplified	TUCR135	77.1	1	78.1
D3	LAN5_amplified	TUCR135	77.1	1	77.7
D4	LAN5_amplified	TUCR135	77.1	1	77.8
B3	LAN5_not amplified	TUCR135	77.1	1	77.7
B4	LAN5_not amplified	TUCR135	77.1	1	77.8
C5	GI-ME-N_amplified	TUCR136	77.3	1	76.5
C6	GI-ME-N_amplified	TUCR136	77.3	1	76.3
A5	GI-ME-N_not amplified	TUCR136	77.3	1	76.8
A6	GI-ME-N_not amplified	TUCR136	77.3	1	76.7

D5	LAN5_amplified	TUCR136	77.3	1	76.5
D6	LAN5_amplified	TUCR136	77.3	1	76.3
B6	LAN5_not amplified	TUCR136	77.3	0	
B5	LAN5_not amplified	TUCR136	77.3	1	76.7
C8	GI-ME-N_amplified	TUCR137	78.8	0	
C7	GI-ME-N_amplified	TUCR137	78.8	1	78.2
A8	GI-ME-N_not amplified	TUCR137	78.8	0	
A7	GI-ME-N_not amplified	TUCR137	78.8	1	78.6
D8	LAN5_amplified	TUCR137	78.8	0	
D7	LAN5_amplified	TUCR137	78.8	1	78.4
B7	LAN5_not amplified	TUCR137	78.8	0	
B8	LAN5_not amplified	TUCR137	78.8	0	
C10	GI-ME-N_amplified	TUCR138	76.5	1	75.7
C9	GI-ME-N_amplified	TUCR138	76.5	1	75.8
A10	GI-ME-N_not amplified	TUCR138	76.5	1	76.1
A9	GI-ME-N_not amplified	TUCR138	76.5	1	76.2
D10	LAN5_amplified	TUCR138	76.5	1	75.6
D9	LAN5_amplified	TUCR138	76.5	1	75.7
B10	LAN5_not amplified	TUCR138	76.5	1	75.8
B9	LAN5_not amplified	TUCR138	76.5	1	76
C11	GI-ME-N_amplified	TUCR139	73.3	1	73.9
C12	GI-ME-N_amplified	TUCR139	73.3	1	74.1
A11	GI-ME-N_not amplified	TUCR139	73.3	1	74.3
A12	GI-ME-N_not amplified	TUCR139	73.3	1	74.5
D11	LAN5_amplified	TUCR139	73.3	1	74
D12	LAN5_amplified	TUCR139	73.3	1	74.1
B11	LAN5_not amplified	TUCR139	73.3	1	74.1
B12	LAN5_not amplified	TUCR139	73.3	1	74.3
C3	GI-ME-N_amplified	TUCR14	84.1	1	84.3
C4	GI-ME-N_amplified	TUCR14	84.1	1	84.5
A3	GI-ME-N_not amplified	TUCR14	84.1	1	84.6
A4	GI-ME-N_not amplified	TUCR14	84.1	1	84.8
D3	LAN5_amplified	TUCR14	84.1	1	84.4
D4	LAN5_amplified	TUCR14	84.1	1	84.2
B3	LAN5_not amplified	TUCR14	84.1	1	84.4
B4	LAN5_not amplified	TUCR14	84.1	1	84.6
G1	GI-ME-N_amplified	TUCR140	76.9	1	77.8
G2	GI-ME-N_amplified	TUCR140	76.9	1	77.7
E1	GI-ME-N_not amplified	TUCR140	76.9	1	77.7
E2	GI-ME-N_not amplified	TUCR140	76.9	1	77.4
F1	LAN5_amplified	TUCR140	76.9	1	77.7
F2	LAN5_amplified	TUCR140	76.9	1	77.6
H1	LAN5_not amplified	TUCR140	76.9	1	78.1
H2	LAN5_not amplified	TUCR140	76.9	1	77.9
G3	GI-ME-N_amplified	TUCR141	78	1	78
G4	GI-ME-N_amplified	TUCR141	78	1	78.1
E3	GI-ME-N_not amplified	TUCR141	78	1	77.7
E4	GI-ME-N_not amplified	TUCR141	78	1	77.7
F3	LAN5_amplified	TUCR141	78	1	77.8
F4	LAN5_amplified	TUCR141	78	1	77.9
H3	LAN5_not amplified	TUCR141	78	1	78.4
H4	LAN5_not amplified	TUCR141	78	1	78.2
G5	GI-ME-N_amplified	TUCR142	79.5	1	80.3
G6	GI-ME-N_amplified	TUCR142	79.5	1	80
E5	GI-ME-N_not amplified	TUCR142	79.5	1	80.2
E6	GI-ME-N_not amplified	TUCR142	79.5	1	80
H5	LAN5_amplified	TUCR142	79.5	1	80.5
H6	LAN5_amplified	TUCR142	79.5	1	80.3
F5	LAN5_not amplified	TUCR142	79.5	1	80.2
F6	LAN5_not amplified	TUCR142	79.5	1	80
G7	GI-ME-N_amplified	TUCR143	83.9	1	82
G8	GI-ME-N_amplified	TUCR143	83.9	1	82.1
E7	GI-ME-N_not amplified	TUCR143	83.9	1	81.9
E8	GI-ME-N_not amplified	TUCR143	83.9	1	81.9
H7	LAN5_amplified	TUCR143	83.9	1	82.2

H8	LAN5_amplified	TUCR143	83.9	1	82.3
F7	LAN5_not amplified	TUCR143	83.9	1	81.9
F8	LAN5_not amplified	TUCR143	83.9	1	82
G10	GI-ME-N_amplified	TUCR144	87.4	1	87
G9	GI-ME-N_amplified	TUCR144	87.4	1	87.3
E10	GI-ME-N_not amplified	TUCR144	87.4	1	86.9
E9	GI-ME-N_not amplified	TUCR144	87.4	1	87.1
H10	LAN5_amplified	TUCR144	87.4	1	87.3
H9	LAN5_amplified	TUCR144	87.4	1	87.5
F10	LAN5_not amplified	TUCR144	87.4	1	86.9
F9	LAN5_not amplified	TUCR144	87.4	1	87.1
G11	GI-ME-N_amplified	TUCR145	80.2	1	79.4
G12	GI-ME-N_amplified	TUCR145	80.2	1	79.6
E11	GI-ME-N_not amplified	TUCR145	80.2	1	79.6
E12	GI-ME-N_not amplified	TUCR145	80.2	1	79.6
H12	LAN5_amplified	TUCR145	80.2	0	
H11	LAN5_amplified	TUCR145	80.2	1	79.6
F11	LAN5_not amplified	TUCR145	80.2	0	
F12	LAN5_not amplified	TUCR145	80.2	1	76
C2	GI-ME-N_amplified	TUCR146	74.6	0	
C1	GI-ME-N_amplified	TUCR146	74.6	1	74.5
A1	GI-ME-N_not amplified	TUCR146	74.6	1	74.8
A2	GI-ME-N_not amplified	TUCR146	74.6	1	74.6
D2	LAN5_amplified	TUCR146	74.6	0	
D1	LAN5_amplified	TUCR146	74.6	1	74.5
B2	LAN5_not amplified	TUCR146	74.6	0	
B1	LAN5_not amplified	TUCR146	74.6	1	73.4
C3	GI-ME-N_amplified	TUCR147	79.8	1	79.5
C4	GI-ME-N_amplified	TUCR147	79.8	1	79.6
A3	GI-ME-N_not amplified	TUCR147	79.8	1	79.8
A4	GI-ME-N_not amplified	TUCR147	79.8	1	79.9
D4	LAN5_amplified	TUCR147	79.8	0	
D3	LAN5_amplified	TUCR147	79.8	1	79.5
B3	LAN5_not amplified	TUCR147	79.8	1	79.5
B4	LAN5_not amplified	TUCR147	79.8	1	79.7
C5	GI-ME-N_amplified	TUCR148	84.5	1	83.7
C6	GI-ME-N_amplified	TUCR148	84.5	1	83.6
A5	GI-ME-N_not amplified	TUCR148	84.5	1	84
A6	GI-ME-N_not amplified	TUCR148	84.5	1	83.9
D5	LAN5_amplified	TUCR148	84.5	1	83.7
D6	LAN5_amplified	TUCR148	84.5	1	83.6
B5	LAN5_not amplified	TUCR148	84.5	1	83.8
B6	LAN5_not amplified	TUCR148	84.5	1	83.6
C7	GI-ME-N_amplified	TUCR149	81.9	1	81
C8	GI-ME-N_amplified	TUCR149	81.9	1	81.2
A7	GI-ME-N_not amplified	TUCR149	81.9	1	81.4
A8	GI-ME-N_not amplified	TUCR149	81.9	1	81.4
D7	LAN5_amplified	TUCR149	81.9	1	81
D8	LAN5_amplified	TUCR149	81.9	1	81.1
B7	LAN5_not amplified	TUCR149	81.9	1	81.1
B8	LAN5_not amplified	TUCR149	81.9	1	81.3
C6	GI-ME-N_amplified	TUCR15	81.9	0	
C5	GI-ME-N_amplified	TUCR15	81.9	1	81.5
A5	GI-ME-N_not amplified	TUCR15	81.9	1	82.2
A6	GI-ME-N_not amplified	TUCR15	81.9	1	82.1
D6	LAN5_amplified	TUCR15	81.9	0	
D5	LAN5_amplified	TUCR15	81.9	1	81.9
B6	LAN5_not amplified	TUCR15	81.9	0	
B5	LAN5_not amplified	TUCR15	81.9	1	82
C10	GI-ME-N_amplified	TUCR150	80.3	1	79.1
C9	GI-ME-N_amplified	TUCR150	80.3	1	79.3
A10	GI-ME-N_not amplified	TUCR150	80.3	1	79.5
A9	GI-ME-N_not amplified	TUCR150	80.3	1	79.7
D9	LAN5_amplified	TUCR150	80.3	0	
D10	LAN5_amplified	TUCR150	80.3	1	77.9

B10	LAN5_not amplified	TUCR150	80.3	0	
B9	LAN5_not amplified	TUCR150	80.3	0	
C11	GI-ME-N_amplified	TUCR151	81.3	1	81.7
C12	GI-ME-N_amplified	TUCR151	81.3	1	81.9
A11	GI-ME-N_not amplified	TUCR151	81.3	1	82.1
A12	GI-ME-N_not amplified	TUCR151	81.3	1	82.1
D11	LAN5_amplified	TUCR151	81.3	1	81.6
D12	LAN5_amplified	TUCR151	81.3	1	81.9
B11	LAN5_not amplified	TUCR151	81.3	1	81.8
B12	LAN5_not amplified	TUCR151	81.3	1	82
G1	GI-ME-N_amplified	TUCR152	75	1	75.2
G2	GI-ME-N_amplified	TUCR152	75	1	75
E1	GI-ME-N_not amplified	TUCR152	75	1	74.8
E2	GI-ME-N_not amplified	TUCR152	75	1	74.6
F1	LAN5_amplified	TUCR152	75	1	75
F2	LAN5_amplified	TUCR152	75	1	74.8
H1	LAN5_not amplified	TUCR152	75	1	75.3
H2	LAN5_not amplified	TUCR152	75	1	75.2
G3	GI-ME-N_amplified	TUCR153	82.2	1	82.1
G4	GI-ME-N_amplified	TUCR153	82.2	1	82.1
E3	GI-ME-N_not amplified	TUCR153	82.2	1	81.7
E4	GI-ME-N_not amplified	TUCR153	82.2	1	81.7
F3	LAN5_amplified	TUCR153	82.2	1	81.8
F4	LAN5_amplified	TUCR153	82.2	1	81.9
H3	LAN5_not amplified	TUCR153	82.2	1	82.3
H4	LAN5_not amplified	TUCR153	82.2	1	82.3
G5	GI-ME-N_amplified	TUCR154	77.9	1	77.3
G6	GI-ME-N_amplified	TUCR154	77.9	1	77.1
E5	GI-ME-N_not amplified	TUCR154	77.9	1	77.2
E6	GI-ME-N_not amplified	TUCR154	77.9	1	77.1
H5	LAN5_amplified	TUCR154	77.9	1	77.4
H6	LAN5_amplified	TUCR154	77.9	1	77.3
F5	LAN5_not amplified	TUCR154	77.9	1	77.2
F6	LAN5_not amplified	TUCR154	77.9	1	77.1
G7	GI-ME-N_amplified	TUCR155	77.7	1	77.6
G8	GI-ME-N_amplified	TUCR155	77.7	1	75.8
E7	GI-ME-N_not amplified	TUCR155	77.7	1	77.8
E8	GI-ME-N_not amplified	TUCR155	77.7	1	75.3
H7	LAN5_amplified	TUCR155	77.7	1	77.4
H8	LAN5_amplified	TUCR155	77.7	1	77.2
F7	LAN5_not amplified	TUCR155	77.7	0	
F8	LAN5_not amplified	TUCR155	77.7	0	
G10	GI-ME-N_amplified	TUCR156	73.2	0	
G9	GI-ME-N_amplified	TUCR156	73.2	1	74.1
E10	GI-ME-N_not amplified	TUCR156	73.2	1	74.3
E9	GI-ME-N_not amplified	TUCR156	73.2	1	74.6
H10	LAN5_amplified	TUCR156	73.2	0	
H9	LAN5_amplified	TUCR156	73.2	1	73.2
F10	LAN5_not amplified	TUCR156	73.2	1	74.2
F9	LAN5_not amplified	TUCR156	73.2	1	74.6
G11	GI-ME-N_amplified	TUCR157	82.2	0	
G12	GI-ME-N_amplified	TUCR157	82.2	0	
E11	GI-ME-N_not amplified	TUCR157	82.2	0	
E12	GI-ME-N_not amplified	TUCR157	82.2	0	
H12	LAN5_amplified	TUCR157	82.2	0	
H11	LAN5_amplified	TUCR157	82.2	3	
F11	LAN5_not amplified	TUCR157	82.2	0	
F12	LAN5_not amplified	TUCR157	82.2	0	
C1	GI-ME-N_amplified	TUCR158	80.9	1	78.9
C2	GI-ME-N_amplified	TUCR158	80.9	1	80
A1	GI-ME-N_not amplified	TUCR158	80.9	0	
A2	GI-ME-N_not amplified	TUCR158	80.9	0	
D1	LAN5_amplified	TUCR158	80.9	0	
D2	LAN5_amplified	TUCR158	80.9	1	77.6
B2	LAN5_not amplified	TUCR158	80.9	0	

B1	LAN5_not amplified	TUCR158	80.9	1	80.5
C3	GI-ME-N_amplified	TUCR159	79.6	1	79
C4	GI-ME-N_amplified	TUCR159	79.6	1	79.2
A3	GI-ME-N_not amplified	TUCR159	79.6	1	79.3
A4	GI-ME-N_not amplified	TUCR159	79.6	1	75.2
D4	LAN5_amplified	TUCR159	79.6	0	
D3	LAN5_amplified	TUCR159	79.6	1	79.5
B3	LAN5_not amplified	TUCR159	79.6	1	79.2
B4	LAN5_not amplified	TUCR159	79.6	1	74.9
C8	GI-ME-N_amplified	TUCR16	79.7	0	
C7	GI-ME-N_amplified	TUCR16	79.7	1	79.2
A7	GI-ME-N_not amplified	TUCR16	79.7	0	
A8	GI-ME-N_not amplified	TUCR16	79.7	0	
D7	LAN5_amplified	TUCR16	79.7	0	
D8	LAN5_amplified	TUCR16	79.7	0	
B7	LAN5_not amplified	TUCR16	79.7	0	
B8	LAN5_not amplified	TUCR16	79.7	0	
C5	GI-ME-N_amplified	TUCR160	83	1	86.6
C6	GI-ME-N_amplified	TUCR160	83	1	86.2
A5	GI-ME-N_not amplified	TUCR160	83	1	82.7
A6	GI-ME-N_not amplified	TUCR160	83	1	82.6
D6	LAN5_amplified	TUCR160	83	0	
D5	LAN5_amplified	TUCR160	83	1	86.7
B5	LAN5_not amplified	TUCR160	83	1	82.4
B6	LAN5_not amplified	TUCR160	83	1	82.3
C7	GI-ME-N_amplified	TUCR161	83.3	1	81.7
C8	GI-ME-N_amplified	TUCR161	83.3	1	81.8
A7	GI-ME-N_not amplified	TUCR161	83.3	1	82
A8	GI-ME-N_not amplified	TUCR161	83.3	1	82.1
D7	LAN5_amplified	TUCR161	83.3	1	81.7
D8	LAN5_amplified	TUCR161	83.3	1	81.7
B7	LAN5_not amplified	TUCR161	83.3	1	81.8
B8	LAN5_not amplified	TUCR161	83.3	1	82
C10	GI-ME-N_amplified	TUCR162	83.4	1	81.4
C9	GI-ME-N_amplified	TUCR162	83.4	1	81.5
A10	GI-ME-N_not amplified	TUCR162	83.4	1	76.2
A9	GI-ME-N_not amplified	TUCR162	83.4	1	76.4
D10	LAN5_amplified	TUCR162	83.4	1	81
D9	LAN5_amplified	TUCR162	83.4	1	81.2
B10	LAN5_not amplified	TUCR162	83.4	1	81.2
B9	LAN5_not amplified	TUCR162	83.4	1	81.4
C12	GI-ME-N_amplified	TUCR163	81.6	0	
C11	GI-ME-N_amplified	TUCR163	81.6	1	81.6
A11	GI-ME-N_not amplified	TUCR163	81.6	1	80.8
A12	GI-ME-N_not amplified	TUCR163	81.6	1	69.7
D11	LAN5_amplified	TUCR163	81.6	1	80.4
D12	LAN5_amplified	TUCR163	81.6	1	77.6
B11	LAN5_not amplified	TUCR163	81.6	0	
B12	LAN5_not amplified	TUCR163	81.6	1	69.5
G1	GI-ME-N_amplified	TUCR164	79	1	79
G2	GI-ME-N_amplified	TUCR164	79	1	78.8
E1	GI-ME-N_not amplified	TUCR164	79	1	78.7
E2	GI-ME-N_not amplified	TUCR164	79	1	74.6
F1	LAN5_amplified	TUCR164	79	1	78.9
F2	LAN5_amplified	TUCR164	79	2	75.3, 78.8
H1	LAN5_not amplified	TUCR164	79	1	75.3
H2	LAN5_not amplified	TUCR164	79	1	75.1
G3	GI-ME-N_amplified	TUCR165	73.1	1	73.5
G4	GI-ME-N_amplified	TUCR165	73.1	1	73.6
E3	GI-ME-N_not amplified	TUCR165	73.1	1	73.4
E4	GI-ME-N_not amplified	TUCR165	73.1	1	73.4
F3	LAN5_amplified	TUCR165	73.1	1	73.2
F4	LAN5_amplified	TUCR165	73.1	1	73.5
H3	LAN5_not amplified	TUCR165	73.1	1	73.7
H4	LAN5_not amplified	TUCR165	73.1	1	73.7

G5	GI-ME-N_amplified	TUCR166	79.5	1	79.2
G6	GI-ME-N_amplified	TUCR166	79.5	1	78.9
E5	GI-ME-N_not amplified	TUCR166	79.5	1	79
E6	GI-ME-N_not amplified	TUCR166	79.5	1	78.8
H5	LAN5_amplified	TUCR166	79.5	1	79.3
H6	LAN5_amplified	TUCR166	79.5	1	79.1
F5	LAN5_not amplified	TUCR166	79.5	1	79
F6	LAN5_not amplified	TUCR166	79.5	1	78.9
G7	GI-ME-N_amplified	TUCR167	76.8	1	76.6
G8	GI-ME-N_amplified	TUCR167	76.8	1	76.7
E7	GI-ME-N_not amplified	TUCR167	76.8	1	76.6
E8	GI-ME-N_not amplified	TUCR167	76.8	1	76.6
H7	LAN5_amplified	TUCR167	76.8	1	76.8
H8	LAN5_amplified	TUCR167	76.8	1	76.8
F7	LAN5_not amplified	TUCR167	76.8	1	76.6
F8	LAN5_not amplified	TUCR167	76.8	1	76.7
G10	GI-ME-N_amplified	TUCR168	79.4	1	78.4
G9	GI-ME-N_amplified	TUCR168	79.4	1	78.6
E10	GI-ME-N_not amplified	TUCR168	79.4	1	78.4
E9	GI-ME-N_not amplified	TUCR168	79.4	1	78.5
H10	LAN5_amplified	TUCR168	79.4	1	78.6
H9	LAN5_amplified	TUCR168	79.4	1	78.8
F10	LAN5_not amplified	TUCR168	79.4	1	78.4
F9	LAN5_not amplified	TUCR168	79.4	1	78.6
G11	GI-ME-N_amplified	TUCR169	86.4	1	85.4
G12	GI-ME-N_amplified	TUCR169	86.4	1	85.7
E11	GI-ME-N_not amplified	TUCR169	86.4	1	85.3
E12	GI-ME-N_not amplified	TUCR169	86.4	1	85.4
H11	LAN5_amplified	TUCR169	86.4	1	85.7
H12	LAN5_amplified	TUCR169	86.4	1	85.8
F11	LAN5_not amplified	TUCR169	86.4	1	85.3
F12	LAN5_not amplified	TUCR169	86.4	1	85.6
C10	GI-ME-N_amplified	TUCR17	80.6	0	
C9	GI-ME-N_amplified	TUCR17	80.6	1	79.6
A10	GI-ME-N_not amplified	TUCR17	80.6	0	
A9	GI-ME-N_not amplified	TUCR17	80.6	0	
D10	LAN5_amplified	TUCR17	80.6	0	
D9	LAN5_amplified	TUCR17	80.6	1	74.1
B10	LAN5_not amplified	TUCR17	80.6	0	
B9	LAN5_not amplified	TUCR17	80.6	0	
C1	GI-ME-N_amplified	TUCR170	77.6	1	77.6
C2	GI-ME-N_amplified	TUCR170	77.6	1	77.3
A1	GI-ME-N_not amplified	TUCR170	77.6	1	77.7
A2	GI-ME-N_not amplified	TUCR170	77.6	1	77.5
D1	LAN5_amplified	TUCR170	77.6	1	77.6
D2	LAN5_amplified	TUCR170	77.6	1	77.5
B1	LAN5_not amplified	TUCR170	77.6	1	77.6
B2	LAN5_not amplified	TUCR170	77.6	1	77.4
C3	GI-ME-N_amplified	TUCR171	80.3	1	79.6
C4	GI-ME-N_amplified	TUCR171	80.3	1	79.7
A3	GI-ME-N_not amplified	TUCR171	80.3	1	79.9
A4	GI-ME-N_not amplified	TUCR171	80.3	1	80
D3	LAN5_amplified	TUCR171	80.3	1	79.6
D4	LAN5_amplified	TUCR171	80.3	1	79.8
B3	LAN5_not amplified	TUCR171	80.3	1	79.7
B4	LAN5_not amplified	TUCR171	80.3	1	79.8
C5	GI-ME-N_amplified	TUCR172	76.1	1	76.5
C6	GI-ME-N_amplified	TUCR172	76.1	1	76.4
A5	GI-ME-N_not amplified	TUCR172	76.1	1	76.8
A6	GI-ME-N_not amplified	TUCR172	76.1	1	76.7
D5	LAN5_amplified	TUCR172	76.1	1	76.5
D6	LAN5_amplified	TUCR172	76.1	1	76.5
B5	LAN5_not amplified	TUCR172	76.1	1	76.7
B6	LAN5_not amplified	TUCR172	76.1	1	76.5
C7	GI-ME-N_amplified	TUCR173	77.8	1	76.8

C8	GI-ME-N_amplified	TUCR173	77.8	1	76.9
A7	GI-ME-N_not amplified	TUCR173	77.8	1	77.2
A8	GI-ME-N_not amplified	TUCR173	77.8	1	77.2
D7	LAN5_amplified	TUCR173	77.8	1	76.8
D8	LAN5_amplified	TUCR173	77.8	1	76.9
B7	LAN5_not amplified	TUCR173	77.8	1	77
B8	LAN5_not amplified	TUCR173	77.8	1	77.1
C10	GI-ME-N_amplified	TUCR174	79.8	1	78
C9	GI-ME-N_amplified	TUCR174	79.8	1	78.1
A10	GI-ME-N_not amplified	TUCR174	79.8	1	78.4
A9	GI-ME-N_not amplified	TUCR174	79.8	1	78.6
D10	LAN5_amplified	TUCR174	79.8	1	78
D9	LAN5_amplified	TUCR174	79.8	1	78.1
B10	LAN5_not amplified	TUCR174	79.8	1	78.2
B9	LAN5_not amplified	TUCR174	79.8	1	78.4
C11	GI-ME-N_amplified	TUCR175	75.7	1	74.9
C12	GI-ME-N_amplified	TUCR175	75.7	1	75.1
A11	GI-ME-N_not amplified	TUCR175	75.7	1	75.3
A12	GI-ME-N_not amplified	TUCR175	75.7	1	75.4
D11	LAN5_amplified	TUCR175	75.7	1	74.9
D12	LAN5_amplified	TUCR175	75.7	1	75
B11	LAN5_not amplified	TUCR175	75.7	1	75.1
B12	LAN5_not amplified	TUCR175	75.7	1	75.2
G1	GI-ME-N_amplified	TUCR176	76.4	1	77.5
G2	GI-ME-N_amplified	TUCR176	76.4	1	77.3
E1	GI-ME-N_not amplified	TUCR176	76.4	1	77.2
E2	GI-ME-N_not amplified	TUCR176	76.4	1	77
F1	LAN5_amplified	TUCR176	76.4	1	77.3
F2	LAN5_amplified	TUCR176	76.4	1	77.3
H2	LAN5_not amplified	TUCR176	76.4	0	
H1	LAN5_not amplified	TUCR176	76.4	1	77.6
G3	GI-ME-N_amplified	TUCR177	77.1	1	76.4
G4	GI-ME-N_amplified	TUCR177	77.1	1	76.5
E3	GI-ME-N_not amplified	TUCR177	77.1	1	76.3
E4	GI-ME-N_not amplified	TUCR177	77.1	1	76.2
F3	LAN5_amplified	TUCR177	77.1	1	76.4
F4	LAN5_amplified	TUCR177	77.1	1	76.5
H3	LAN5_not amplified	TUCR177	77.1	1	76.7
H4	LAN5_not amplified	TUCR177	77.1	1	76.7
G5	GI-ME-N_amplified	TUCR178	74.9	1	75.2
G6	GI-ME-N_amplified	TUCR178	74.9	1	75
E5	GI-ME-N_not amplified	TUCR178	74.9	1	74.9
E6	GI-ME-N_not amplified	TUCR178	74.9	1	74.9
H5	LAN5_amplified	TUCR178	74.9	1	75.3
H6	LAN5_amplified	TUCR178	74.9	1	75.2
F5	LAN5_not amplified	TUCR178	74.9	1	75
F6	LAN5_not amplified	TUCR178	74.9	1	74.9
G7	GI-ME-N_amplified	TUCR179	82.2	1	81
G8	GI-ME-N_amplified	TUCR179	82.2	1	81.1
E7	GI-ME-N_not amplified	TUCR179	82.2	1	80.8
E8	GI-ME-N_not amplified	TUCR179	82.2	1	80.9
H7	LAN5_amplified	TUCR179	82.2	1	81.3
H8	LAN5_amplified	TUCR179	82.2	1	81.3
F7	LAN5_not amplified	TUCR179	82.2	1	80.9
F8	LAN5_not amplified	TUCR179	82.2	1	81
C11	GI-ME-N_amplified	TUCR18	80.2	1	79.2
C12	GI-ME-N_amplified	TUCR18	80.2	1	79.5
A11	GI-ME-N_not amplified	TUCR18	80.2	1	79.7
A12	GI-ME-N_not amplified	TUCR18	80.2	1	79.8
D11	LAN5_amplified	TUCR18	80.2	1	79.3
D12	LAN5_amplified	TUCR18	80.2	1	79.3
B11	LAN5_not amplified	TUCR18	80.2	1	79.5
B12	LAN5_not amplified	TUCR18	80.2	1	79.6
G10	GI-ME-N_amplified	TUCR180	82	1	80.6
G9	GI-ME-N_amplified	TUCR180	82	1	80.8

E10	GI-ME-N_not amplified	TUCR180	82	1	80.6
E9	GI-ME-N_not amplified	TUCR180	82	1	80.7
H10	LAN5_amplified	TUCR180	82	1	80.8
H9	LAN5_amplified	TUCR180	82	1	80.9
F10	LAN5_not amplified	TUCR180	82	1	80.5
F9	LAN5_not amplified	TUCR180	82	1	80.8
G11	GI-ME-N_amplified	TUCR181	82.4	1	81.4
G12	GI-ME-N_amplified	TUCR181	82.4	1	81.6
E11	GI-ME-N_not amplified	TUCR181	82.4	1	81.4
E12	GI-ME-N_not amplified	TUCR181	82.4	1	81.5
H11	LAN5_amplified	TUCR181	82.4	1	81.6
H12	LAN5_amplified	TUCR181	82.4	1	81.7
F11	LAN5_not amplified	TUCR181	82.4	1	81.3
F12	LAN5_not amplified	TUCR181	82.4	1	81.5
C2	GI-ME-N_amplified	TUCR182	77.4	0	
C1	GI-ME-N_amplified	TUCR182	77.4	1	77.6
A2	GI-ME-N_not amplified	TUCR182	77.4	0	
A1	GI-ME-N_not amplified	TUCR182	77.4	1	77.7
D2	LAN5_amplified	TUCR182	77.4	0	
D1	LAN5_amplified	TUCR182	77.4	1	77.6
B2	LAN5_not amplified	TUCR182	77.4	0	
B1	LAN5_not amplified	TUCR182	77.4	1	77.6
C3	GI-ME-N_amplified	TUCR183	76.4	1	79.6
C4	GI-ME-N_amplified	TUCR183	76.4	1	79.8
A4	GI-ME-N_not amplified	TUCR183	76.4	0	
A3	GI-ME-N_not amplified	TUCR183	76.4	1	79.6
D4	LAN5_amplified	TUCR183	76.4	0	
D3	LAN5_amplified	TUCR183	76.4	1	79.7
B3	LAN5_not amplified	TUCR183	76.4	1	79.9
B4	LAN5_not amplified	TUCR183	76.4	1	80
C5	GI-ME-N_amplified	TUCR184	76.4	1	76.5
C6	GI-ME-N_amplified	TUCR184	76.4	1	76.4
A6	GI-ME-N_not amplified	TUCR184	76.4	0	
A5	GI-ME-N_not amplified	TUCR184	76.4	1	76.8
D5	LAN5_amplified	TUCR184	76.4	1	76.5
D6	LAN5_amplified	TUCR184	76.4	1	76.5
B5	LAN5_not amplified	TUCR184	76.4	1	76.7
B6	LAN5_not amplified	TUCR184	76.4	1	76.5
C7	GI-ME-N_amplified	TUCR185	75	1	76.8
C8	GI-ME-N_amplified	TUCR185	75	1	76.9
A7	GI-ME-N_not amplified	TUCR185	75	1	77.2
A8	GI-ME-N_not amplified	TUCR185	75	1	77.2
D7	LAN5_amplified	TUCR185	75	1	76.8
D8	LAN5_amplified	TUCR185	75	1	76.9
B7	LAN5_not amplified	TUCR185	75	1	77
B8	LAN5_not amplified	TUCR185	75	1	77.1
C10	GI-ME-N_amplified	TUCR186	75.6	1	78
C9	GI-ME-N_amplified	TUCR186	75.6	1	78.1
A10	GI-ME-N_not amplified	TUCR186	75.6	1	78.4
A9	GI-ME-N_not amplified	TUCR186	75.6	1	78.6
D10	LAN5_amplified	TUCR186	75.6	1	78
D9	LAN5_amplified	TUCR186	75.6	1	78.1
B10	LAN5_not amplified	TUCR186	75.6	1	78.2
B9	LAN5_not amplified	TUCR186	75.6	1	78.4
A11	GI-ME-N_not amplified	TUCR187	78.4	1	75.3
D11	LAN5_amplified	TUCR187	78.4	1	74.9
D12	LAN5_amplified	TUCR187	78.4	1	75
B11	LAN5_not amplified	TUCR187	78.4	1	75.1
B12	LAN5_not amplified	TUCR187	78.4	1	75.2
F1	GI-ME-N_amplified	TUCR188	74.4	1	77.3
F2	GI-ME-N_amplified	TUCR188	74.4	1	77.5
E1	GI-ME-N_not amplified	TUCR188	74.4	1	77.2
E2	GI-ME-N_not amplified	TUCR188	74.4	1	77
G1	LAN5_amplified	TUCR188	74.4	0	
G2	LAN5_amplified	TUCR188	74.4	1	77.3

H1	LAN5_not amplified	TUCR188	74.4	1	77.6
H2	LAN5_not amplified	TUCR188	74.4	1	77.5
E3	GI-ME-N_amplified	TUCR189	77	1	76.3
E4	GI-ME-N_amplified	TUCR189	77	1	76.2
G3	GI-ME-N_not amplified	TUCR189	77	1	76.4
G4	GI-ME-N_not amplified	TUCR189	77	1	76.5
F3	LAN5_amplified	TUCR189	77	1	76.4
F4	LAN5_amplified	TUCR189	77	1	76.5
H3	LAN5_not amplified	TUCR189	77	1	76.7
H4	LAN5_not amplified	TUCR189	77	1	76.7
G1	GI-ME-N_amplified	TUCR19	78.1	1	78.9
G2	GI-ME-N_amplified	TUCR19	78.1	1	78.6
E1	GI-ME-N_not amplified	TUCR19	78.1	1	78.3
E2	GI-ME-N_not amplified	TUCR19	78.1	1	78.1
F1	LAN5_amplified	TUCR19	78.1	0	
F2	LAN5_amplified	TUCR19	78.1	0	
H1	LAN5_not amplified	TUCR19	78.1	0	
H2	LAN5_not amplified	TUCR19	78.1	0	
G5	GI-ME-N_amplified	TUCR190	71.5	1	75.2
G6	GI-ME-N_amplified	TUCR190	71.5	1	75
E5	GI-ME-N_not amplified	TUCR190	71.5	1	74.9
E6	GI-ME-N_not amplified	TUCR190	71.5	1	74.9
H5	LAN5_amplified	TUCR190	71.5	1	75.3
H6	LAN5_amplified	TUCR190	71.5	1	75.2
F5	LAN5_not amplified	TUCR190	71.5	1	75
F6	LAN5_not amplified	TUCR190	71.5	1	74.9
G7	GI-ME-N_amplified	TUCR191	80.4	1	81
G8	GI-ME-N_amplified	TUCR191	80.4	1	81.1
E7	GI-ME-N_not amplified	TUCR191	80.4	1	80.8
E8	GI-ME-N_not amplified	TUCR191	80.4	1	80.9
H7	LAN5_amplified	TUCR191	80.4	1	81.3
H8	LAN5_amplified	TUCR191	80.4	1	81.3
F8	LAN5_not amplified	TUCR191	80.4	0	
F7	LAN5_not amplified	TUCR191	80.4	1	80.9
G10	GI-ME-N_amplified	TUCR192	76.9	0	
G9	GI-ME-N_amplified	TUCR192	76.9	1	80.8
E10	GI-ME-N_not amplified	TUCR192	76.9	1	80.6
E9	GI-ME-N_not amplified	TUCR192	76.9	1	80.7
H10	LAN5_amplified	TUCR192	76.9	1	80.8
H9	LAN5_amplified	TUCR192	76.9	1	80.9
F10	LAN5_not amplified	TUCR192	76.9	1	80.5
F9	LAN5_not amplified	TUCR192	76.9	1	80.8
G11	GI-ME-N_amplified	TUCR193	78.7	1	81.4
G12	GI-ME-N_amplified	TUCR193	78.7	1	81.6
E11	GI-ME-N_not amplified	TUCR193	78.7	1	81.4
E12	GI-ME-N_not amplified	TUCR193	78.7	1	81.5
H12	LAN5_amplified	TUCR193	78.7	0	
H11	LAN5_amplified	TUCR193	78.7	1	81.6
F12	LAN5_not amplified	TUCR193	78.7	0	
F11	LAN5_not amplified	TUCR193	78.7	1	81.3
C1	GI-ME-N_amplified	TUCR194	77.8	1	78.3
C2	GI-ME-N_amplified	TUCR194	77.8	1	78.1
A1	GI-ME-N_not amplified	TUCR194	77.8	1	78.5
A2	GI-ME-N_not amplified	TUCR194	77.8	1	78.4
D2	LAN5_amplified	TUCR194	77.8	0	
D1	LAN5_amplified	TUCR194	77.8	1	80.7
B1	LAN5_not amplified	TUCR194	77.8	1	77
B2	LAN5_not amplified	TUCR194	77.8	1	77.8
C3	GI-ME-N_amplified	TUCR195	82	1	81.3
C4	GI-ME-N_amplified	TUCR195	82	1	81.4
A3	GI-ME-N_not amplified	TUCR195	82	1	81.7
A4	GI-ME-N_not amplified	TUCR195	82	1	81.8
D3	LAN5_amplified	TUCR195	82	1	81.4
D4	LAN5_amplified	TUCR195	82	1	81.5
B3	LAN5_not amplified	TUCR195	82	1	81.4

B4	LAN5_not amplified	TUCR195	82	1	81.6
C5	GI-ME-N_amplified	TUCR196	79.6	1	79.8
C6	GI-ME-N_amplified	TUCR196	79.6	1	79.6
A5	GI-ME-N_not amplified	TUCR196	79.6	1	80.1
A6	GI-ME-N_not amplified	TUCR196	79.6	1	80
D5	LAN5_amplified	TUCR196	79.6	1	79.9
D6	LAN5_amplified	TUCR196	79.6	1	75.7
B5	LAN5_not amplified	TUCR196	79.6	1	79.9
B6	LAN5_not amplified	TUCR196	79.6	1	73.5
C7	GI-ME-N_amplified	TUCR197	74.7	1	72.7
C8	GI-ME-N_amplified	TUCR197	74.7	1	71.7
A7	GI-ME-N_not amplified	TUCR197	74.7	1	75.3
A8	GI-ME-N_not amplified	TUCR197	74.7	1	75.4
D7	LAN5_amplified	TUCR197	74.7	1	72.1
D8	LAN5_amplified	TUCR197	74.7	1	72.2
B7	LAN5_not amplified	TUCR197	74.7	1	72.2
B8	LAN5_not amplified	TUCR197	74.7	2	72.4, 75.6
C10	GI-ME-N_amplified	TUCR198	83.7	1	83.9
C9	GI-ME-N_amplified	TUCR198	83.7	1	84
A10	GI-ME-N_not amplified	TUCR198	83.7	1	74.8
A9	GI-ME-N_not amplified	TUCR198	83.7	1	75.3
D10	LAN5_amplified	TUCR198	83.7	0	
D9	LAN5_amplified	TUCR198	83.7	1	83.8
B10	LAN5_not amplified	TUCR198	83.7	1	74.9
B9	LAN5_not amplified	TUCR198	83.7	1	75.2
C11	GI-ME-N_amplified	TUCR199	75	1	76.3
C12	GI-ME-N_amplified	TUCR199	75	1	76.5
A11	GI-ME-N_not amplified	TUCR199	75	1	76.6
A12	GI-ME-N_not amplified	TUCR199	75	1	76.6
D11	LAN5_amplified	TUCR199	75	1	76.4
D12	LAN5_amplified	TUCR199	75	1	76
B11	LAN5_not amplified	TUCR199	75	1	76.2
B12	LAN5_not amplified	TUCR199	75	1	73
C5	GI-ME-N_amplified	TUCR2	75	1	74.8
C6	GI-ME-N_amplified	TUCR2	75	1	74.7
A5	GI-ME-N_not amplified	TUCR2	75	1	75.1
A6	GI-ME-N_not amplified	TUCR2	75	1	74.9
D5	LAN5_amplified	TUCR2	75	1	74.8
D6	LAN5_amplified	TUCR2	75	1	74.7
B5	LAN5_not amplified	TUCR2	75	1	74.9
B6	LAN5_not amplified	TUCR2	75	1	74.7
G3	GI-ME-N_amplified	TUCR20	80.7	1	80.4
G4	GI-ME-N_amplified	TUCR20	80.7	1	80.5
E3	GI-ME-N_not amplified	TUCR20	80.7	1	80.1
E4	GI-ME-N_not amplified	TUCR20	80.7	1	80.1
F4	LAN5_amplified	TUCR20	80.7	0	
F3	LAN5_amplified	TUCR20	80.7	1	80.1
H4	LAN5_not amplified	TUCR20	80.7	0	
H3	LAN5_not amplified	TUCR20	80.7	1	80.5
G2	GI-ME-N_amplified	TUCR200	84.4	0	
G1	GI-ME-N_amplified	TUCR200	84.4	1	83.3
E1	GI-ME-N_not amplified	TUCR200	84.4	0	
E2	GI-ME-N_not amplified	TUCR200	84.4	0	
F1	LAN5_amplified	TUCR200	84.4	0	
F2	LAN5_amplified	TUCR200	84.4	0	
H1	LAN5_not amplified	TUCR200	84.4	0	
H2	LAN5_not amplified	TUCR200	84.4	0	
G3	GI-ME-N_amplified	TUCR201	79.3	0	
G4	GI-ME-N_amplified	TUCR201	79.3	0	
E3	GI-ME-N_not amplified	TUCR201	79.3	0	
E4	GI-ME-N_not amplified	TUCR201	79.3	0	
F3	LAN5_amplified	TUCR201	79.3	1	80.8
F4	LAN5_amplified	TUCR201	79.3	1	78
H3	LAN5_not amplified	TUCR201	79.3	1	78.9
H4	LAN5_not amplified	TUCR201	79.3	2	77.5, 82.6

G5	GI-ME-N_amplified	TUCR202	80.2	1	80.6
G6	GI-ME-N_amplified	TUCR202	80.2	1	80.4
E5	GI-ME-N_not amplified	TUCR202	80.2	1	80.4
E6	GI-ME-N_not amplified	TUCR202	80.2	1	80.3
H5	LAN5_amplified	TUCR202	80.2	1	80.8
H6	LAN5_amplified	TUCR202	80.2	1	80.6
F5	LAN5_not amplified	TUCR202	80.2	1	80.3
F6	LAN5_not amplified	TUCR202	80.2	1	80.3
G7	GI-ME-N_amplified	TUCR203	81.7	1	82.7
G8	GI-ME-N_amplified	TUCR203	81.7	1	82.8
E7	GI-ME-N_not amplified	TUCR203	81.7	1	82.6
E8	GI-ME-N_not amplified	TUCR203	81.7	1	82.5
H7	LAN5_amplified	TUCR203	81.7	1	82.9
H8	LAN5_amplified	TUCR203	81.7	1	83
F8	LAN5_not amplified	TUCR203	81.7	0	
F7	LAN5_not amplified	TUCR203	81.7	1	82.5
G10	GI-ME-N_amplified	TUCR204	81.2	0	
G9	GI-ME-N_amplified	TUCR204	81.2	0	
E10	GI-ME-N_not amplified	TUCR204	81.2	0	
E9	GI-ME-N_not amplified	TUCR204	81.2	0	
H10	LAN5_amplified	TUCR204	81.2	1	74.6
H9	LAN5_amplified	TUCR204	81.2	1	79.3
F10	LAN5_not amplified	TUCR204	81.2	0	
F9	LAN5_not amplified	TUCR204	81.2	0	
G11	GI-ME-N_amplified	TUCR205	81.9	1	80.1
G12	GI-ME-N_amplified	TUCR205	81.9	2	77.1, 80.1
E11	GI-ME-N_not amplified	TUCR205	81.9	0	
E12	GI-ME-N_not amplified	TUCR205	81.9	0	
H12	LAN5_amplified	TUCR205	81.9	0	
H11	LAN5_amplified	TUCR205	81.9	1	80.4
F11	LAN5_not amplified	TUCR205	81.9	0	
F12	LAN5_not amplified	TUCR205	81.9	0	
C2	GI-ME-N_amplified	TUCR206	78.8	0	
C1	GI-ME-N_amplified	TUCR206	78.8	1	78.6
A1	GI-ME-N_not amplified	TUCR206	78.8	0	
A2	GI-ME-N_not amplified	TUCR206	78.8	0	
D2	LAN5_amplified	TUCR206	78.8	0	
D1	LAN5_amplified	TUCR206	78.8	1	78.7
B1	LAN5_not amplified	TUCR206	78.8	0	
B2	LAN5_not amplified	TUCR206	78.8	0	
C3	GI-ME-N_amplified	TUCR207	81.3	1	79.8
C4	GI-ME-N_amplified	TUCR207	81.3	1	79.9
A3	GI-ME-N_not amplified	TUCR207	81.3	1	80.2
A4	GI-ME-N_not amplified	TUCR207	81.3	1	80.3
D4	LAN5_amplified	TUCR207	81.3	0	
D3	LAN5_amplified	TUCR207	81.3	1	79.8
B3	LAN5_not amplified	TUCR207	81.3	1	80.1
B4	LAN5_not amplified	TUCR207	81.3	1	80.1
C5	GI-ME-N_amplified	TUCR208	78.1	1	76.8
C6	GI-ME-N_amplified	TUCR208	78.1	1	76.7
A5	GI-ME-N_not amplified	TUCR208	78.1	1	77.2
A6	GI-ME-N_not amplified	TUCR208	78.1	1	77
D5	LAN5_amplified	TUCR208	78.1	1	76.8
D6	LAN5_amplified	TUCR208	78.1	1	76.1
B5	LAN5_not amplified	TUCR208	78.1	1	77
B6	LAN5_not amplified	TUCR208	78.1	1	76.9
C7	GI-ME-N_amplified	TUCR209	77.8	1	76.6
C8	GI-ME-N_amplified	TUCR209	77.8	1	76.7
A7	GI-ME-N_not amplified	TUCR209	77.8	1	77
A8	GI-ME-N_not amplified	TUCR209	77.8	1	77
D7	LAN5_amplified	TUCR209	77.8	1	76.6
D8	LAN5_amplified	TUCR209	77.8	1	76.6
B7	LAN5_not amplified	TUCR209	77.8	1	76.8
B8	LAN5_not amplified	TUCR209	77.8	1	76.9
G5	GI-ME-N_amplified	TUCR21	78.9	1	78

G6	GI-ME-N_amplified	TUCR21	78.9	1	77.8
E5	GI-ME-N_not amplified	TUCR21	78.9	1	77.9
E6	GI-ME-N_not amplified	TUCR21	78.9	1	77.7
H5	LAN5_amplified	TUCR21	78.9	1	78
H6	LAN5_amplified	TUCR21	78.9	1	77.8
F5	LAN5_not amplified	TUCR21	78.9	0	
F6	LAN5_not amplified	TUCR21	78.9	1	74.8
C10	GI-ME-N_amplified	TUCR210	75.7	0	
C9	GI-ME-N_amplified	TUCR210	75.7	1	75.5
A10	GI-ME-N_not amplified	TUCR210	75.7	1	76.1
A9	GI-ME-N_not amplified	TUCR210	75.7	1	76.3
D10	LAN5_amplified	TUCR210	75.7	1	75.6
D9	LAN5_amplified	TUCR210	75.7	1	75.8
B10	LAN5_not amplified	TUCR210	75.7	1	75.9
B9	LAN5_not amplified	TUCR210	75.7	1	76.1
C11	GI-ME-N_amplified	TUCR211	77.2	1	76.4
C12	GI-ME-N_amplified	TUCR211	77.2	1	76.6
A11	GI-ME-N_not amplified	TUCR211	77.2	1	76.8
A12	GI-ME-N_not amplified	TUCR211	77.2	1	76.9
D12	LAN5_amplified	TUCR211	77.2	0	
D11	LAN5_amplified	TUCR211	77.2	1	76.5
B12	LAN5_not amplified	TUCR211	77.2	0	
B11	LAN5_not amplified	TUCR211	77.2	1	76.6
G1	GI-ME-N_amplified	TUCR212	83.2	1	83.3
G2	GI-ME-N_amplified	TUCR212	83.2	1	83.2
E1	GI-ME-N_not amplified	TUCR212	83.2	1	83.1
E2	GI-ME-N_not amplified	TUCR212	83.2	1	82.8
F2	LAN5_amplified	TUCR212	83.2	0	
F1	LAN5_amplified	TUCR212	83.2	1	79.3
H1	LAN5_not amplified	TUCR212	83.2	0	
H2	LAN5_not amplified	TUCR212	83.2	0	
G4	GI-ME-N_amplified	TUCR213	80.2	0	
G3	GI-ME-N_amplified	TUCR213	80.2	1	81.2
E3	GI-ME-N_not amplified	TUCR213	80.2	0	
E4	GI-ME-N_not amplified	TUCR213	80.2	1	81
F4	LAN5_amplified	TUCR213	80.2	0	
F3	LAN5_amplified	TUCR213	80.2	1	81
H3	LAN5_not amplified	TUCR213	80.2	0	
H4	LAN5_not amplified	TUCR213	80.2	0	
G5	GI-ME-N_amplified	TUCR214	77.1	0	
G6	GI-ME-N_amplified	TUCR214	77.1	0	
E5	GI-ME-N_not amplified	TUCR214	77.1	0	
E6	GI-ME-N_not amplified	TUCR214	77.1	0	
H5	LAN5_amplified	TUCR214	77.1	0	
H6	LAN5_amplified	TUCR214	77.1	0	
F5	LAN5_not amplified	TUCR214	77.1	0	
F6	LAN5_not amplified	TUCR214	77.1	1	76.7
G7	GI-ME-N_amplified	TUCR215	82.5	0	
G8	GI-ME-N_amplified	TUCR215	82.5	2	80.2, 82.1
E7	GI-ME-N_not amplified	TUCR215	82.5	0	
E8	GI-ME-N_not amplified	TUCR215	82.5	0	
H7	LAN5_amplified	TUCR215	82.5	1	82.4
H8	LAN5_amplified	TUCR215	82.5	1	82.8
F7	LAN5_not amplified	TUCR215	82.5	1	82.1
F8	LAN5_not amplified	TUCR215	82.5	1	82
G10	GI-ME-N_amplified	TUCR216	82.1	1	81.5
G9	GI-ME-N_amplified	TUCR216	82.1	1	81.6
E10	GI-ME-N_not amplified	TUCR216	82.1	1	81.4
E9	GI-ME-N_not amplified	TUCR216	82.1	1	81.6
H10	LAN5_amplified	TUCR216	82.1	1	81.7
H9	LAN5_amplified	TUCR216	82.1	1	81.8
F10	LAN5_not amplified	TUCR216	82.1	1	81.4
F9	LAN5_not amplified	TUCR216	82.1	1	81.6
G11	GI-ME-N_amplified	TUCR217	81.8	1	80.5
G12	GI-ME-N_amplified	TUCR217	81.8	1	80.5

E11	GI-ME-N_not amplified	TUCR217	81.8	1	80.3
E12	GI-ME-N_not amplified	TUCR217	81.8	1	80.6
H11	LAN5_amplified	TUCR217	81.8	1	80.7
H12	LAN5_amplified	TUCR217	81.8	1	80.8
F11	LAN5_not amplified	TUCR217	81.8	1	80.2
F12	LAN5_not amplified	TUCR217	81.8	1	80.6
C1	GI-ME-N_amplified	TUCR218	77.9	1	77
C2	GI-ME-N_amplified	TUCR218	77.9	1	76.9
A1	GI-ME-N_not amplified	TUCR218	77.9	1	77.3
A2	GI-ME-N_not amplified	TUCR218	77.9	1	77.2
D1	LAN5_amplified	TUCR218	77.9	1	77.2
D2	LAN5_amplified	TUCR218	77.9	1	76.9
B1	LAN5_not amplified	TUCR218	77.9	1	77.2
B2	LAN5_not amplified	TUCR218	77.9	1	77
C3	GI-ME-N_amplified	TUCR219	79	1	78.3
C4	GI-ME-N_amplified	TUCR219	79	1	78.5
A3	GI-ME-N_not amplified	TUCR219	79	1	78.8
A4	GI-ME-N_not amplified	TUCR219	79	1	78.9
D3	LAN5_amplified	TUCR219	79	1	78.4
D4	LAN5_amplified	TUCR219	79	1	78.4
B3	LAN5_not amplified	TUCR219	79	1	78.5
B4	LAN5_not amplified	TUCR219	79	1	78.7
G7	GI-ME-N_amplified	TUCR22	73.3	1	74
G8	GI-ME-N_amplified	TUCR22	73.3	1	74
E7	GI-ME-N_not amplified	TUCR22	73.3	1	74
E8	GI-ME-N_not amplified	TUCR22	73.3	1	74
H7	LAN5_amplified	TUCR22	73.3	1	74.1
H8	LAN5_amplified	TUCR22	73.3	1	74.1
F7	LAN5_not amplified	TUCR22	73.3	1	73.9
F8	LAN5_not amplified	TUCR22	73.3	1	74
C5	GI-ME-N_amplified	TUCR220	75.7	1	77.2
C6	GI-ME-N_amplified	TUCR220	75.7	1	77.1
A5	GI-ME-N_not amplified	TUCR220	75.7	1	77.5
A6	GI-ME-N_not amplified	TUCR220	75.7	1	77.5
D5	LAN5_amplified	TUCR220	75.7	1	77.1
D6	LAN5_amplified	TUCR220	75.7	1	77.2
B5	LAN5_not amplified	TUCR220	75.7	1	77.4
B6	LAN5_not amplified	TUCR220	75.7	1	77.2
C7	GI-ME-N_amplified	TUCR221	80.3	1	79
C8	GI-ME-N_amplified	TUCR221	80.3	1	79.1
A7	GI-ME-N_not amplified	TUCR221	80.3	1	79.4
A8	GI-ME-N_not amplified	TUCR221	80.3	1	79.5
D7	LAN5_amplified	TUCR221	80.3	1	79
D8	LAN5_amplified	TUCR221	80.3	1	79
B7	LAN5_not amplified	TUCR221	80.3	1	79.2
B8	LAN5_not amplified	TUCR221	80.3	1	79.3
C10	GI-ME-N_amplified	TUCR222	74.4	0	
C9	GI-ME-N_amplified	TUCR222	74.4	1	74.1
A10	GI-ME-N_not amplified	TUCR222	74.4	0	
A9	GI-ME-N_not amplified	TUCR222	74.4	1	74.5
D10	LAN5_amplified	TUCR222	74.4	0	
D9	LAN5_amplified	TUCR222	74.4	0	
B9	LAN5_not amplified	TUCR222	74.4	0	
B10	LAN5_not amplified	TUCR222	74.4	1	74.2
C11	GI-ME-N_amplified	TUCR223	80.2	1	78.8
C12	GI-ME-N_amplified	TUCR223	80.2	1	78.6
A12	GI-ME-N_not amplified	TUCR223	80.2	0	
A11	GI-ME-N_not amplified	TUCR223	80.2	1	79.1
D12	LAN5_amplified	TUCR223	80.2	0	
D11	LAN5_amplified	TUCR223	80.2	1	79.1
B11	LAN5_not amplified	TUCR223	80.2	0	
B12	LAN5_not amplified	TUCR223	80.2	0	
G1	GI-ME-N_amplified	TUCR224	81.3	1	81.4
G2	GI-ME-N_amplified	TUCR224	81.3	1	81.2
E1	GI-ME-N_not amplified	TUCR224	81.3	0	

E2	GI-ME-N_not amplified	TUCR224	81.3	0	
F1	LAN5_amplified	TUCR224	81.3	0	
F2	LAN5_amplified	TUCR224	81.3	1	81
H1	LAN5_not amplified	TUCR224	81.3	1	76.5
H2	LAN5_not amplified	TUCR224	81.3	1	77.8
G4	GI-ME-N_amplified	TUCR225	78.5	0	
G3	GI-ME-N_amplified	TUCR225	78.5	1	77.1
E3	GI-ME-N_not amplified	TUCR225	78.5	1	77.3
E4	GI-ME-N_not amplified	TUCR225	78.5	1	80.6
F3	LAN5_amplified	TUCR225	78.5	0	
F4	LAN5_amplified	TUCR225	78.5	1	74.8
H3	LAN5_not amplified	TUCR225	78.5	0	
H4	LAN5_not amplified	TUCR225	78.5	0	
G5	GI-ME-N_amplified	TUCR226	75.3	1	75.4
G6	GI-ME-N_amplified	TUCR226	75.3	1	75.3
E6	GI-ME-N_not amplified	TUCR226	75.3	0	
E5	GI-ME-N_not amplified	TUCR226	75.3	1	74.2
H6	LAN5_amplified	TUCR226	75.3	0	
H5	LAN5_amplified	TUCR226	75.3	1	75.3
F5	LAN5_not amplified	TUCR226	75.3	0	
F6	LAN5_not amplified	TUCR226	75.3	0	
G7	GI-ME-N_amplified	TUCR227	77.4	1	76.5
G8	GI-ME-N_amplified	TUCR227	77.4	1	76.5
E7	GI-ME-N_not amplified	TUCR227	77.4	0	
E8	GI-ME-N_not amplified	TUCR227	77.4	0	
H8	LAN5_amplified	TUCR227	77.4	0	
H7	LAN5_amplified	TUCR227	77.4	1	76.4
F7	LAN5_not amplified	TUCR227	77.4	0	
F8	LAN5_not amplified	TUCR227	77.4	0	
G10	GI-ME-N_amplified	TUCR228	79	1	78.2
G9	GI-ME-N_amplified	TUCR228	79	1	78.4
E10	GI-ME-N_not amplified	TUCR228	79	0	
E9	GI-ME-N_not amplified	TUCR228	79	1	78.6
H10	LAN5_amplified	TUCR228	79	0	
H9	LAN5_amplified	TUCR228	79	0	
F10	LAN5_not amplified	TUCR228	79	0	
F9	LAN5_not amplified	TUCR228	79	1	78.3
G12	GI-ME-N_amplified	TUCR229	77.9	0	
G11	GI-ME-N_amplified	TUCR229	77.9	1	79.2
E11	GI-ME-N_not amplified	TUCR229	77.9	0	
E12	GI-ME-N_not amplified	TUCR229	77.9	0	
H11	LAN5_amplified	TUCR229	77.9	0	
H12	LAN5_amplified	TUCR229	77.9	0	
F11	LAN5_not amplified	TUCR229	77.9	0	
F12	LAN5_not amplified	TUCR229	77.9	0	
G10	GI-ME-N_amplified	TUCR23	82.1	1	80.5
G9	GI-ME-N_amplified	TUCR23	82.1	1	80.7
E10	GI-ME-N_not amplified	TUCR23	82.1	1	80.5
E9	GI-ME-N_not amplified	TUCR23	82.1	1	80.6
H10	LAN5_amplified	TUCR23	82.1	1	80.7
H9	LAN5_amplified	TUCR23	82.1	1	80.9
F10	LAN5_not amplified	TUCR23	82.1	1	80.4
F9	LAN5_not amplified	TUCR23	82.1	1	80.6
C1	GI-ME-N_amplified	TUCR230	79.4	0	
C2	GI-ME-N_amplified	TUCR230	79.4	1	70.3
A1	GI-ME-N_not amplified	TUCR230	79.4	0	
A2	GI-ME-N_not amplified	TUCR230	79.4	1	78.9
D1	LAN5_amplified	TUCR230	79.4	1	70.7
D2	LAN5_amplified	TUCR230	79.4	1	70.5
B2	LAN5_not amplified	TUCR230	79.4	0	
B1	LAN5_not amplified	TUCR230	79.4	1	72.8
C3	GI-ME-N_amplified	TUCR231	76.6	1	74.8
C4	GI-ME-N_amplified	TUCR231	76.6	1	74.9
A3	GI-ME-N_not amplified	TUCR231	76.6	0	
A4	GI-ME-N_not amplified	TUCR231	76.6	0	

D3	LAN5_amplified	TUCR231	76.6	0	
D4	LAN5_amplified	TUCR231	76.6	0	
B3	LAN5_not amplified	TUCR231	76.6	0	
B4	LAN5_not amplified	TUCR231	76.6	0	
C5	GI-ME-N_amplified	TUCR232	71.2	0	
C6	GI-ME-N_amplified	TUCR232	71.2	0	
A5	GI-ME-N_not amplified	TUCR232	71.2	0	
A6	GI-ME-N_not amplified	TUCR232	71.2	0	
D5	LAN5_amplified	TUCR232	71.2	1	72.9
D6	LAN5_amplified	TUCR232	71.2	1	72.8
B5	LAN5_not amplified	TUCR232	71.2	1	72.9
B6	LAN5_not amplified	TUCR232	71.2	1	73.9
C7	GI-ME-N_amplified	TUCR233	74.8	1	75.6
C8	GI-ME-N_amplified	TUCR233	74.8	1	75.7
A7	GI-ME-N_not amplified	TUCR233	74.8	1	76.1
A8	GI-ME-N_not amplified	TUCR233	74.8	1	76.1
D7	LAN5_amplified	TUCR233	74.8	1	75.6
D8	LAN5_amplified	TUCR233	74.8	1	75.7
B7	LAN5_not amplified	TUCR233	74.8	1	75.8
B8	LAN5_not amplified	TUCR233	74.8	1	76
C10	GI-ME-N_amplified	TUCR234	78.9	1	77.7
C9	GI-ME-N_amplified	TUCR234	78.9	1	77.9
A10	GI-ME-N_not amplified	TUCR234	78.9	1	78.2
A9	GI-ME-N_not amplified	TUCR234	78.9	1	78.2
D9	LAN5_amplified	TUCR234	78.9	0	
D10	LAN5_amplified	TUCR234	78.9	1	76.9
B10	LAN5_not amplified	TUCR234	78.9	0	
B9	LAN5_not amplified	TUCR234	78.9	0	
C11	GI-ME-N_amplified	TUCR235	78.3	1	77
C12	GI-ME-N_amplified	TUCR235	78.3	1	77.2
A11	GI-ME-N_not amplified	TUCR235	78.3	1	77.6
A12	GI-ME-N_not amplified	TUCR235	78.3	1	73.7
D11	LAN5_amplified	TUCR235	78.3	1	77.5
D12	LAN5_amplified	TUCR235	78.3	1	77.6
B11	LAN5_not amplified	TUCR235	78.3	0	
B12	LAN5_not amplified	TUCR235	78.3	0	
G1	GI-ME-N_amplified	TUCR236	74.3	1	76.1
G2	GI-ME-N_amplified	TUCR236	74.3	1	75.9
E2	GI-ME-N_not amplified	TUCR236	74.3	0	
E1	GI-ME-N_not amplified	TUCR236	74.3	1	75.5
F1	LAN5_amplified	TUCR236	74.3	1	75.8
F2	LAN5_amplified	TUCR236	74.3	1	75.7
H1	LAN5_not amplified	TUCR236	74.3	1	77.4
H2	LAN5_not amplified	TUCR236	74.3	1	77.1
G3	GI-ME-N_amplified	TUCR237	81.9	1	80.7
G4	GI-ME-N_amplified	TUCR237	81.9	1	80.7
E3	GI-ME-N_not amplified	TUCR237	81.9	1	80.4
E4	GI-ME-N_not amplified	TUCR237	81.9	1	80.4
F4	LAN5_amplified	TUCR237	81.9	0	
F3	LAN5_amplified	TUCR237	81.9	1	75.7
H3	LAN5_not amplified	TUCR237	81.9	0	
H4	LAN5_not amplified	TUCR237	81.9	0	
G6	GI-ME-N_amplified	TUCR238	82.2	0	
G5	GI-ME-N_amplified	TUCR238	82.2	1	79.9
E6	GI-ME-N_not amplified	TUCR238	82.2	0	
E5	GI-ME-N_not amplified	TUCR238	82.2	1	80.6
H5	LAN5_amplified	TUCR238	82.2	0	
H6	LAN5_amplified	TUCR238	82.2	0	
F5	LAN5_not amplified	TUCR238	82.2	1	80.7
F6	LAN5_not amplified	TUCR238	82.2	1	80.6
G7	GI-ME-N_amplified	TUCR239	80.2	1	78.7
G8	GI-ME-N_amplified	TUCR239	80.2	1	78.8
E7	GI-ME-N_not amplified	TUCR239	80.2	1	78.7
E8	GI-ME-N_not amplified	TUCR239	80.2	1	78.7
H7	LAN5_amplified	TUCR239	80.2	1	79

H8	LAN5_amplified	TUCR239	80.2	1	79
F7	LAN5_not amplified	TUCR239	80.2	1	78.7
F8	LAN5_not amplified	TUCR239	80.2	1	78.7
G11	GI-ME-N_amplified	TUCR24	75.4	1	80.6
G12	GI-ME-N_amplified	TUCR24	75.4	1	80.9
E11	GI-ME-N_not amplified	TUCR24	75.4	1	80.5
E12	GI-ME-N_not amplified	TUCR24	75.4	1	80.7
H11	LAN5_amplified	TUCR24	75.4	1	80.9
H12	LAN5_amplified	TUCR24	75.4	1	81
F11	LAN5_not amplified	TUCR24	75.4	1	80.5
F12	LAN5_not amplified	TUCR24	75.4	1	80.7
G10	GI-ME-N_amplified	TUCR240	81.4	1	81.1
G9	GI-ME-N_amplified	TUCR240	81.4	1	81.2
E10	GI-ME-N_not amplified	TUCR240	81.4	0	
E9	GI-ME-N_not amplified	TUCR240	81.4	0	
H10	LAN5_amplified	TUCR240	81.4	1	81
H9	LAN5_amplified	TUCR240	81.4	1	81.5
F10	LAN5_not amplified	TUCR240	81.4	0	
F9	LAN5_not amplified	TUCR240	81.4	1	81.3
G11	GI-ME-N_amplified	TUCR241	77.1	1	76.4
G12	GI-ME-N_amplified	TUCR241	77.1	1	76.6
E11	GI-ME-N_not amplified	TUCR241	77.1	1	76.5
E12	GI-ME-N_not amplified	TUCR241	77.1	1	76.6
H11	LAN5_amplified	TUCR241	77.1	0	
H12	LAN5_amplified	TUCR241	77.1	1	81.9
F11	LAN5_not amplified	TUCR241	77.1	1	76.5
F12	LAN5_not amplified	TUCR241	77.1	1	76.7
C1	GI-ME-N_amplified	TUCR242	79.1	2	77.5, 79.8
C2	GI-ME-N_amplified	TUCR242	79.1	2	74.9, 79.2
A2	GI-ME-N_not amplified	TUCR242	79.1	0	
A1	GI-ME-N_not amplified	TUCR242	79.1	2	72.9, 79.2
D1	LAN5_amplified	TUCR242	79.1	1	72.8
D2	LAN5_amplified	TUCR242	79.1	2	72.8, 75.5
B1	LAN5_not amplified	TUCR242	79.1	1	73.2
B2	LAN5_not amplified	TUCR242	79.1	1	72.8
C3	GI-ME-N_amplified	TUCR243	76.1	1	75.3
C4	GI-ME-N_amplified	TUCR243	76.1	1	75.5
A3	GI-ME-N_not amplified	TUCR243	76.1	1	75.7
A4	GI-ME-N_not amplified	TUCR243	76.1	1	75.8
D3	LAN5_amplified	TUCR243	76.1	1	75.5
D4	LAN5_amplified	TUCR243	76.1	1	75.5
B3	LAN5_not amplified	TUCR243	76.1	1	75.5
B4	LAN5_not amplified	TUCR243	76.1	1	75.6
C6	GI-ME-N_amplified	TUCR244	77.7	0	
C5	GI-ME-N_amplified	TUCR244	77.7	1	77.9
A5	GI-ME-N_not amplified	TUCR244	77.7	0	
A6	GI-ME-N_not amplified	TUCR244	77.7	0	
D5	LAN5_amplified	TUCR244	77.7	1	77.8
D6	LAN5_amplified	TUCR244	77.7	1	77.4
B5	LAN5_not amplified	TUCR244	77.7	1	79.4
B6	LAN5_not amplified	TUCR244	77.7	1	79.3
C7	GI-ME-N_amplified	TUCR245	81.7	1	79.9
C8	GI-ME-N_amplified	TUCR245	81.7	1	80
A7	GI-ME-N_not amplified	TUCR245	81.7	1	80.2
A8	GI-ME-N_not amplified	TUCR245	81.7	1	80.4
D7	LAN5_amplified	TUCR245	81.7	1	79.9
D8	LAN5_amplified	TUCR245	81.7	1	79.9
B7	LAN5_not amplified	TUCR245	81.7	1	80
B8	LAN5_not amplified	TUCR245	81.7	1	80.2
C10	GI-ME-N_amplified	TUCR246	81	1	80.6
C9	GI-ME-N_amplified	TUCR246	81	1	80.8
A10	GI-ME-N_not amplified	TUCR246	81	1	81
A9	GI-ME-N_not amplified	TUCR246	81	1	81.2
D10	LAN5_amplified	TUCR246	81	1	80.6
D9	LAN5_amplified	TUCR246	81	1	80.7

B10	LAN5_not amplified	TUCR246	81	1	80.8
B9	LAN5_not amplified	TUCR246	81	1	80.9
C11	GI-ME-N_amplified	TUCR247	79.5	1	77.8
C12	GI-ME-N_amplified	TUCR247	79.5	1	78
A12	GI-ME-N_not amplified	TUCR247	79.5	0	
A11	GI-ME-N_not amplified	TUCR247	79.5	1	78.1
D11	LAN5_amplified	TUCR247	79.5	1	77.8
D12	LAN5_amplified	TUCR247	79.5	1	77.9
B11	LAN5_not amplified	TUCR247	79.5	0	
B12	LAN5_not amplified	TUCR247	79.5	0	
G1	GI-ME-N_amplified	TUCR248	80.5	1	80.2
G2	GI-ME-N_amplified	TUCR248	80.5	1	80
E1	GI-ME-N_not amplified	TUCR248	80.5	0	
E2	GI-ME-N_not amplified	TUCR248	80.5	0	
F2	LAN5_amplified	TUCR248	80.5	0	
F1	LAN5_amplified	TUCR248	80.5	1	80.1
H2	LAN5_not amplified	TUCR248	80.5	0	
H1	LAN5_not amplified	TUCR248	80.5	1	80.3
G3	GI-ME-N_amplified	TUCR249	79.3	0	
G4	GI-ME-N_amplified	TUCR249	79.3	0	
E3	GI-ME-N_not amplified	TUCR249	79.3	0	
E4	GI-ME-N_not amplified	TUCR249	79.3	0	
F3	LAN5_amplified	TUCR249	79.3	1	79
F4	LAN5_amplified	TUCR249	79.3	1	79.1
H3	LAN5_not amplified	TUCR249	79.3	1	79.1
H4	LAN5_not amplified	TUCR249	79.3	1	79.4
C2	GI-ME-N_amplified	TUCR25	79	0	
C1	GI-ME-N_amplified	TUCR25	79	1	77.7
A2	GI-ME-N_not amplified	TUCR25	79	0	
A1	GI-ME-N_not amplified	TUCR25	79	1	78.3
D1	LAN5_amplified	TUCR25	79	1	78.3
D2	LAN5_amplified	TUCR25	79	1	78.2
B2	LAN5_not amplified	TUCR25	79	0	
B1	LAN5_not amplified	TUCR25	79	1	78
G5	GI-ME-N_amplified	TUCR250	76.5	1	76.3
G6	GI-ME-N_amplified	TUCR250	76.5	1	76.2
E5	GI-ME-N_not amplified	TUCR250	76.5	1	76
E6	GI-ME-N_not amplified	TUCR250	76.5	1	75.8
H5	LAN5_amplified	TUCR250	76.5	1	76.3
H6	LAN5_amplified	TUCR250	76.5	1	76.2
F5	LAN5_not amplified	TUCR250	76.5	1	76.3
F6	LAN5_not amplified	TUCR250	76.5	1	75.9
G7	GI-ME-N_amplified	TUCR251	83.5	1	82.7
G8	GI-ME-N_amplified	TUCR251	83.5	1	82.8
E7	GI-ME-N_not amplified	TUCR251	83.5	1	82.5
E8	GI-ME-N_not amplified	TUCR251	83.5	1	82.4
H7	LAN5_amplified	TUCR251	83.5	1	83
H8	LAN5_amplified	TUCR251	83.5	1	83
F7	LAN5_not amplified	TUCR251	83.5	1	82.4
F8	LAN5_not amplified	TUCR251	83.5	1	82.6
G10	GI-ME-N_amplified	TUCR252	79.8	1	78.5
G9	GI-ME-N_amplified	TUCR252	79.8	1	78.6
E10	GI-ME-N_not amplified	TUCR252	79.8	1	78.4
E9	GI-ME-N_not amplified	TUCR252	79.8	1	78.5
H10	LAN5_amplified	TUCR252	79.8	1	78.7
H9	LAN5_amplified	TUCR252	79.8	1	78.8
F10	LAN5_not amplified	TUCR252	79.8	1	78.4
F9	LAN5_not amplified	TUCR252	79.8	1	78.5
G11	GI-ME-N_amplified	TUCR253	78.5	1	78.6
G12	GI-ME-N_amplified	TUCR253	78.5	1	78.7
E11	GI-ME-N_not amplified	TUCR253	78.5	1	78.3
E12	GI-ME-N_not amplified	TUCR253	78.5	1	78.5
H11	LAN5_amplified	TUCR253	78.5	1	78.2
H12	LAN5_amplified	TUCR253	78.5	1	78.4
F11	LAN5_not amplified	TUCR253	78.5	1	78.2

F12	LAN5_not amplified	TUCR253	78.5	1	78.5
C1	GI-ME-N_amplified	TUCR254	74.4	1	75.1
C2	GI-ME-N_amplified	TUCR254	74.4	1	75.3
A1	GI-ME-N_not amplified	TUCR254	74.4	1	75.3
A2	GI-ME-N_not amplified	TUCR254	74.4	1	75.2
D2	LAN5_amplified	TUCR254	74.4	0	
D1	LAN5_amplified	TUCR254	74.4	1	75.7
B1	LAN5_not amplified	TUCR254	74.4	1	75.1
B2	LAN5_not amplified	TUCR254	74.4	1	74.9
C3	GI-ME-N_amplified	TUCR255	74.6	1	74.8
C4	GI-ME-N_amplified	TUCR255	74.6	1	75
A3	GI-ME-N_not amplified	TUCR255	74.6	1	75.1
A4	GI-ME-N_not amplified	TUCR255	74.6	1	75.3
D3	LAN5_amplified	TUCR255	74.6	1	75
D4	LAN5_amplified	TUCR255	74.1	1	75
B3	LAN5_not amplified	TUCR255	74.6	1	75
B4	LAN5_not amplified	TUCR255	74.6	1	75.1
C5	GI-ME-N_amplified	TUCR256	74.1	1	74
C6	GI-ME-N_amplified	TUCR256	74.1	1	73.9
A5	GI-ME-N_not amplified	TUCR256	74.1	1	74.4
A6	GI-ME-N_not amplified	TUCR256	74.1	1	74.3
D5	LAN5_amplified	TUCR256	74.1	1	73.9
D6	LAN5_amplified	TUCR256	74.1	1	73.9
B5	LAN5_not amplified	TUCR256	74.1	1	74.1
B6	LAN5_not amplified	TUCR256	74.1	1	74
C7	GI-ME-N_amplified	TUCR257	79.1	1	77.7
C8	GI-ME-N_amplified	TUCR257	79.1	1	77.8
A7	GI-ME-N_not amplified	TUCR257	79.1	1	78.1
A8	GI-ME-N_not amplified	TUCR257	79.1	1	78.2
D7	LAN5_amplified	TUCR257	79.1	1	77.7
D8	LAN5_amplified	TUCR257	79.1	1	77.7
B7	LAN5_not amplified	TUCR257	79.1	1	77.9
B8	LAN5_not amplified	TUCR257	79.1	1	78
C10	GI-ME-N_amplified	TUCR258	79.8	1	78.7
C9	GI-ME-N_amplified	TUCR258	79.8	1	78.5
A10	GI-ME-N_not amplified	TUCR258	79.8	1	78.7
A9	GI-ME-N_not amplified	TUCR258	79.8	1	78.9
D10	LAN5_amplified	TUCR258	79.8	1	78.9
D9	LAN5_amplified	TUCR258	79.8	1	79.1
B10	LAN5_not amplified	TUCR258	79.8	1	79.2
B9	LAN5_not amplified	TUCR258	79.8	1	79.4
C12	GI-ME-N_amplified	TUCR259	76	1	76.1
C11	GI-ME-N_amplified	TUCR259	76	1	76.4
A11	GI-ME-N_not amplified	TUCR259	76	1	76.2
A12	GI-ME-N_not amplified	TUCR259	76	1	74.5
D12	LAN5_amplified	TUCR259	76	0	
D11	LAN5_amplified	TUCR259	76	1	76.3
B11	LAN5_not amplified	TUCR259	76	1	75.8
B12	LAN5_not amplified	TUCR259	76	1	75.9
C3	GI-ME-N_amplified	TUCR26	77.8	1	76.3
C4	GI-ME-N_amplified	TUCR26	77.8	1	76.4
A3	GI-ME-N_not amplified	TUCR26	77.8	1	76.6
A4	GI-ME-N_not amplified	TUCR26	77.8	1	76.7
D3	LAN5_amplified	TUCR26	77.8	1	76.5
D4	LAN5_amplified	TUCR26	77.8	1	76.5
B3	LAN5_not amplified	TUCR26	77.8	1	76.4
B4	LAN5_not amplified	TUCR26	77.8	1	76.6
G1	GI-ME-N_amplified	TUCR260	81.9	1	81.4
G2	GI-ME-N_amplified	TUCR260	81.9	1	81.3
E1	GI-ME-N_not amplified	TUCR260	81.9	1	81.1
E2	GI-ME-N_not amplified	TUCR260	81.9	1	80.9
F1	LAN5_amplified	TUCR260	81.9	1	81.3
F2	LAN5_amplified	TUCR260	81.9	1	81.1
H1	LAN5_not amplified	TUCR260	81.9	1	81.6
H2	LAN5_not amplified	TUCR260	81.9	1	75

G3	GI-ME-N_amplified	TUCR261	79	1	78.9
G4	GI-ME-N_amplified	TUCR261	79	1	78.9
E3	GI-ME-N_not amplified	TUCR261	79	1	78.6
E4	GI-ME-N_not amplified	TUCR261	79	1	78.6
F3	LAN5_amplified	TUCR261	79	1	78.6
F4	LAN5_amplified	TUCR261	79	1	78.6
H3	LAN5_not amplified	TUCR261	79	0	
H4	LAN5_not amplified	TUCR261	79	0	
G5	GI-ME-N_amplified	TUCR262	79.1	1	79
G6	GI-ME-N_amplified	TUCR262	79.1	1	78.8
E5	GI-ME-N_not amplified	TUCR262	79.1	1	74.3
E6	GI-ME-N_not amplified	TUCR262	79.1	1	74.8
H5	LAN5_amplified	TUCR262	79.1	1	79.1
H6	LAN5_amplified	TUCR262	79.1	1	78.9
F5	LAN5_not amplified	TUCR262	79.1	1	78
F6	LAN5_not amplified	TUCR262	79.1	1	77.8
G7	GI-ME-N_amplified	TUCR263	84.8	1	84.3
G8	GI-ME-N_amplified	TUCR263	84.8	1	84.4
E7	GI-ME-N_not amplified	TUCR263	84.8	1	84.2
E8	GI-ME-N_not amplified	TUCR263	84.8	1	84.3
H8	LAN5_amplified	TUCR263	84.8	0	
H7	LAN5_amplified	TUCR263	84.8	1	84.6
F7	LAN5_not amplified	TUCR263	84.8	1	84.2
F8	LAN5_not amplified	TUCR263	84.8	1	84.3
G10	GI-ME-N_amplified	TUCR264	76.8	1	75.9
G9	GI-ME-N_amplified	TUCR264	76.8	1	76.1
E10	GI-ME-N_not amplified	TUCR264	76.8	1	75.9
E9	GI-ME-N_not amplified	TUCR264	76.8	1	76.1
H10	LAN5_amplified	TUCR264	76.8	1	75.5
H9	LAN5_amplified	TUCR264	76.8	1	76.2
F10	LAN5_not amplified	TUCR264	76.8	1	75.9
F9	LAN5_not amplified	TUCR264	76.8	1	76.1
G11	GI-ME-N_amplified	TUCR265	83.5	1	82.2
G12	GI-ME-N_amplified	TUCR265	83.5	1	82.4
E11	GI-ME-N_not amplified	TUCR265	83.5	1	82.1
E12	GI-ME-N_not amplified	TUCR265	83.5	1	82.4
H11	LAN5_amplified	TUCR265	83.5	1	82.5
H12	LAN5_amplified	TUCR265	83.5	1	82.6
F11	LAN5_not amplified	TUCR265	83.5	1	82.1
F12	LAN5_not amplified	TUCR265	83.5	1	82.4
C1	GI-ME-N_amplified	TUCR266	81.3	1	80.2
C2	GI-ME-N_amplified	TUCR266	81.3	1	80.1
A1	GI-ME-N_not amplified	TUCR266	81.3	1	80.6
A2	GI-ME-N_not amplified	TUCR266	81.3	1	80.4
D2	LAN5_amplified	TUCR266	81.3	0	
D1	LAN5_amplified	TUCR266	81.3	1	80.3
B1	LAN5_not amplified	TUCR266	81.3	1	80.1
B2	LAN5_not amplified	TUCR266	81.3	1	80.1
C3	GI-ME-N_amplified	TUCR267	76.2	1	76.6
C4	GI-ME-N_amplified	TUCR267	76.2	1	76.8
A3	GI-ME-N_not amplified	TUCR267	76.2	1	76.9
A4	GI-ME-N_not amplified	TUCR267	76.2	1	77
D3	LAN5_amplified	TUCR267	76.2	1	76.7
D4	LAN5_amplified	TUCR267	76.2	1	76.8
B3	LAN5_not amplified	TUCR267	76.2	1	76.7
B4	LAN5_not amplified	TUCR267	76.2	1	76.9
C5	GI-ME-N_amplified	TUCR268	79.1	1	78.7
C6	GI-ME-N_amplified	TUCR268	79.1	1	78.6
A5	GI-ME-N_not amplified	TUCR268	79.1	1	79
A6	GI-ME-N_not amplified	TUCR268	79.1	1	78.9
D5	LAN5_amplified	TUCR268	79.1	1	78.7
D6	LAN5_amplified	TUCR268	79.1	1	78.6
B5	LAN5_not amplified	TUCR268	79.1	1	78.9
B6	LAN5_not amplified	TUCR268	79.1	1	78.7
C7	GI-ME-N_amplified	TUCR269	81.7	1	80.8

C8	GI-ME-N_amplified	TUCR269	81.7	1	80.9
A7	GI-ME-N_not amplified	TUCR269	81.7	1	81.1
A8	GI-ME-N_not amplified	TUCR269	81.7	1	81.3
D7	LAN5_amplified	TUCR269	81.7	1	80.8
D8	LAN5_amplified	TUCR269	81.7	1	80.8
B7	LAN5_not amplified	TUCR269	81.7	1	80.9
B8	LAN5_not amplified	TUCR269	81.7	1	81
C5	GI-ME-N_amplified	TUCR27	80.5	1	79.8
C6	GI-ME-N_amplified	TUCR27	80.5	1	79.7
A5	GI-ME-N_not amplified	TUCR27	80.5	1	80.2
A6	GI-ME-N_not amplified	TUCR27	80.5	1	80.1
D5	LAN5_amplified	TUCR27	80.5	1	79.8
D6	LAN5_amplified	TUCR27	80.5	1	79.7
B5	LAN5_not amplified	TUCR27	80.5	1	80
B6	LAN5_not amplified	TUCR27	80.5	1	79.9
C10	GI-ME-N_amplified	TUCR270	85.2	1	83.7
C9	GI-ME-N_amplified	TUCR270	85.2	1	83.9
A10	GI-ME-N_not amplified	TUCR270	85.2	1	84
A9	GI-ME-N_not amplified	TUCR270	85.2	1	84.4
D10	LAN5_amplified	TUCR270	85.2	1	83.7
D9	LAN5_amplified	TUCR270	85.2	1	83.8
B10	LAN5_not amplified	TUCR270	85.2	1	83.9
B9	LAN5_not amplified	TUCR270	85.2	1	84.1
C11	GI-ME-N_amplified	TUCR271	82	1	80.9
C12	GI-ME-N_amplified	TUCR271	82	1	81.2
A11	GI-ME-N_not amplified	TUCR271	82	1	81.4
A12	GI-ME-N_not amplified	TUCR271	82	1	81.5
D11	LAN5_amplified	TUCR271	82	1	81
D12	LAN5_amplified	TUCR271	82	1	81.1
B11	LAN5_not amplified	TUCR271	82	1	81.2
B12	LAN5_not amplified	TUCR271	82	1	81.3
G1	GI-ME-N_amplified	TUCR272	73.6	1	74.6
G2	GI-ME-N_amplified	TUCR272	73.6	1	74.5
E1	GI-ME-N_not amplified	TUCR272	73.6	1	74.5
E2	GI-ME-N_not amplified	TUCR272	73.6	1	74.3
F1	LAN5_amplified	TUCR272	73.6	1	74.5
F2	LAN5_amplified	TUCR272	73.6	1	74.5
H1	LAN5_not amplified	TUCR272	73.6	1	74.9
H2	LAN5_not amplified	TUCR272	73.6	1	74.7
G3	GI-ME-N_amplified	TUCR273	78.6	1	78.9
G4	GI-ME-N_amplified	TUCR273	78.6	1	79
E3	GI-ME-N_not amplified	TUCR273	78.6	1	78.7
E4	GI-ME-N_not amplified	TUCR273	78.6	1	78.7
F3	LAN5_amplified	TUCR273	78.6	1	78.7
F4	LAN5_amplified	TUCR273	78.6	1	78.8
H3	LAN5_not amplified	TUCR273	78.6	1	79.1
H4	LAN5_not amplified	TUCR273	78.6	1	79.2
G5	GI-ME-N_amplified	TUCR274	79.1	1	78.4
G6	GI-ME-N_amplified	TUCR274	79.1	1	78.3
E5	GI-ME-N_not amplified	TUCR274	79.1	1	78.3
E6	GI-ME-N_not amplified	TUCR274	79.1	1	78.2
H5	LAN5_amplified	TUCR274	79.1	1	78.6
H6	LAN5_amplified	TUCR274	79.1	1	78.4
F5	LAN5_not amplified	TUCR274	79.1	1	78.4
F6	LAN5_not amplified	TUCR274	79.1	1	78.2
G7	GI-ME-N_amplified	TUCR275	77.2	1	76.1
G8	GI-ME-N_amplified	TUCR275	77.2	1	76.2
E7	GI-ME-N_not amplified	TUCR275	77.2	1	76.1
E8	GI-ME-N_not amplified	TUCR275	77.2	1	76.1
H7	LAN5_amplified	TUCR275	77.2	1	76.3
H8	LAN5_amplified	TUCR275	77.2	1	76.3
F7	LAN5_not amplified	TUCR275	77.2	1	76.1
F8	LAN5_not amplified	TUCR275	77.2	1	76.1
G10	GI-ME-N_amplified	TUCR276	81.8	1	82.1
G9	GI-ME-N_amplified	TUCR276	81.8	1	82.3

E10	GI-ME-N_not amplified	TUCR276	81.8	1	82
E9	GI-ME-N_not amplified	TUCR276	81.8	1	82.1
H10	LAN5_amplified	TUCR276	81.8	1	82.4
H9	LAN5_amplified	TUCR276	81.8	1	82.5
F10	LAN5_not amplified	TUCR276	81.8	1	82
F9	LAN5_not amplified	TUCR276	81.8	1	82.2
G11	GI-ME-N_amplified	TUCR277	80.9	1	80.5
G12	GI-ME-N_amplified	TUCR277	80.9	1	80.8
E11	GI-ME-N_not amplified	TUCR277	80.9	1	80.5
E12	GI-ME-N_not amplified	TUCR277	80.9	1	80.7
H11	LAN5_amplified	TUCR277	80.9	1	80.7
H12	LAN5_amplified	TUCR277	80.9	1	80.9
F11	LAN5_not amplified	TUCR277	80.9	1	80.4
F12	LAN5_not amplified	TUCR277	80.9	1	80.7
C1	GI-ME-N_amplified	TUCR278	80	1	79.1
C2	GI-ME-N_amplified	TUCR278	80	1	78.8
A1	GI-ME-N_not amplified	TUCR278	80	1	79.4
A2	GI-ME-N_not amplified	TUCR278	80	1	78.9
D1	LAN5_amplified	TUCR278	80	1	79.2
D2	LAN5_amplified	TUCR278	80	1	79
B1	LAN5_not amplified	TUCR278	80	1	79.2
B2	LAN5_not amplified	TUCR278	80	1	78.8
C3	GI-ME-N_amplified	TUCR279	78.6	1	77.7
C4	GI-ME-N_amplified	TUCR279	78.6	1	77.4
A3	GI-ME-N_not amplified	TUCR279	78.6	1	78
A4	GI-ME-N_not amplified	TUCR279	78.6	1	78.1
D3	LAN5_amplified	TUCR279	78.6	1	77.8
D4	LAN5_amplified	TUCR279	78.6	1	77.9
B3	LAN5_not amplified	TUCR279	78.6	1	77.8
B4	LAN5_not amplified	TUCR279	78.6	1	77.9
C7	GI-ME-N_amplified	TUCR28	75.1	1	75.6
C8	GI-ME-N_amplified	TUCR28	75.1	1	75.7
A7	GI-ME-N_not amplified	TUCR28	75.1	1	76
A8	GI-ME-N_not amplified	TUCR28	75.1	1	76.1
D7	LAN5_amplified	TUCR28	75.1	1	75.6
D8	LAN5_amplified	TUCR28	75.1	1	75.6
B7	LAN5_not amplified	TUCR28	75.1	1	75.7
B8	LAN5_not amplified	TUCR28	75.1	1	75.8
C5	GI-ME-N_amplified	TUCR280	86.9	1	87.3
C6	GI-ME-N_amplified	TUCR280	86.9	1	87.2
A5	GI-ME-N_not amplified	TUCR280	86.9	1	87.6
A6	GI-ME-N_not amplified	TUCR280	86.9	1	87.5
D5	LAN5_amplified	TUCR280	86.9	1	87.4
D6	LAN5_amplified	TUCR280	86.9	1	87
B5	LAN5_not amplified	TUCR280	86.9	1	87.4
B6	LAN5_not amplified	TUCR280	86.9	1	87.3
C7	GI-ME-N_amplified	TUCR281	78.8	1	78.8
C8	GI-ME-N_amplified	TUCR281	78.8	1	79
A7	GI-ME-N_not amplified	TUCR281	78.8	1	79.3
A8	GI-ME-N_not amplified	TUCR281	78.8	1	79
D7	LAN5_amplified	TUCR281	78.8	1	78.8
D8	LAN5_amplified	TUCR281	78.8	1	78.9
B7	LAN5_not amplified	TUCR281	78.8	1	78.8
B8	LAN5_not amplified	TUCR281	78.8	1	79.2
C10	GI-ME-N_amplified	TUCR282	80.9	1	79.7
C9	GI-ME-N_amplified	TUCR282	80.9	1	79.2
A10	GI-ME-N_not amplified	TUCR282	80.9	1	79.9
A9	GI-ME-N_not amplified	TUCR282	80.9	1	79.8
D10	LAN5_amplified	TUCR282	80.9	1	79.3
D9	LAN5_amplified	TUCR282	80.9	1	79.2
B10	LAN5_not amplified	TUCR282	80.9	1	79.7
B9	LAN5_not amplified	TUCR282	80.9	1	79.4
C12	GI-ME-N_amplified	TUCR283	81.4	0	
C11	GI-ME-N_amplified	TUCR283	81.4	1	81.1
A11	GI-ME-N_not amplified	TUCR283	81.4	1	81.4

A12	GI-ME-N_not amplified	TUCR283	81.4	1	81.6
D11	LAN5_amplified	TUCR283	81.4	0	
D12	LAN5_amplified	TUCR283	81.4	1	81.2
B11	LAN5_not amplified	TUCR283	81.4	1	81.2
B12	LAN5_not amplified	TUCR283	81.4	1	81.3
G1	GI-ME-N_amplified	TUCR284	77	1	77.3
G2	GI-ME-N_amplified	TUCR284	77	1	77
E1	GI-ME-N_not amplified	TUCR284	77	1	71.3
E2	GI-ME-N_not amplified	TUCR284	77	1	71.1
F1	LAN5_amplified	TUCR284	77	1	77.3
F2	LAN5_amplified	TUCR284	77	1	77.3
H1	LAN5_not amplified	TUCR284	77	1	77.3
H2	LAN5_not amplified	TUCR284	77	1	77.6
G3	GI-ME-N_amplified	TUCR285	84.3	1	83.6
G4	GI-ME-N_amplified	TUCR285	84.3	1	83.7
E3	GI-ME-N_not amplified	TUCR285	84.3	1	83.3
E4	GI-ME-N_not amplified	TUCR285	84.3	1	83.4
F3	LAN5_amplified	TUCR285	84.3	1	83.5
F4	LAN5_amplified	TUCR285	84.3	1	83.5
H3	LAN5_not amplified	TUCR285	84.3	1	83.5
H4	LAN5_not amplified	TUCR285	84.3	1	83.9
G5	GI-ME-N_amplified	TUCR286	73.8	1	74.7
G6	GI-ME-N_amplified	TUCR286	73.8	1	74.6
E5	GI-ME-N_not amplified	TUCR286	73.8	1	74.3
E6	GI-ME-N_not amplified	TUCR286	73.8	1	74.3
H5	LAN5_amplified	TUCR286	73.8	1	75
H6	LAN5_amplified	TUCR286	73.8	1	74.7
F5	LAN5_not amplified	TUCR286	73.8	1	76.5
F6	LAN5_not amplified	TUCR286	73.8	1	74.2
G7	GI-ME-N_amplified	TUCR287	80.6	1	79.4
G8	GI-ME-N_amplified	TUCR287	80.6	1	79.5
E7	GI-ME-N_not amplified	TUCR287	80.6	1	79.4
E8	GI-ME-N_not amplified	TUCR287	80.6	1	79.4
H7	LAN5_amplified	TUCR287	80.6	1	79.3
H8	LAN5_amplified	TUCR287	80.6	1	79.7
F7	LAN5_not amplified	TUCR287	80.6	1	79.4
F8	LAN5_not amplified	TUCR287	80.6	1	79.4
G10	GI-ME-N_amplified	TUCR288	79.1	1	77.4
G9	GI-ME-N_amplified	TUCR288	79.1	1	76.4
E10	GI-ME-N_not amplified	TUCR288	79.1	0	
E9	GI-ME-N_not amplified	TUCR288	79.1	1	77.4
H10	LAN5_amplified	TUCR288	79.1	1	77.4
H9	LAN5_amplified	TUCR288	79.1	1	77.6
F10	LAN5_not amplified	TUCR288	79.1	1	77.4
F9	LAN5_not amplified	TUCR288	79.1	1	77.4
G11	GI-ME-N_amplified	TUCR289	77.1	1	75.7
G12	GI-ME-N_amplified	TUCR289	77.1	1	75.9
E11	GI-ME-N_not amplified	TUCR289	77.1	1	75.8
E12	GI-ME-N_not amplified	TUCR289	77.1	1	76
H11	LAN5_amplified	TUCR289	77.1	1	76
H12	LAN5_amplified	TUCR289	77.1	1	76.1
F11	LAN5_not amplified	TUCR289	77.1	1	75.8
F12	LAN5_not amplified	TUCR289	77.1	1	75.9
C10	GI-ME-N_amplified	TUCR29	76	1	75.7
C9	GI-ME-N_amplified	TUCR29	76	1	75.9
A10	GI-ME-N_not amplified	TUCR29	76	1	76.1
A9	GI-ME-N_not amplified	TUCR29	76	1	76.3
D10	LAN5_amplified	TUCR29	76	1	75.6
D9	LAN5_amplified	TUCR29	76	1	75.8
B10	LAN5_not amplified	TUCR29	76	1	76
B9	LAN5_not amplified	TUCR29	76	1	76.1
C1	GI-ME-N_amplified	TUCR290	80	1	80
C2	GI-ME-N_amplified	TUCR290	80	1	79.8
A1	GI-ME-N_not amplified	TUCR290	80	1	80.2
A2	GI-ME-N_not amplified	TUCR290	80	1	80.1

D1	LAN5_amplified	TUCR290	80	1	80.1
D2	LAN5_amplified	TUCR290	80	1	79.9
B1	LAN5_not amplified	TUCR290	80	1	80.1
B2	LAN5_not amplified	TUCR290	80	1	79.9
C3	GI-ME-N_amplified	TUCR291	75.9	1	76.2
C4	GI-ME-N_amplified	TUCR291	75.9	1	76.1
A3	GI-ME-N_not amplified	TUCR291	75.9	1	76.5
A4	GI-ME-N_not amplified	TUCR291	75.9	1	76.6
D3	LAN5_amplified	TUCR291	75.9	1	76.2
D4	LAN5_amplified	TUCR291	75.9	1	76.3
B4	LAN5_not amplified	TUCR291	75.9	0	
B3	LAN5_not amplified	TUCR291	75.9	1	76.1
C5	GI-ME-N_amplified	TUCR292	81.5	1	80.1
C6	GI-ME-N_amplified	TUCR292	81.5	1	80.2
A5	GI-ME-N_not amplified	TUCR292	81.5	1	80.5
A6	GI-ME-N_not amplified	TUCR292	81.5	1	80.4
D5	LAN5_amplified	TUCR292	81.5	1	80.1
D6	LAN5_amplified	TUCR292	81.5	1	80.1
B5	LAN5_not amplified	TUCR292	81.5	1	80.1
B6	LAN5_not amplified	TUCR292	81.5	1	76.3
C8	GI-ME-N_amplified	TUCR293	79.2	0	
C7	GI-ME-N_amplified	TUCR293	79.2	1	79.9
A7	GI-ME-N_not amplified	TUCR293	79.2	1	75.3
A8	GI-ME-N_not amplified	TUCR293	79.2	1	79.2
D7	LAN5_amplified	TUCR293	79.2	1	79.9
D8	LAN5_amplified	TUCR293	79.2	1	79.8
B7	LAN5_not amplified	TUCR293	79.2	1	78.9
B8	LAN5_not amplified	TUCR293	79.2	1	79
C10	GI-ME-N_amplified	TUCR294	81.7	1	78.6
C9	GI-ME-N_amplified	TUCR294	81.7	1	80
A10	GI-ME-N_not amplified	TUCR294	81.7	0	
A9	GI-ME-N_not amplified	TUCR294	81.7	1	80.3
D10	LAN5_amplified	TUCR294	81.7	1	79.8
D9	LAN5_amplified	TUCR294	81.7	1	79.8
B9	LAN5_not amplified	TUCR294	81.7	0	
B10	LAN5_not amplified	TUCR294	81.7	1	79.9
C11	GI-ME-N_amplified	TUCR295	79.5	0	
C12	GI-ME-N_amplified	TUCR295	79.5	1	74.7
A11	GI-ME-N_not amplified	TUCR295	79.5	1	79.1
A12	GI-ME-N_not amplified	TUCR295	79.5	1	79.5
D11	LAN5_amplified	TUCR295	79.5	1	79.3
D12	LAN5_amplified	TUCR295	79.5	3	
B11	LAN5_not amplified	TUCR295	79.5	1	79.1
B12	LAN5_not amplified	TUCR295	79.5	1	79.3
G1	GI-ME-N_amplified	TUCR296	84	1	83.1
G2	GI-ME-N_amplified	TUCR296	84	1	82.7
E1	GI-ME-N_not amplified	TUCR296	84	1	82.7
E2	GI-ME-N_not amplified	TUCR296	84	1	82.4
F1	LAN5_amplified	TUCR296	84	1	82.8
F2	LAN5_amplified	TUCR296	84	1	82.5
H1	LAN5_not amplified	TUCR296	84	1	83.2
H2	LAN5_not amplified	TUCR296	84	1	83.1
G4	GI-ME-N_amplified	TUCR297	80.7	0	
G3	GI-ME-N_amplified	TUCR297	80.7	1	81
E3	GI-ME-N_not amplified	TUCR297	80.7	1	80.3
E4	GI-ME-N_not amplified	TUCR297	80.7	1	80.4
F3	LAN5_amplified	TUCR297	80.7	1	80.5
F4	LAN5_amplified	TUCR297	80.7	1	80.5
H3	LAN5_not amplified	TUCR297	80.7	1	77.9
H4	LAN5_not amplified	TUCR297	80.7	1	80.9
G5	GI-ME-N_amplified	TUCR298	77.5	1	74.2
G6	GI-ME-N_amplified	TUCR298	77.5	1	74.2
E5	GI-ME-N_not amplified	TUCR298	77.5	1	74.6
E6	GI-ME-N_not amplified	TUCR298	77.5	1	74.4
H5	LAN5_amplified	TUCR298	77.5	1	74.9

H6	LAN5_amplified	TUCR298	77.5	1	74.4
F5	LAN5_not amplified	TUCR298	77.5	1	74.7
F6	LAN5_not amplified	TUCR298	77.5	1	74.5
G7	GI-ME-N_amplified	TUCR299	88.9	1	80.6
G8	GI-ME-N_amplified	TUCR299	88.9	1	79.4
E7	GI-ME-N_not amplified	TUCR299	88.9	0	
E8	GI-ME-N_not amplified	TUCR299	88.9	0	
H8	LAN5_amplified	TUCR299	88.9	1	79.8
H7	LAN5_amplified	TUCR299	88.9	2	76.8, 79.7
F7	LAN5_not amplified	TUCR299	88.9	0	
F8	LAN5_not amplified	TUCR299	88.9	0	
C7	GI-ME-N_amplified	TUCR3	81.9	1	80.6
C8	GI-ME-N_amplified	TUCR3	81.9	1	80.6
A7	GI-ME-N_not amplified	TUCR3	81.9	1	81
A8	GI-ME-N_not amplified	TUCR3	81.9	1	81.1
D7	LAN5_amplified	TUCR3	81.9	1	80.5
D8	LAN5_amplified	TUCR3	81.9	1	80.5
B7	LAN5_not amplified	TUCR3	81.9	1	80.7
B8	LAN5_not amplified	TUCR3	81.9	1	80.9
C12	GI-ME-N_amplified	TUCR30	75.4	10	
C11	GI-ME-N_amplified	TUCR30	75.4	12	
A11	GI-ME-N_not amplified	TUCR30	75.4	0	
A12	GI-ME-N_not amplified	TUCR30	75.4	0	
D11	LAN5_amplified	TUCR30	75.4	10	
D12	LAN5_amplified	TUCR30	75.4	11	
B11	LAN5_not amplified	TUCR30	75.4	0	
B12	LAN5_not amplified	TUCR30	75.4	4	
G10	GI-ME-N_amplified	TUCR300	75.3	1	72.1
G9	GI-ME-N_amplified	TUCR300	75.3	1	72
E10	GI-ME-N_not amplified	TUCR300	75.3	2	71.6, 74.9
E9	GI-ME-N_not amplified	TUCR300	75.3	2	71.5, 74.8
H10	LAN5_amplified	TUCR300	75.3	1	72.4
H9	LAN5_amplified	TUCR300	75.3	1	72.1
F10	LAN5_not amplified	TUCR300	75.3	1	71.6
F9	LAN5_not amplified	TUCR300	75.3	1	71.5
G11	GI-ME-N_amplified	TUCR301	80.5	1	80.8
G12	GI-ME-N_amplified	TUCR301	80.5	1	81
E11	GI-ME-N_not amplified	TUCR301	80.5	1	81
E12	GI-ME-N_not amplified	TUCR301	80.5	2	74.9, 80.7
H11	LAN5_amplified	TUCR301	80.5	1	80.1
H12	LAN5_amplified	TUCR301	80.5	1	80.7
F12	LAN5_not amplified	TUCR301	80.5	0	
F11	LAN5_not amplified	TUCR301	80.5	1	80.7
C1	GI-ME-N_amplified	TUCR302	77.9	1	75.8
C2	GI-ME-N_amplified	TUCR302	77.9	1	75.5
A2	GI-ME-N_not amplified	TUCR302	77.9	0	
A1	GI-ME-N_not amplified	TUCR302	77.9	1	75
D2	LAN5_amplified	TUCR302	77.9	0	
D1	LAN5_amplified	TUCR302	77.9	1	76
B2	LAN5_not amplified	TUCR302	77.9	0	
B1	LAN5_not amplified	TUCR302	77.9	1	75.7
C3	GI-ME-N_amplified	TUCR303	84.9	1	83.3
C4	GI-ME-N_amplified	TUCR303	84.9	1	83.8
A4	GI-ME-N_not amplified	TUCR303	84.9	0	
A3	GI-ME-N_not amplified	TUCR303	84.9	1	78.2
D3	LAN5_amplified	TUCR303	84.9	1	83.9
D4	LAN5_amplified	TUCR303	84.9	1	83.5
B3	LAN5_not amplified	TUCR303	84.9	1	83.5
B4	LAN5_not amplified	TUCR303	84.9	1	83.7
C5	GI-ME-N_amplified	TUCR304	79	1	78
C6	GI-ME-N_amplified	TUCR304	79	1	73.4
A5	GI-ME-N_not amplified	TUCR304	79	1	78.8
A6	GI-ME-N_not amplified	TUCR304	79	1	74.4
D5	LAN5_amplified	TUCR304	79	1	78.7
D6	LAN5_amplified	TUCR304	79	1	78.4

B5	LAN5_not amplified	TUCR304	79	1	78.4
B6	LAN5_not amplified	TUCR304	79	1	78.5
C7	GI-ME-N_amplified	TUCR305	82.9	1	81.4
C8	GI-ME-N_amplified	TUCR305	82.9	1	81.6
A7	GI-ME-N_not amplified	TUCR305	82.9	1	81.9
A8	GI-ME-N_not amplified	TUCR305	82.9	1	81.8
D7	LAN5_amplified	TUCR305	82.9	1	81.5
D8	LAN5_amplified	TUCR305	82.9	1	81.5
B7	LAN5_not amplified	TUCR305	82.9	1	81.6
B8	LAN5_not amplified	TUCR305	82.9	1	81.7
C10	GI-ME-N_amplified	TUCR306	81.8	1	80.4
C9	GI-ME-N_amplified	TUCR306	81.8	1	80.6
A10	GI-ME-N_not amplified	TUCR306	81.8	1	80.8
A9	GI-ME-N_not amplified	TUCR306	81.8	1	80.9
D10	LAN5_amplified	TUCR306	81.8	1	80.4
D9	LAN5_amplified	TUCR306	81.8	1	80.5
B10	LAN5_not amplified	TUCR306	81.8	1	80.6
B9	LAN5_not amplified	TUCR306	81.8	1	80.7
C11	GI-ME-N_amplified	TUCR307	75.3	1	74.9
C12	GI-ME-N_amplified	TUCR307	75.3	1	75
A11	GI-ME-N_not amplified	TUCR307	75.3	1	75.3
A12	GI-ME-N_not amplified	TUCR307	75.3	1	75.3
D11	LAN5_amplified	TUCR307	75.3	1	74.9
D12	LAN5_amplified	TUCR307	75.3	1	75.1
B11	LAN5_not amplified	TUCR307	75.3	1	74.9
B12	LAN5_not amplified	TUCR307	75.3	1	75.2
G1	GI-ME-N_amplified	TUCR308	79.5	1	80.5
G2	GI-ME-N_amplified	TUCR308	79.5	1	80.2
E1	GI-ME-N_not amplified	TUCR308	79.5	1	79.6
E2	GI-ME-N_not amplified	TUCR308	79.5	1	79.5
F1	LAN5_amplified	TUCR308	79.5	1	79.8
F2	LAN5_amplified	TUCR308	79.5	1	79.5
H1	LAN5_not amplified	TUCR308	79.5	1	80.7
H2	LAN5_not amplified	TUCR308	79.5	1	80.5
G3	GI-ME-N_amplified	TUCR309	81.9	1	82.9
G4	GI-ME-N_amplified	TUCR309	81.9	1	82.9
E3	GI-ME-N_not amplified	TUCR309	81.9	1	82.6
E4	GI-ME-N_not amplified	TUCR309	81.9	1	82.7
F3	LAN5_amplified	TUCR309	81.9	1	82.8
F4	LAN5_amplified	TUCR309	81.9	1	82.8
H3	LAN5_not amplified	TUCR309	81.9	1	83
H4	LAN5_not amplified	TUCR309	81.9	1	83.2
G1	GI-ME-N_amplified	TUCR31	73.2	1	75.7
G2	GI-ME-N_amplified	TUCR31	73.2	1	76.4
E1	GI-ME-N_not amplified	TUCR31	73.2	0	
E2	GI-ME-N_not amplified	TUCR31	73.2	1	74.2
F2	LAN5_amplified	TUCR31	73.2	0	
F1	LAN5_amplified	TUCR31	73.2	1	76.5
H2	LAN5_not amplified	TUCR31	73.2	0	
H1	LAN5_not amplified	TUCR31	73.2	1	75.6
G6	GI-ME-N_amplified	TUCR310	78.1	0	
G5	GI-ME-N_amplified	TUCR310	78.1	1	76.6
E5	GI-ME-N_not amplified	TUCR310	78.1	1	77
E6	GI-ME-N_not amplified	TUCR310	78.1	1	76.9
H5	LAN5_amplified	TUCR310	78.1	1	76.8
H6	LAN5_amplified	TUCR310	78.1	1	77.1
F5	LAN5_not amplified	TUCR310	78.1	1	77
F6	LAN5_not amplified	TUCR310	78.1	1	76.8
G7	GI-ME-N_amplified	TUCR311	76	1	75.7
G8	GI-ME-N_amplified	TUCR311	76	1	75.8
E7	GI-ME-N_not amplified	TUCR311	76	1	75.8
E8	GI-ME-N_not amplified	TUCR311	76	1	75.7
H7	LAN5_amplified	TUCR311	76	1	76
H8	LAN5_amplified	TUCR311	76	1	76
F7	LAN5_not amplified	TUCR311	76	1	75.8

F8	LAN5_not amplified	TUCR311	76	1	76
G10	GI-ME-N_amplified	TUCR312	77.8	1	78.3
G9	GI-ME-N_amplified	TUCR312	77.8	1	78.3
E10	GI-ME-N_not amplified	TUCR312	77.8	1	78.1
E9	GI-ME-N_not amplified	TUCR312	77.8	1	78.2
H10	LAN5_amplified	TUCR312	77.8	0	
H9	LAN5_amplified	TUCR312	77.8	1	78.5
F10	LAN5_not amplified	TUCR312	77.8	0	
F9	LAN5_not amplified	TUCR312	77.8	1	78.2
G11	GI-ME-N_amplified	TUCR313	76	1	76.3
G12	GI-ME-N_amplified	TUCR313	76	1	76.5
E11	GI-ME-N_not amplified	TUCR313	76	1	76.3
E12	GI-ME-N_not amplified	TUCR313	76	1	76.3
H12	LAN5_amplified	TUCR313	76	0	
H11	LAN5_amplified	TUCR313	76	1	76.5
F12	LAN5_not amplified	TUCR313	76	0	
F11	LAN5_not amplified	TUCR313	76	1	76.2
C2	GI-ME-N_amplified	TUCR314	81.1	0	
C1	GI-ME-N_amplified	TUCR314	81.1	1	79.6
A1	GI-ME-N_not amplified	TUCR314	81.1	0	
A2	GI-ME-N_not amplified	TUCR314	81.1	0	
D2	LAN5_amplified	TUCR314	81.1	0	
D1	LAN5_amplified	TUCR314	81.1	1	77.3
B2	LAN5_not amplified	TUCR314	81.1	0	
B1	LAN5_not amplified	TUCR314	81.1	1	79.5
C4	GI-ME-N_amplified	TUCR315	79.1	0	
C3	GI-ME-N_amplified	TUCR315	79.1	1	79.2
A3	GI-ME-N_not amplified	TUCR315	79.1	0	
A4	GI-ME-N_not amplified	TUCR315	79.1	0	
D3	LAN5_amplified	TUCR315	79.1	1	79.2
D4	LAN5_amplified	TUCR315	79.1	1	79.1
B4	LAN5_not amplified	TUCR315	79.1	1	79.1
B3	LAN5_not amplified	TUCR315	79.1	1	79.1
C6	GI-ME-N_amplified	TUCR316	76.7	0	
C5	GI-ME-N_amplified	TUCR316	76.7	1	78.6
A6	GI-ME-N_not amplified	TUCR316	76.7	0	
A5	GI-ME-N_not amplified	TUCR316	76.7	1	77.3
D6	LAN5_amplified	TUCR316	76.7	0	
D5	LAN5_amplified	TUCR316	76.7	1	77
B5	LAN5_not amplified	TUCR316	76.7	1	77.2
B6	LAN5_not amplified	TUCR316	76.7	1	77
C7	GI-ME-N_amplified	TUCR317	73.5	1	73.8
C8	GI-ME-N_amplified	TUCR317	73.5	1	73.9
A7	GI-ME-N_not amplified	TUCR317	73.5	1	74.3
A8	GI-ME-N_not amplified	TUCR317	73.5	1	74.3
D7	LAN5_amplified	TUCR317	73.5	1	73.7
D8	LAN5_amplified	TUCR317	73.5	1	73.8
B7	LAN5_not amplified	TUCR317	73.5	1	74
B8	LAN5_not amplified	TUCR317	73.5	1	74.1
C10	GI-ME-N_amplified	TUCR318	75.4	1	75.6
C9	GI-ME-N_amplified	TUCR318	75.4	1	75.7
A10	GI-ME-N_not amplified	TUCR318	75.4	1	75.9
A9	GI-ME-N_not amplified	TUCR318	75.4	1	76.1
D10	LAN5_amplified	TUCR318	75.4	1	74.9
D9	LAN5_amplified	TUCR318	75.4	1	75.4
B10	LAN5_not amplified	TUCR318	75.4	1	75.2
B9	LAN5_not amplified	TUCR318	75.4	1	75.5
C11	GI-ME-N_amplified	TUCR319	76.5	1	76
C12	GI-ME-N_amplified	TUCR319	76.5	1	76.4
A11	GI-ME-N_not amplified	TUCR319	76.5	1	76.7
A12	GI-ME-N_not amplified	TUCR319	76.5	1	76.8
D11	LAN5_amplified	TUCR319	76.5	1	76.1
D12	LAN5_amplified	TUCR319	76.5	1	76.1
B12	LAN5_not amplified	TUCR319	76.5	0	
B11	LAN5_not amplified	TUCR319	76.5	1	76.4

G1	GI-ME-N_amplified	TUCR320	72.5	1	75.4
G2	GI-ME-N_amplified	TUCR320	72.5	1	75.2
E1	GI-ME-N_not amplified	TUCR320	72.5	1	75.1
E2	GI-ME-N_not amplified	TUCR320	72.5	1	75.2
F2	LAN5_amplified	TUCR320	72.5	0	
F1	LAN5_amplified	TUCR320	72.5	1	75
H1	LAN5_not amplified	TUCR320	72.5	1	75.4
H2	LAN5_not amplified	TUCR320	72.5	1	75.2
G3	GI-ME-N_amplified	TUCR321	78.4	1	78.6
G4	GI-ME-N_amplified	TUCR321	78.4	1	72.4
E4	GI-ME-N_not amplified	TUCR321	78.4	0	
E3	GI-ME-N_not amplified	TUCR321	78.4	1	78.1
F4	LAN5_amplified	TUCR321	78.4	0	
F3	LAN5_amplified	TUCR321	78.4	1	78.1
H3	LAN5_not amplified	TUCR321	78.4	1	78.4
H4	LAN5_not amplified	TUCR321	78.4	1	78.4
G5	GI-ME-N_amplified	TUCR322	78.4	1	77
G6	GI-ME-N_amplified	TUCR322	78.4	1	76.8
E6	GI-ME-N_not amplified	TUCR322	78.4	1	0
E5	GI-ME-N_not amplified	TUCR322	78.4	1	76.9
H5	LAN5_amplified	TUCR322	78.4	1	77.1
H6	LAN5_amplified	TUCR322	78.4	1	77
F5	LAN5_not amplified	TUCR322	78.4	1	77
F6	LAN5_not amplified	TUCR322	78.4	1	76.8
G7	GI-ME-N_amplified	TUCR323	78.5	1	77.4
G8	GI-ME-N_amplified	TUCR323	78.5	1	77.5
E7	GI-ME-N_not amplified	TUCR323	78.5	1	77.4
E8	GI-ME-N_not amplified	TUCR323	78.5	1	77.4
H7	LAN5_amplified	TUCR323	78.5	1	77.8
H8	LAN5_amplified	TUCR323	78.5	1	77.8
F7	LAN5_not amplified	TUCR323	78.5	1	77.4
F8	LAN5_not amplified	TUCR323	78.5	1	77.5
G10	GI-ME-N_amplified	TUCR324	84.9	1	83.3
G9	GI-ME-N_amplified	TUCR324	84.9	1	83.1
E10	GI-ME-N_not amplified	TUCR324	84.9	1	83.1
E9	GI-ME-N_not amplified	TUCR324	84.9	1	83
H10	LAN5_amplified	TUCR324	84.9	1	83.4
H9	LAN5_amplified	TUCR324	84.9	1	83.3
F10	LAN5_not amplified	TUCR324	84.9	0	
F9	LAN5_not amplified	TUCR324	84.9	1	83
G11	GI-ME-N_amplified	TUCR325	79.9	1	78.8
G12	GI-ME-N_amplified	TUCR325	79.9	1	79.2
E11	GI-ME-N_not amplified	TUCR325	79.9	1	78.8
E12	GI-ME-N_not amplified	TUCR325	79.9	1	79
H12	LAN5_amplified	TUCR325	79.9	0	
H11	LAN5_amplified	TUCR325	79.9	1	79.2
F12	LAN5_not amplified	TUCR325	79.9	0	
F11	LAN5_not amplified	TUCR325	79.9	1	78.8
C1	GI-ME-N_amplified	TUCR326	25.23	1	75.7
C2	GI-ME-N_amplified	TUCR326		1	76.4
A1	GI-ME-N_not amplified	TUCR326	27.23	1	76.3
A2	GI-ME-N_not amplified	TUCR326		1	76.7
D1	LAN5_amplified	TUCR326	33.54	1	76.7
D2	LAN5_amplified	TUCR326		1	76.5
B1	LAN5_not amplified	TUCR326	40	1	76.2
B2	LAN5_not amplified	TUCR326		1	76.4
C3	GI-ME-N_amplified	TUCR327	40	2	72.6, 78.4
C4	GI-ME-N_amplified	TUCR327	40	2	72.7, 78.4
A3	GI-ME-N_not amplified	TUCR327	40	1	73.8
A4	GI-ME-N_not amplified	TUCR327	40	1	73.5
D3	LAN5_amplified	TUCR327	28.41	1	78
D4	LAN5_amplified	TUCR327		2	72.7, 78.5
B3	LAN5_not amplified	TUCR327	34.22	1	73.3
B4	LAN5_not amplified	TUCR327		1	73.4
C5	GI-ME-N_amplified	TUCR328	40	0	

C6	GI-ME-N_amplified	TUCR328	40	1	79.7
A6	GI-ME-N_not amplified	TUCR328	40	0	
A5	GI-ME-N_not amplified	TUCR328	40	1	82
D5	LAN5_amplified	TUCR328	28.45	1	82.5
D6	LAN5_amplified	TUCR328	28.73	1	74.7
B5	LAN5_not amplified	TUCR328	34.31	0	
B6	LAN5_not amplified	TUCR328		0	
C7	GI-ME-N_amplified	TUCR329	31.97	1	78.1
C8	GI-ME-N_amplified	TUCR329	33.15	1	78.2
A7	GI-ME-N_not amplified	TUCR329	33.95	0	
A8	GI-ME-N_not amplified	TUCR329		1	78.5
D7	LAN5_amplified	TUCR329	30.72	1	78.2
D8	LAN5_amplified	TUCR329		1	78.1
B8	LAN5_not amplified	TUCR329	35.44	0	
B7	LAN5_not amplified	TUCR329	30.3	1	78.2
G3	GI-ME-N_amplified	TUCR33	84.4	1	83
G4	GI-ME-N_amplified	TUCR33	84.4	1	83.1
E3	GI-ME-N_not amplified	TUCR33	84.4	1	83.1
E4	GI-ME-N_not amplified	TUCR33	84.4	1	82.7
F3	LAN5_amplified	TUCR33	84.4	1	82.8
F4	LAN5_amplified	TUCR33	84.4	1	82.8
H3	LAN5_not amplified	TUCR33	84.4	1	83.2
H4	LAN5_not amplified	TUCR33	84.4	1	83.3
C10	GI-ME-N_amplified	TUCR330	30.56	1	81.8
C9	GI-ME-N_amplified	TUCR330	30.3	1	82
A10	GI-ME-N_not amplified	TUCR330	33.24	1	82.3
A9	GI-ME-N_not amplified	TUCR330	29.58	1	82.4
D10	LAN5_amplified	TUCR330	25.6	1	81.9
D9	LAN5_amplified	TUCR330	25.54	1	81.7
B10	LAN5_not amplified	TUCR330	29.57	1	82
B9	LAN5_not amplified	TUCR330	29.15	1	82.1
C11	GI-ME-N_amplified	TUCR331	24.77	1	77.9
C12	GI-ME-N_amplified	TUCR331	24.85	1	78.2
A11	GI-ME-N_not amplified	TUCR331	27.24	1	78.4
A12	GI-ME-N_not amplified	TUCR331	27.3	1	78.5
D11	LAN5_amplified	TUCR331	27.52	1	78
D12	LAN5_amplified	TUCR331		1	78.2
B11	LAN5_not amplified	TUCR331	32.39	1	78.1
B12	LAN5_not amplified	TUCR331		1	78.3
G1	GI-ME-N_amplified	TUCR332	25.56	1	76.8
G2	GI-ME-N_amplified	TUCR332	25.6	1	76.9
E1	GI-ME-N_not amplified	TUCR332	28.6	1	76.7
E2	GI-ME-N_not amplified	TUCR332	29.28	1	76.4
F2	LAN5_amplified	TUCR332	21.51	0	
F1	LAN5_amplified	TUCR332	21.35	1	76.6
H1	LAN5_not amplified	TUCR332	23.74	1	77
H2	LAN5_not amplified	TUCR332	23.9	1	76
G3	GI-ME-N_amplified	TUCR333	21.98	1	82.4
G4	GI-ME-N_amplified	TUCR333	22	1	82.5
E3	GI-ME-N_not amplified	TUCR333	23.57	1	82
E4	GI-ME-N_not amplified	TUCR333	23.63	1	82.1
F3	LAN5_amplified	TUCR333	32.36	1	82.1
F4	LAN5_amplified	TUCR333	33.38	1	82.2
H3	LAN5_not amplified	TUCR333	40	1	82.7
H4	LAN5_not amplified	TUCR333		1	82.7
G5	GI-ME-N_amplified	TUCR334	27.75	1	80.9
G6	GI-ME-N_amplified	TUCR334		1	80.4
E5	GI-ME-N_not amplified	TUCR334	29.86	1	80.6
E6	GI-ME-N_not amplified	TUCR334	31.14	1	80.6
H5	LAN5_amplified	TUCR334	24.8	1	79.3
H6	LAN5_amplified	TUCR334	25.62	1	79.1
F5	LAN5_not amplified	TUCR334	27.57	1	80.7
F6	LAN5_not amplified	TUCR334	28.15	1	80.6
G7	GI-ME-N_amplified	TUCR335	25.14	1	80.1
G8	GI-ME-N_amplified	TUCR335	25.16	1	80.1

E7	GI-ME-N_not amplified	TUCR335	27.42	1	80
E8	GI-ME-N_not amplified	TUCR335	27.58	1	80
H7	LAN5_amplified	TUCR335	23.94	1	80.2
H8	LAN5_amplified	TUCR335	24	1	80.3
F7	LAN5_not amplified	TUCR335	25.44	1	79.9
F8	LAN5_not amplified	TUCR335		1	80
G10	GI-ME-N_amplified	TUCR336	22.8	1	75.5
G9	GI-ME-N_amplified	TUCR336	22.7	1	75.4
E10	GI-ME-N_not amplified	TUCR336	25.21	1	75.5
E9	GI-ME-N_not amplified	TUCR336	24.99	1	75.4
H10	LAN5_amplified	TUCR336		1	75.8
H9	LAN5_amplified	TUCR336	40	1	75.5
F10	LAN5_not amplified	TUCR336		1	75.1
F9	LAN5_not amplified	TUCR336	40	1	75.4
G11	GI-ME-N_amplified	TUCR337	33.73	1	76.8
G12	GI-ME-N_amplified	TUCR337	33.75	1	77
E11	GI-ME-N_not amplified	TUCR337	33.46	1	76.8
E12	GI-ME-N_not amplified	TUCR337		1	77
H11	LAN5_amplified	TUCR337	22.97	0	
H12	LAN5_amplified	TUCR337	22.99	0	
F11	LAN5_not amplified	TUCR337	24.54	0	
F12	LAN5_not amplified	TUCR337	24.7	0	
C1	GI-ME-N_amplified	TUCR338	86.4	1	86
C2	GI-ME-N_amplified	TUCR338	86.4	1	85.8
A1	GI-ME-N_not amplified	TUCR338	86.4	1	86.1
A2	GI-ME-N_not amplified	TUCR338	86.4	1	85.9
D1	LAN5_amplified	TUCR338	86.4	1	86.2
D2	LAN5_amplified	TUCR338	86.4	1	85.9
B1	LAN5_not amplified	TUCR338	86.4	1	85.9
B2	LAN5_not amplified	TUCR338	86.4	1	85.7
C3	GI-ME-N_amplified	TUCR339	85	1	83.5
C4	GI-ME-N_amplified	TUCR339	85	1	83.7
A3	GI-ME-N_not amplified	TUCR339	85	1	83.9
A4	GI-ME-N_not amplified	TUCR339	85	1	84
D4	LAN5_amplified	TUCR339	85	0	
D3	LAN5_amplified	TUCR339	85	1	83.5
B3	LAN5_not amplified	TUCR339	85	1	83.5
B4	LAN5_not amplified	TUCR339	85	1	83.6
G5	GI-ME-N_amplified	TUCR34	77.3	1	76.5
G6	GI-ME-N_amplified	TUCR34	77.3	1	76.4
E5	GI-ME-N_not amplified	TUCR34	77.3	1	76.4
E6	GI-ME-N_not amplified	TUCR34	77.3	1	76.3
H5	LAN5_amplified	TUCR34	77.3	1	76.7
H6	LAN5_amplified	TUCR34	77.3	1	76.5
F5	LAN5_not amplified	TUCR34	77.3	1	76.5
F6	LAN5_not amplified	TUCR34	77.3	1	76.3
C5	GI-ME-N_amplified	TUCR340	81.3	1	80.7
C6	GI-ME-N_amplified	TUCR340	81.3	2	75.0, 80.8
A6	GI-ME-N_not amplified	TUCR340	81.3	0	
A5	GI-ME-N_not amplified	TUCR340	81.3	1	80.8
D5	LAN5_amplified	TUCR340	81.3	1	80.8
D6	LAN5_amplified	TUCR340	81.3	1	80.8
B6	LAN5_not amplified	TUCR340	81.3	0	
B5	LAN5_not amplified	TUCR340	81.3	1	80.8
C7	GI-ME-N_amplified	TUCR341	82.9	1	81.5
C8	GI-ME-N_amplified	TUCR341	82.9	1	81.5
A7	GI-ME-N_not amplified	TUCR341	82.9	0	
A8	GI-ME-N_not amplified	TUCR341	82.9	0	
D7	LAN5_amplified	TUCR341	82.9	1	81.3
D8	LAN5_amplified	TUCR341	82.9	1	81.5
B7	LAN5_not amplified	TUCR341	82.9	1	81.2
B8	LAN5_not amplified	TUCR341	82.9	1	81.4
C10	GI-ME-N_amplified	TUCR342	83.7	1	82.6
C9	GI-ME-N_amplified	TUCR342	83.7	1	82.8
A10	GI-ME-N_not amplified	TUCR342	83.7	1	83.1

A9	GI-ME-N_not amplified	TUCR342	83.7	1	83.3
D10	LAN5_amplified	TUCR342	83.7	1	82.6
D9	LAN5_amplified	TUCR342	83.7	1	82.7
B10	LAN5_not amplified	TUCR342	83.7	1	82.8
B9	LAN5_not amplified	TUCR342	83.7	1	83
C11	GI-ME-N_amplified	TUCR343	84.2	1	83.2
C12	GI-ME-N_amplified	TUCR343	84.2	1	83.5
A11	GI-ME-N_not amplified	TUCR343	84.2	1	83.7
A12	GI-ME-N_not amplified	TUCR343	84.2	1	83.7
B12	LAN5_amplified	TUCR343	84.2	1	83.6
D11	LAN5_amplified	TUCR343	84.2	1	83.2
B11	LAN5_not amplified	TUCR343	84.2	1	83.4
D12	LAN5_not amplified	TUCR343	88	1	83.4
G1	GI-ME-N_amplified	TUCR344	88	1	86.3
G2	GI-ME-N_amplified	TUCR344	88	1	86.2
E1	GI-ME-N_not amplified	TUCR344	88	1	86.1
E2	GI-ME-N_not amplified	TUCR344	88	1	85.8
F1	LAN5_amplified	TUCR344	88	1	86.2
H2	LAN5_amplified	TUCR344	88	1	86.4
F2	LAN5_not amplified	TUCR344	88	1	86
H1	LAN5_not amplified	TUCR344	88	1	86.7
G3	GI-ME-N_amplified	TUCR345	77.1	1	77.8
G4	GI-ME-N_amplified	TUCR345	77.1	1	77.9
E3	GI-ME-N_not amplified	TUCR345	77.1	1	77.6
E4	GI-ME-N_not amplified	TUCR345	77.1	1	77.6
F3	LAN5_amplified	TUCR345	77.1	1	77.7
H4	LAN5_amplified	TUCR345	77.1	1	78
F4	LAN5_not amplified	TUCR345	77.1	1	77.7
H3	LAN5_not amplified	TUCR345	77.1	1	78
G5	GI-ME-N_amplified	TUCR346	85.3	0	
G6	GI-ME-N_amplified	TUCR346	85.3	0	
E6	GI-ME-N_not amplified	TUCR346	85.3	0	
E5	GI-ME-N_not amplified	TUCR346	85.3	1	85
F6	LAN5_amplified	TUCR346	85.3	1	85
H5	LAN5_amplified	TUCR346	85.3	1	85.2
F5	LAN5_not amplified	TUCR346	85.3	1	85
H6	LAN5_not amplified	TUCR346	85.3	1	85.1
G7	GI-ME-N_amplified	TUCR347	77.8	1	75
G8	GI-ME-N_amplified	TUCR347	77.8	1	75.1
E7	GI-ME-N_not amplified	TUCR347	77.8	1	75.1
E8	GI-ME-N_not amplified	TUCR347	77.8	1	75.2
F8	LAN5_amplified	TUCR347	77.8	1	75.2
H7	LAN5_amplified	TUCR347	77.8	1	75.3
F7	LAN5_not amplified	TUCR347	77.8	1	75.1
H8	LAN5_not amplified	TUCR347	77.8	1	75.3
G10	GI-ME-N_amplified	TUCR348	74.4	1	73.9
G9	GI-ME-N_amplified	TUCR348	74.4	1	74
E9	GI-ME-N_not amplified	TUCR348	74.4	1	73.6
E10	GI-ME-N_not amplified	TUCR348	74.4	2	73.2, 76.1
F10	LAN5_amplified	TUCR348	74.4	1	73.7
H9	LAN5_amplified	TUCR348	74.4	1	74.1
F9	LAN5_not amplified	TUCR348	74.4	1	74.1
H10	LAN5_not amplified	TUCR348	74.4	2	73.5, 76.2
G11	GI-ME-N_amplified	TUCR349	77.6	1	78.8
G12	GI-ME-N_amplified	TUCR349	77.6	1	79
E11	GI-ME-N_not amplified	TUCR349	77.6	1	78.7
E12	GI-ME-N_not amplified	TUCR349	77.6	1	78.8
H11	LAN5_amplified	TUCR349	77.6	1	79.1
H12	LAN5_amplified	TUCR349	77.6	1	79.2
F11	LAN5_not amplified	TUCR349	77.6	1	78.6
F12	LAN5_not amplified	TUCR349	77.6	1	78.8
G7	GI-ME-N_amplified	TUCR35	77.6	1	78
G8	GI-ME-N_amplified	TUCR35	77.6	1	78.2
E7	GI-ME-N_not amplified	TUCR35	77.6	1	77.9
E8	GI-ME-N_not amplified	TUCR35	77.6	1	78

H7	LAN5_amplified	TUCR35	77.6	1	78.3
H8	LAN5_amplified	TUCR35	77.6	1	78.3
F7	LAN5_not amplified	TUCR35	77.6	1	77.9
F8	LAN5_not amplified	TUCR35	77.6	1	78
C2	GI-ME-N_amplified	TUCR350	78.2	0	
C1	GI-ME-N_amplified	TUCR350	78.2	1	78.3
A1	GI-ME-N_not amplified	TUCR350	78.2	0	
A2	GI-ME-N_not amplified	TUCR350	78.2	0	
D2	LAN5_amplified	TUCR350	78.2	0	
D1	LAN5_amplified	TUCR350	78.2	1	78.6
B1	LAN5_not amplified	TUCR350	78.2	0	
B2	LAN5_not amplified	TUCR350	78.2	0	
C3	GI-ME-N_amplified	TUCR351	73.9	1	74.7
C4	GI-ME-N_amplified	TUCR351	73.9	2	74.5, 79.0
A3	GI-ME-N_not amplified	TUCR351	73.9	1	75.1
A4	GI-ME-N_not amplified	TUCR351	73.9	1	75.3
D3	LAN5_amplified	TUCR351	73.9	1	75.1
D4	LAN5_amplified	TUCR351	73.9	1	74.9
B3	LAN5_not amplified	TUCR351	73.9	1	75.1
B4	LAN5_not amplified	TUCR351	73.9	1	75.5
C5	GI-ME-N_amplified	TUCR352	72.7	1	72.6
C6	GI-ME-N_amplified	TUCR352	72.7	1	72.5
A5	GI-ME-N_not amplified	TUCR352	72.7	1	73.2
A6	GI-ME-N_not amplified	TUCR352	72.7	1	73.1
D5	LAN5_amplified	TUCR352	72.7	1	72.7
D6	LAN5_amplified	TUCR352	72.7	1	72.6
B5	LAN5_not amplified	TUCR352	72.7	1	72.9
B6	LAN5_not amplified	TUCR352	72.7	1	72.8
C7	GI-ME-N_amplified	TUCR353	73.2	1	73.6
C8	GI-ME-N_amplified	TUCR353	73.2	2	75.6, 78.9
A8	GI-ME-N_not amplified	TUCR353	73.2	0	
A7	GI-ME-N_not amplified	TUCR353	73.2	1	74
D7	LAN5_amplified	TUCR353	73.2	1	73.2
D8	LAN5_amplified	TUCR353	73.2	1	74.2
B8	LAN5_not amplified	TUCR353	73.2	0	
B7	LAN5_not amplified	TUCR353	73.2	1	73.8
C10	GI-ME-N_amplified	TUCR354	79.3	1	80.1
C9	GI-ME-N_amplified	TUCR354	79.3	1	80.2
A10	GI-ME-N_not amplified	TUCR354	79.3	1	80.2
A9	GI-ME-N_not amplified	TUCR354	79.3	1	80
D10	LAN5_amplified	TUCR354	79.3	1	80
D9	LAN5_amplified	TUCR354	79.3	1	80.1
B10	LAN5_not amplified	TUCR354	79.3	1	79.9
B9	LAN5_not amplified	TUCR354	79.3	1	79.7
C11	GI-ME-N_amplified	TUCR355	79	1	77.4
C12	GI-ME-N_amplified	TUCR355	79	1	77.7
A11	GI-ME-N_not amplified	TUCR355	79	1	78.1
A12	GI-ME-N_not amplified	TUCR355	79	1	78.1
D11	LAN5_amplified	TUCR355	79	1	77.4
D12	LAN5_amplified	TUCR355	79	1	77.7
B11	LAN5_not amplified	TUCR355	79	1	77.8
B12	LAN5_not amplified	TUCR355	79	1	78.1
G1	GI-ME-N_amplified	TUCR356	79.3	1	79.8
G2	GI-ME-N_amplified	TUCR356	79.3	1	80.1
E1	GI-ME-N_not amplified	TUCR356	79.3	1	80.1
E2	GI-ME-N_not amplified	TUCR356	79.3	1	79.8
F1	LAN5_amplified	TUCR356	79.3	1	80.2
F2	LAN5_amplified	TUCR356	79.3	1	80
H1	LAN5_not amplified	TUCR356	79.3	1	80.2
H2	LAN5_not amplified	TUCR356	79.3	1	80.4
G3	GI-ME-N_amplified	TUCR357	67.2	7	
G4	GI-ME-N_amplified	TUCR357	67.2	11	
E3	GI-ME-N_not amplified	TUCR357	67.2	0	
E4	GI-ME-N_not amplified	TUCR357	67.2	0	
F3	LAN5_amplified	TUCR357	67.2	1	67.2

F4	LAN5_amplified	TUCR357	67.2	2	60.8, 63.3
H3	LAN5_not amplified	TUCR357	67.2	1	67.3
H4	LAN5_not amplified	TUCR357	67.2	1	67.5
G5	GI-ME-N_amplified	TUCR358	71.2	1	71.1
G6	GI-ME-N_amplified	TUCR358	71.2	2	70.8, 76.0
E5	GI-ME-N_not amplified	TUCR358	71.2	1	71.1
E6	GI-ME-N_not amplified	TUCR358	71.2	2	70.8, 78.1
H5	LAN5_amplified	TUCR358	71.2	1	71.1
H6	LAN5_amplified	TUCR358	71.2	1	71.2
F5	LAN5_not amplified	TUCR358	71.2	1	71
F6	LAN5_not amplified	TUCR358	71.2	1	71
G7	GI-ME-N_amplified	TUCR359	77.6	1	76.5
G8	GI-ME-N_amplified	TUCR359	77.6	1	77
E7	GI-ME-N_not amplified	TUCR359	77.6	1	76.5
E8	GI-ME-N_not amplified	TUCR359	77.6	1	76.8
H7	LAN5_amplified	TUCR359	77.6	1	76.4
H8	LAN5_amplified	TUCR359	77.6	1	77.3
F7	LAN5_not amplified	TUCR359	77.6	1	76.5
F8	LAN5_not amplified	TUCR359	77.6	1	76.8
G10	GI-ME-N_amplified	TUCR36	80.3	1	80.5
G9	GI-ME-N_amplified	TUCR36	80.3	1	80.6
E10	GI-ME-N_not amplified	TUCR36	80.3	1	80.3
E9	GI-ME-N_not amplified	TUCR36	80.3	1	80.5
H10	LAN5_amplified	TUCR36	80.3	1	80.7
H9	LAN5_amplified	TUCR36	80.3	1	80.8
F10	LAN5_not amplified	TUCR36	80.3	1	80.3
F9	LAN5_not amplified	TUCR36	80.3	1	80.5
G10	GI-ME-N_amplified	TUCR360	79.3	1	78.6
G9	GI-ME-N_amplified	TUCR360	79.3	1	78.4
E10	GI-ME-N_not amplified	TUCR360	79.3	1	78.5
E9	GI-ME-N_not amplified	TUCR360	79.3	1	78.4
H10	LAN5_amplified	TUCR360	79.3	1	78.8
H9	LAN5_amplified	TUCR360	79.3	1	78.7
F10	LAN5_not amplified	TUCR360	79.3	1	78.5
F9	LAN5_not amplified	TUCR360	79.3	1	78.4
G12	GI-ME-N_amplified	TUCR361	77.2	0	
G11	GI-ME-N_amplified	TUCR361	77.2	1	74.7
E12	GI-ME-N_not amplified	TUCR361	77.2	0	
E11	GI-ME-N_not amplified	TUCR361	77.2	1	73.8
H11	LAN5_amplified	TUCR361	77.2	1	73.7
H12	LAN5_amplified	TUCR361	77.2	1	76.8
F11	LAN5_not amplified	TUCR361	77.2	1	78.1
F12	LAN5_not amplified	TUCR361	77.2	1	78.4
C1	GI-ME-N_amplified	TUCR362	79.4	1	79.5
C2	GI-ME-N_amplified	TUCR362	79.4	1	79.3
A1	GI-ME-N_not amplified	TUCR362	79.4	1	79.6
A2	GI-ME-N_not amplified	TUCR362	79.4	1	79.5
D1	LAN5_amplified	TUCR362	79.4	1	78.9
D2	LAN5_amplified	TUCR362	79.4	1	78.6
B1	LAN5_not amplified	TUCR362	79.4	1	79.2
B2	LAN5_not amplified	TUCR362	79.4	1	79
C3	GI-ME-N_amplified	TUCR363	76.2	0	
C4	GI-ME-N_amplified	TUCR363	76.2	0	
A3	GI-ME-N_not amplified	TUCR363	76.2	0	
A4	GI-ME-N_not amplified	TUCR363	76.2	0	
D3	LAN5_amplified	TUCR363	76.2	1	77.1
D4	LAN5_amplified	TUCR363	76.2	1	77.3
B3	LAN5_not amplified	TUCR363	76.2	1	77.4
B4	LAN5_not amplified	TUCR363	76.2	1	77.2
C5	GI-ME-N_amplified	TUCR364	74.1	1	74.3
C6	GI-ME-N_amplified	TUCR364	74.1	1	74.3
A5	GI-ME-N_not amplified	TUCR364	74.1	1	74.6
A6	GI-ME-N_not amplified	TUCR364	74.1	1	74.5
D5	LAN5_amplified	TUCR364	74.1	1	74.4
D6	LAN5_amplified	TUCR364	74.1	1	74.2

B5	LAN5_not amplified	TUCR364	74.1	1	74.4
B6	LAN5_not amplified	TUCR364	74.1	1	74.4
C7	GI-ME-N_amplified	TUCR365	81.5	1	80.3
C8	GI-ME-N_amplified	TUCR365	81.5	1	80.5
A7	GI-ME-N_not amplified	TUCR365	81.5	0	
A8	GI-ME-N_not amplified	TUCR365	81.5	0	
D7	LAN5_amplified	TUCR365	81.5	1	80.4
D8	LAN5_amplified	TUCR365	81.5	1	80.5
B7	LAN5_not amplified	TUCR365	81.5	1	80.1
B8	LAN5_not amplified	TUCR365	81.5	1	80.3
C10	GI-ME-N_amplified	TUCR366	80.9	1	80.1
C9	GI-ME-N_amplified	TUCR366	80.9	1	80.2
A10	GI-ME-N_not amplified	TUCR366	80.9	1	80.4
A9	GI-ME-N_not amplified	TUCR366	80.9	1	80.5
D10	LAN5_amplified	TUCR366	80.9	1	80
D9	LAN5_amplified	TUCR366	80.9	1	80.2
B10	LAN5_not amplified	TUCR366	80.9	1	80.1
B9	LAN5_not amplified	TUCR366	80.9	1	80.2
C11	GI-ME-N_amplified	TUCR367	78.1	1	77.3
C12	GI-ME-N_amplified	TUCR367	78.1	1	77.4
A11	GI-ME-N_not amplified	TUCR367	78.1	1	77.8
A12	GI-ME-N_not amplified	TUCR367	78.1	1	77.8
D12	LAN5_amplified	TUCR367	78.1	0	
D11	LAN5_amplified	TUCR367	78.1	1	77.4
B11	LAN5_not amplified	TUCR367	78.1	1	77.6
B12	LAN5_not amplified	TUCR367	78.1	1	72.9
G1	GI-ME-N_amplified	TUCR368	76.6	1	77
G2	GI-ME-N_amplified	TUCR368	76.6	1	76.8
E1	GI-ME-N_not amplified	TUCR368	76.6	1	76.7
E2	GI-ME-N_not amplified	TUCR368	76.6	1	76.4
F1	LAN5_amplified	TUCR368	76.6	1	76.9
F2	LAN5_amplified	TUCR368	76.6	1	76.3
H1	LAN5_not amplified	TUCR368	76.6	1	77.2
H2	LAN5_not amplified	TUCR368	76.6	1	76.9
G3	GI-ME-N_amplified	TUCR369	78.5	1	79.2
G4	GI-ME-N_amplified	TUCR369	78.5	1	79.2
E3	GI-ME-N_not amplified	TUCR369	78.5	1	79
E4	GI-ME-N_not amplified	TUCR369	78.5	1	79
F3	LAN5_amplified	TUCR369	78.5	1	79.1
F4	LAN5_amplified	TUCR369	78.5	1	79.2
H3	LAN5_not amplified	TUCR369	78.5	1	79.5
H4	LAN5_not amplified	TUCR369	78.5	1	79.5
G11	GI-ME-N_amplified	TUCR37	83.1	1	82.4
G12	GI-ME-N_amplified	TUCR37	83.1	1	82.7
E11	GI-ME-N_not amplified	TUCR37	83.1	1	82.1
E12	GI-ME-N_not amplified	TUCR37	83.1	1	82.4
H11	LAN5_amplified	TUCR37	83.1	1	82.7
H12	LAN5_amplified	TUCR37	83.1	1	82.8
F11	LAN5_not amplified	TUCR37	83.1	1	82.1
F12	LAN5_not amplified	TUCR37	83.1	1	82.5
G5	GI-ME-N_amplified	TUCR370	78.2	1	80.2
G6	GI-ME-N_amplified	TUCR370	78.2	2	76.9, 79.1
E5	GI-ME-N_not amplified	TUCR370	78.2	1	77.1
E6	GI-ME-N_not amplified	TUCR370	78.2	1	76.8
H5	LAN5_amplified	TUCR370	78.2	1	77.3
H6	LAN5_amplified	TUCR370	78.2	1	77.2
F5	LAN5_not amplified	TUCR370	78.2	1	77.2
F6	LAN5_not amplified	TUCR370	78.2	1	77
G7	GI-ME-N_amplified	TUCR371	79.6	1	78.6
G8	GI-ME-N_amplified	TUCR371	79.6	1	78.7
E7	GI-ME-N_not amplified	TUCR371	79.6	1	78.6
E8	GI-ME-N_not amplified	TUCR371	79.6	1	78.4
H7	LAN5_amplified	TUCR371	79.6	1	78.8
H8	LAN5_amplified	TUCR371	79.6	1	78.8
F7	LAN5_not amplified	TUCR371	79.6	1	78.5

F8	LAN5_not amplified	TUCR371	79.6	1	78.6
G10	GI-ME-N_amplified	TUCR372	79.9	1	78.5
G9	GI-ME-N_amplified	TUCR372	79.9	1	78.7
E10	GI-ME-N_not amplified	TUCR372	79.9	1	78.4
E9	GI-ME-N_not amplified	TUCR372	79.9	1	78.6
H10	LAN5_amplified	TUCR372	79.9	1	78.7
H9	LAN5_amplified	TUCR372	79.9	1	78.9
F10	LAN5_not amplified	TUCR372	79.9	1	78.4
F9	LAN5_not amplified	TUCR372	79.9	1	78.6
G11	GI-ME-N_amplified	TUCR373	78.2	1	76.8
G12	GI-ME-N_amplified	TUCR373	78.2	1	77.1
E11	GI-ME-N_not amplified	TUCR373	78.2	1	75.1
E12	GI-ME-N_not amplified	TUCR373	78.2	1	75.2
H11	LAN5_amplified	TUCR373	78.2	1	75.2
H12	LAN5_amplified	TUCR373	78.2	1	75.4
F11	LAN5_not amplified	TUCR373	78.2	1	75.2
F12	LAN5_not amplified	TUCR373	78.2	1	75.6
C1	GI-ME-N_amplified	TUCR374	76.8	1	76.8
C2	GI-ME-N_amplified	TUCR374	76.8	1	76.4
A1	GI-ME-N_not amplified	TUCR374	76.8	1	77.1
A2	GI-ME-N_not amplified	TUCR374	76.8	1	77
D1	LAN5_amplified	TUCR374	76.8	1	76.8
D2	LAN5_amplified	TUCR374	76.8	2	76.9, 78.9
B1	LAN5_not amplified	TUCR374	76.8	1	74.5
B2	LAN5_not amplified	TUCR374	76.8	2	74.4, 76.8
C3	GI-ME-N_amplified	TUCR375	75.7	1	78.2
C4	GI-ME-N_amplified	TUCR375	75.7	1	75.8
A3	GI-ME-N_not amplified	TUCR375	75.7	1	78.9
A4	GI-ME-N_not amplified	TUCR375	75.7	1	78
D3	LAN5_amplified	TUCR375	75.7	1	76
D4	LAN5_amplified	TUCR375	75.7	1	77.6
B3	LAN5_not amplified	TUCR375	75.7	1	77.3
B4	LAN5_not amplified	TUCR375	75.7	1	75.5
C5	GI-ME-N_amplified	TUCR376	82.1	1	82.3
C6	GI-ME-N_amplified	TUCR376	82.1	1	82.1
A5	GI-ME-N_not amplified	TUCR376	82.1	1	82.5
A6	GI-ME-N_not amplified	TUCR376	82.1	1	82.4
D5	LAN5_amplified	TUCR376	82.1	1	82.3
D6	LAN5_amplified	TUCR376	82.1	1	82.1
B5	LAN5_not amplified	TUCR376	82.1	1	82.3
B6	LAN5_not amplified	TUCR376	82.1	1	82.1
C7	GI-ME-N_amplified	TUCR377	78.8	1	78.9
C8	GI-ME-N_amplified	TUCR377	78.8	1	79
A7	GI-ME-N_not amplified	TUCR377	78.8	1	79.1
A8	GI-ME-N_not amplified	TUCR377	78.8	1	79.2
D7	LAN5_amplified	TUCR377	78.8	1	78.9
D8	LAN5_amplified	TUCR377	78.8	1	78.9
B7	LAN5_not amplified	TUCR377	78.8	1	78.9
B8	LAN5_not amplified	TUCR377	78.8	1	79
C10	GI-ME-N_amplified	TUCR378	84.5	1	83.9
C9	GI-ME-N_amplified	TUCR378	84.5	1	84.1
A10	GI-ME-N_not amplified	TUCR378	84.5	1	84.3
A9	GI-ME-N_not amplified	TUCR378	84.5	1	84.5
D10	LAN5_amplified	TUCR378	84.5	1	83.8
D9	LAN5_amplified	TUCR378	84.5	1	84
B10	LAN5_not amplified	TUCR378	84.5	1	83.9
B9	LAN5_not amplified	TUCR378	84.5	1	84.1
C11	GI-ME-N_amplified	TUCR379	80.3	1	79.7
C12	GI-ME-N_amplified	TUCR379	80.3	1	80.1
A11	GI-ME-N_not amplified	TUCR379	80.3	1	80.5
A12	GI-ME-N_not amplified	TUCR379	80.3	1	80.8
D11	LAN5_amplified	TUCR379	80.3	1	80.1
D12	LAN5_amplified	TUCR379	80.3	1	80.2
B11	LAN5_not amplified	TUCR379	80.3	1	80.3
B12	LAN5_not amplified	TUCR379	80.3	1	80.5

C1	GI-ME-N_amplified	TUCR38	77	1	76.3
C2	GI-ME-N_amplified	TUCR38	77	1	76.1
A1	GI-ME-N_not amplified	TUCR38	77	1	76.5
A2	GI-ME-N_not amplified	TUCR38	77	1	76.4
D1	LAN5_amplified	TUCR38	77	0	
D2	LAN5_amplified	TUCR38	77	0	
B1	LAN5_not amplified	TUCR38	77	0	
B2	LAN5_not amplified	TUCR38	77	0	
G2	GI-ME-N_amplified	TUCR380	76.9	0	
G1	GI-ME-N_amplified	TUCR380	76.9	1	73
E2	GI-ME-N_not amplified	TUCR380	76.9	1	72.8
E1	GI-ME-N_not amplified	TUCR380	76.9	2	72.7, 77.5
F1	LAN5_amplified	TUCR380	76.9	1	73.2
F2	LAN5_amplified	TUCR380	76.9	2	73.2, 76.1
H1	LAN5_not amplified	TUCR380	76.9	1	73.2
H2	LAN5_not amplified	TUCR380	76.9	2	78.9, 80.4
G3	GI-ME-N_amplified	TUCR381	83.6	1	73
G4	GI-ME-N_amplified	TUCR381	83.6	1	73
E4	GI-ME-N_not amplified	TUCR381	83.6	0	
E3	GI-ME-N_not amplified	TUCR381	83.6	1	72.8
F3	LAN5_amplified	TUCR381	83.6	1	78.7
F4	LAN5_amplified	TUCR381	83.6	1	78.8
H3	LAN5_not amplified	TUCR381	83.6	1	78.8
H4	LAN5_not amplified	TUCR381	83.6	1	78.9
G5	GI-ME-N_amplified	TUCR382	79.1	1	79.4
G6	GI-ME-N_amplified	TUCR382	79.1	1	79.1
E5	GI-ME-N_not amplified	TUCR382	79.1	1	79.3
E6	GI-ME-N_not amplified	TUCR382	79.1	1	79.1
H5	LAN5_amplified	TUCR382	79.1	1	79.6
H6	LAN5_amplified	TUCR382	79.1	1	79.4
F5	LAN5_not amplified	TUCR382	79.1	1	79.4
F6	LAN5_not amplified	TUCR382	79.1	1	79.1
G7	GI-ME-N_amplified	TUCR383	74.8	0	
G8	GI-ME-N_amplified	TUCR383	74.8	1	75.7
E7	GI-ME-N_not amplified	TUCR383	74.8	1	75.2
E8	GI-ME-N_not amplified	TUCR383	74.8	1	75.4
H7	LAN5_amplified	TUCR383	74.8	1	75.4
H8	LAN5_amplified	TUCR383	74.8	1	75.5
F7	LAN5_not amplified	TUCR383	74.8	0	
F8	LAN5_not amplified	TUCR383	74.8	1	75.4
G10	GI-ME-N_amplified	TUCR384	79.3	1	77.7
G9	GI-ME-N_amplified	TUCR384	79.3	1	77.9
E10	GI-ME-N_not amplified	TUCR384	79.3	1	77.7
E9	GI-ME-N_not amplified	TUCR384	79.3	1	77.8
H10	LAN5_amplified	TUCR384	79.3	1	77.9
H9	LAN5_amplified	TUCR384	79.3	1	78
F10	LAN5_not amplified	TUCR384	79.3	1	77.7
F9	LAN5_not amplified	TUCR384	79.3	1	77.8
G11	GI-ME-N_amplified	TUCR385	75.6	1	76.2
G12	GI-ME-N_amplified	TUCR385	75.6	1	76.4
E11	GI-ME-N_not amplified	TUCR385	75.6	1	76
E12	GI-ME-N_not amplified	TUCR385	75.6	1	76.1
H11	LAN5_amplified	TUCR385	75.6	1	76.4
H12	LAN5_amplified	TUCR385	75.6	1	76.6
F11	LAN5_not amplified	TUCR385	75.6	1	75.9
F12	LAN5_not amplified	TUCR385	75.6	1	76.2
C1	GI-ME-N_amplified	TUCR386	72.7	1	73
C2	GI-ME-N_amplified	TUCR386	72.7	1	72.9
A1	GI-ME-N_not amplified	TUCR386	72.7	1	77.7
A2	GI-ME-N_not amplified	TUCR386	72.7	1	78.6
D2	LAN5_amplified	TUCR386	72.7	0	
D1	LAN5_amplified	TUCR386	72.7	1	73.3
B1	LAN5_not amplified	TUCR386	72.7	1	73.1
B2	LAN5_not amplified	TUCR386	72.7	1	73.3
C3	GI-ME-N_amplified	TUCR387	85.9	1	84.3

C4	GI-ME-N_amplified	TUCR387	85.9	1	84.5
A3	GI-ME-N_not amplified	TUCR387	85.9	1	84.7
A4	GI-ME-N_not amplified	TUCR387	85.9	1	84.8
D3	LAN5_amplified	TUCR387	85.9	1	84.4
D4	LAN5_amplified	TUCR387	85.9	1	84.5
B3	LAN5_not amplified	TUCR387	85.9	1	84.4
B4	LAN5_not amplified	TUCR387	85.9	1	84.6
C5	GI-ME-N_amplified	TUCR388	79.3	1	79.1
C6	GI-ME-N_amplified	TUCR388	79.3	1	79
A5	GI-ME-N_not amplified	TUCR388	79.3	1	79.3
A6	GI-ME-N_not amplified	TUCR388	79.3	1	79.3
D5	LAN5_amplified	TUCR388	79.3	1	79.1
D6	LAN5_amplified	TUCR388	79.3	1	79
B5	LAN5_not amplified	TUCR388	79.3	1	79.1
B6	LAN5_not amplified	TUCR388	79.3	1	79
C7	GI-ME-N_amplified	TUCR389	76.6	1	77.7
C8	GI-ME-N_amplified	TUCR389	76.6	1	77.8
A8	GI-ME-N_not amplified	TUCR389	76.6	1	76.5
A7	GI-ME-N_not amplified	TUCR389	76.6	2	76.0, 77.7
D7	LAN5_amplified	TUCR389	76.6	1	77.8
D8	LAN5_amplified	TUCR389	76.6	1	77.7
B7	LAN5_not amplified	TUCR389	76.6	1	76.1
B8	LAN5_not amplified	TUCR389	76.6	1	76.4
C3	GI-ME-N_amplified	TUCR39	82.2	1	81
C4	GI-ME-N_amplified	TUCR39	82.2	1	81.2
A3	GI-ME-N_not amplified	TUCR39	82.2	1	81.3
A4	GI-ME-N_not amplified	TUCR39	82.2	1	81.4
D3	LAN5_amplified	TUCR39	82.2	0	
D4	LAN5_amplified	TUCR39	82.2	1	71
B3	LAN5_not amplified	TUCR39	82.2	1	71
B4	LAN5_not amplified	TUCR39	82.2	1	71.8
C10	GI-ME-N_amplified	TUCR390	77.9	1	77.3
C9	GI-ME-N_amplified	TUCR390	77.9	1	77.4
A10	GI-ME-N_not amplified	TUCR390	77.9	1	77.7
A9	GI-ME-N_not amplified	TUCR390	77.9	1	77.9
D10	LAN5_amplified	TUCR390	77.9	1	77.3
D9	LAN5_amplified	TUCR390	77.9	1	77.4
B10	LAN5_not amplified	TUCR390	77.9	1	77.4
B9	LAN5_not amplified	TUCR390	77.9	1	77.6
C11	GI-ME-N_amplified	TUCR391	78.5	1	77.7
C12	GI-ME-N_amplified	TUCR391	78.5	1	77.9
A11	GI-ME-N_not amplified	TUCR391	78.5	1	78.1
A12	GI-ME-N_not amplified	TUCR391	78.5	1	78.2
D11	LAN5_amplified	TUCR391	78.5	1	77.7
D12	LAN5_amplified	TUCR391	78.5	1	77.9
B11	LAN5_not amplified	TUCR391	78.5	1	77.8
B12	LAN5_not amplified	TUCR391	78.5	1	77.9
G1	GI-ME-N_amplified	TUCR392	78.5	1	79.4
G2	GI-ME-N_amplified	TUCR392	78.5	1	79.2
E1	GI-ME-N_not amplified	TUCR392	78.5	1	79.3
E2	GI-ME-N_not amplified	TUCR392	78.5	1	79
F1	LAN5_amplified	TUCR392	78.5	1	79.4
F2	LAN5_amplified	TUCR392	78.5	1	79.1
H1	LAN5_not amplified	TUCR392	78.5	1	79.6
H2	LAN5_not amplified	TUCR392	78.5	1	79.4
G3	GI-ME-N_amplified	TUCR393	83	1	83.3
G4	GI-ME-N_amplified	TUCR393	83	1	83.3
E3	GI-ME-N_not amplified	TUCR393	83	1	82.9
E4	GI-ME-N_not amplified	TUCR393	83	1	82.9
F3	LAN5_amplified	TUCR393	83	1	83
F4	LAN5_amplified	TUCR393	83	1	83.1
H3	LAN5_not amplified	TUCR393	83	1	83.5
H4	LAN5_not amplified	TUCR393	83	1	83.5
G5	GI-ME-N_amplified	TUCR394	77.7	0	
G6	GI-ME-N_amplified	TUCR394	77.7	2	80.6, 84.0

E5	GI-ME-N_not amplified	TUCR394	77.7	0	
E6	GI-ME-N_not amplified	TUCR394	77.7	0	
H5	LAN5_amplified	TUCR394	77.7	1	77.4
H6	LAN5_amplified	TUCR394	77.7	1	77.6
F5	LAN5_not amplified	TUCR394	77.7	1	77.5
F6	LAN5_not amplified	TUCR394	77.7	1	76.8
G7	GI-ME-N_amplified	TUCR395	78.3	1	79
G8	GI-ME-N_amplified	TUCR395	78.3	1	79.1
E7	GI-ME-N_not amplified	TUCR395	78.3	1	78.9
E8	GI-ME-N_not amplified	TUCR395	78.3	1	78.9
H7	LAN5_amplified	TUCR395	78.3	1	79.2
H8	LAN5_amplified	TUCR395	78.3	1	79.2
F7	LAN5_not amplified	TUCR395	78.3	1	78.9
F8	LAN5_not amplified	TUCR395	78.3	1	79
G10	GI-ME-N_amplified	TUCR396	78.4	1	77.7
G9	GI-ME-N_amplified	TUCR396	78.4	1	77.9
E10	GI-ME-N_not amplified	TUCR396	78.4	1	77.7
E9	GI-ME-N_not amplified	TUCR396	78.4	1	77.8
H10	LAN5_amplified	TUCR396	78.4	1	78.1
H9	LAN5_amplified	TUCR396	78.4	1	77.9
F10	LAN5_not amplified	TUCR396	78.4	1	77.9
F9	LAN5_not amplified	TUCR396	78.4	1	77.8
G11	GI-ME-N_amplified	TUCR397	84.2	1	82.7
G12	GI-ME-N_amplified	TUCR397	84.2	1	82.9
E11	GI-ME-N_not amplified	TUCR397	84.2	1	82.6
E12	GI-ME-N_not amplified	TUCR397	84.2	1	82.8
H11	LAN5_amplified	TUCR397	84.2	1	82.9
H12	LAN5_amplified	TUCR397	84.2	1	83.1
F11	LAN5_not amplified	TUCR397	84.2	1	82.6
F12	LAN5_not amplified	TUCR397	84.2	1	82.8
C3	GI-ME-N_amplified	TUCR398	89.1	1	86.9
C4	GI-ME-N_amplified	TUCR398	89.1	1	87
A3	GI-ME-N_not amplified	TUCR398	89.1	0	
A4	GI-ME-N_not amplified	TUCR398	89.1	0	
D3	LAN5_amplified	TUCR398	89.1	1	87.2
D4	LAN5_amplified	TUCR398	89.1	1	86.7
B3	LAN5_not amplified	TUCR398	89.1	0	
B4	LAN5_not amplified	TUCR398	89.1	1	81.4
C5	GI-ME-N_amplified	TUCR399	74.6	1	73.7
C6	GI-ME-N_amplified	TUCR399	74.6	1	73.8
A5	GI-ME-N_not amplified	TUCR399	74.6	1	74.3
A6	GI-ME-N_not amplified	TUCR399	74.6	1	74.2
D5	LAN5_amplified	TUCR399	74.6	1	73.8
D6	LAN5_amplified	TUCR399	74.6	1	73.8
B5	LAN5_not amplified	TUCR399	74.6	1	74.1
B6	LAN5_not amplified	TUCR399	74.6	1	73.9
C10	GI-ME-N_amplified	TUCR4	79.1	1	76.9
C9	GI-ME-N_amplified	TUCR4	79.1	1	77.1
A10	GI-ME-N_not amplified	TUCR4	79.1	1	77.4
A9	GI-ME-N_not amplified	TUCR4	79.1	1	77.5
D10	LAN5_amplified	TUCR4	79.1	1	76.9
D9	LAN5_amplified	TUCR4	79.1	1	77
B10	LAN5_not amplified	TUCR4	79.1	1	77.2
B9	LAN5_not amplified	TUCR4	79.1	1	77.4
C5	GI-ME-N_amplified	TUCR40	79	1	78
C6	GI-ME-N_amplified	TUCR40	79	1	77.9
A5	GI-ME-N_not amplified	TUCR40	79	1	78.4
A6	GI-ME-N_not amplified	TUCR40	79	1	78.3
D5	LAN5_amplified	TUCR40	79	1	78.1
D6	LAN5_amplified	TUCR40	79	1	77.9
B5	LAN5_not amplified	TUCR40	79	1	78.2
B6	LAN5_not amplified	TUCR40	79	1	78.1
C7	GI-ME-N_amplified	TUCR400	79	1	77.9
C8	GI-ME-N_amplified	TUCR400	79	1	77.7
A7	GI-ME-N_not amplified	TUCR400	79	1	78.1

A8	GI-ME-N_not amplified	TUCR400	79	1	78.2
D8	LAN5_amplified	TUCR400	79	0	
D7	LAN5_amplified	TUCR400	79	1	77.6
B7	LAN5_not amplified	TUCR400	79	1	77.9
B8	LAN5_not amplified	TUCR400	79	1	78
C10	GI-ME-N_amplified	TUCR401	79.3	1	77.7
C9	GI-ME-N_amplified	TUCR401	79.3	1	78
A10	GI-ME-N_not amplified	TUCR401	79.3	1	78.2
A9	GI-ME-N_not amplified	TUCR401	79.3	1	78.2
D10	LAN5_amplified	TUCR401	79.3	0	
D9	LAN5_amplified	TUCR401	79.3	0	
B10	LAN5_not amplified	TUCR401	79.3	0	
B9	LAN5_not amplified	TUCR401	79.3	1	74.1
C11	GI-ME-N_amplified	TUCR402	77.8	1	76.5
C12	GI-ME-N_amplified	TUCR402	77.8	1	76.7
A11	GI-ME-N_not amplified	TUCR402	77.8	1	76.9
A12	GI-ME-N_not amplified	TUCR402	77.8	1	77
D11	LAN5_amplified	TUCR402	77.8	1	76.5
D12	LAN5_amplified	TUCR402	77.8	1	76.5
B11	LAN5_not amplified	TUCR402	77.8	1	76.6
B12	LAN5_not amplified	TUCR402	77.8	1	76.8
G1	GI-ME-N_amplified	TUCR403	81.4	1	80.6
G2	GI-ME-N_amplified	TUCR403	81.4	1	80.4
E1	GI-ME-N_not amplified	TUCR403	81.4	1	80.4
E2	GI-ME-N_not amplified	TUCR403	81.4	1	80.2
F1	LAN5_amplified	TUCR403	81.4	1	80.2
F2	LAN5_amplified	TUCR403	81.4	1	80.2
H1	LAN5_not amplified	TUCR403	81.4	1	80.7
H2	LAN5_not amplified	TUCR403	81.4	1	80.6
G3	GI-ME-N_amplified	TUCR404	73.7	1	74.9
G4	GI-ME-N_amplified	TUCR404	73.7	1	75
E3	GI-ME-N_not amplified	TUCR404	73.7	1	74.7
E4	GI-ME-N_not amplified	TUCR404	73.7	1	74.7
F3	LAN5_amplified	TUCR404	73.7	1	74.7
F4	LAN5_amplified	TUCR404	73.7	1	74.7
H3	LAN5_not amplified	TUCR404	73.7	1	75.1
H4	LAN5_not amplified	TUCR404	73.7	1	75.1
G5	GI-ME-N_amplified	TUCR405	74.4	1	73
G6	GI-ME-N_amplified	TUCR405	74.4	1	73.3
E5	GI-ME-N_not amplified	TUCR405	74.4	1	74.9
E6	GI-ME-N_not amplified	TUCR405	74.4	1	74.8
H6	LAN5_amplified	TUCR405	74.4	0	
H5	LAN5_amplified	TUCR405	74.4	1	74.8
F5	LAN5_not amplified	TUCR405	74.4	0	
F6	LAN5_not amplified	TUCR405	74.4	0	
G7	GI-ME-N_amplified	TUCR406	77.7	1	78
G8	GI-ME-N_amplified	TUCR406	77.7	1	78.1
E7	GI-ME-N_not amplified	TUCR406	77.7	1	77.8
E8	GI-ME-N_not amplified	TUCR406	77.7	1	78
H7	LAN5_amplified	TUCR406	77.7	1	78.2
H8	LAN5_amplified	TUCR406	77.7	1	78.2
F7	LAN5_not amplified	TUCR406	77.7	1	77.9
F8	LAN5_not amplified	TUCR406	77.7	1	78.1
G10	GI-ME-N_amplified	TUCR407	81.1	1	79.9
G9	GI-ME-N_amplified	TUCR407	81.1	1	80.1
E10	GI-ME-N_not amplified	TUCR407	81.1	1	79.9
E9	GI-ME-N_not amplified	TUCR407	81.1	1	80
H10	LAN5_amplified	TUCR407	81.1	1	80.1
H9	LAN5_amplified	TUCR407	81.1	1	80.2
F10	LAN5_not amplified	TUCR407	81.1	1	79.8
F9	LAN5_not amplified	TUCR407	81.1	1	80
G11	GI-ME-N_amplified	TUCR408	*83.2*	1	82.4
G12	GI-ME-N_amplified	TUCR408	*83.2*	1	82.6
E11	GI-ME-N_not amplified	TUCR408	*83.2*	1	82.3
E12	GI-ME-N_not amplified	TUCR408	*83.2*	1	82.5

H11	LAN5_amplified	TUCR408	*83.2*	1	82.6
H12	LAN5_amplified	TUCR408	*83.2*	1	82.7
F11	LAN5_not amplified	TUCR408	*83.2*	1	82.3
F12	LAN5_not amplified	TUCR408	*83.2*	1	82.5
C1	GI-ME-N_amplified	TUCR409	78.8	1	79.5
C2	GI-ME-N_amplified	TUCR409	78.8	1	79.3
A1	GI-ME-N_not amplified	TUCR409	78.8	1	79.9
A2	GI-ME-N_not amplified	TUCR409	78.8	1	79.6
D1	LAN5_amplified	TUCR409	78.8	1	79.6
D2	LAN5_amplified	TUCR409	78.8	1	79.4
B1	LAN5_not amplified	TUCR409	78.8	1	79.6
B2	LAN5_not amplified	TUCR409	78.8	1	79.4
C7	GI-ME-N_amplified	TUCR41	79	1	78.5
C8	GI-ME-N_amplified	TUCR41	79	1	78.6
A7	GI-ME-N_not amplified	TUCR41	79	1	78.9
A8	GI-ME-N_not amplified	TUCR41	79	1	78.9
D7	LAN5_amplified	TUCR41	79	1	78.6
D8	LAN5_amplified	TUCR41	79	1	78.6
B7	LAN5_not amplified	TUCR41	79	1	78.7
B8	LAN5_not amplified	TUCR41	79	1	78.8
C3	GI-ME-N_amplified	TUCR410	77.3	1	77.1
C4	GI-ME-N_amplified	TUCR410	77.3	1	77.3
A3	GI-ME-N_not amplified	TUCR410	77.3	1	77.4
A4	GI-ME-N_not amplified	TUCR410	77.3	1	77.5
D3	LAN5_amplified	TUCR410	77.3	1	77.3
D4	LAN5_amplified	TUCR410	77.3	1	77.3
B3	LAN5_not amplified	TUCR410	77.3	1	77
B4	LAN5_not amplified	TUCR410	77.3	1	77.3
C5	GI-ME-N_amplified	TUCR411	80.4	1	80.8
C6	GI-ME-N_amplified	TUCR411	80.4	1	80.7
A5	GI-ME-N_not amplified	TUCR411	80.4	1	81
A6	GI-ME-N_not amplified	TUCR411	80.4	1	80.9
D5	LAN5_amplified	TUCR411	80.4	1	80.8
D6	LAN5_amplified	TUCR411	80.4	1	80.7
B5	LAN5_not amplified	TUCR411	80.4	1	80.9
B6	LAN5_not amplified	TUCR411	80.4	1	80.8
C7	GI-ME-N_amplified	TUCR412	77.2	1	77
C8	GI-ME-N_amplified	TUCR412	77.2	1	77.1
A7	GI-ME-N_not amplified	TUCR412	77.2	1	77.3
A8	GI-ME-N_not amplified	TUCR412	77.2	1	77.4
D7	LAN5_amplified	TUCR412	77.2	1	77
D8	LAN5_amplified	TUCR412	77.2	1	77
B7	LAN5_not amplified	TUCR412	77.2	1	77.1
B8	LAN5_not amplified	TUCR412	77.2	1	77.2
C10	GI-ME-N_amplified	TUCR413	88.6	1	87
C9	GI-ME-N_amplified	TUCR413	88.6	1	87.2
A10	GI-ME-N_not amplified	TUCR413	88.6	1	87.5
A9	GI-ME-N_not amplified	TUCR413	88.6	1	87.7
D10	LAN5_amplified	TUCR413	88.6	1	87
D9	LAN5_amplified	TUCR413	88.6	1	87.1
B10	LAN5_not amplified	TUCR413	88.6	1	87.2
B9	LAN5_not amplified	TUCR413	88.6	1	87.4
C11	GI-ME-N_amplified	TUCR414	90	1	89.1
C12	GI-ME-N_amplified	TUCR414	90	1	89.4
A11	GI-ME-N_not amplified	TUCR414	90	1	89.6
A12	GI-ME-N_not amplified	TUCR414	90	1	89.7
D11	LAN5_amplified	TUCR414	90	1	89.1
D12	LAN5_amplified	TUCR414	90	1	89.3
B11	LAN5_not amplified	TUCR414	90	1	89.3
B12	LAN5_not amplified	TUCR414	90	1	89.5
G1	GI-ME-N_amplified	TUCR415	81.2	1	81
G2	GI-ME-N_amplified	TUCR415	81.2	1	80.8
E1	GI-ME-N_not amplified	TUCR415	81.2	1	80.8
E2	GI-ME-N_not amplified	TUCR415	81.2	1	80.6
F1	LAN5_amplified	TUCR415	81.2	1	69.8

F2	LAN5_amplified	TUCR415	81.2	1	69.6
H1	LAN5_not amplified	TUCR415	81.2	1	74.3
H2	LAN5_not amplified	TUCR415	81.2	1	69.9
G3	GI-ME-N_amplified	TUCR416	80.2	1	80.3
G4	GI-ME-N_amplified	TUCR416	80.2	1	80.4
E3	GI-ME-N_not amplified	TUCR416	80.2	1	80.3
E4	GI-ME-N_not amplified	TUCR416	80.2	1	80.3
F3	LAN5_amplified	TUCR416	80.2	0	
F4	LAN5_amplified	TUCR416	80.2	0	
H3	LAN5_not amplified	TUCR416	80.2	0	
H4	LAN5_not amplified	TUCR416	80.2	0	
G5	GI-ME-N_amplified	TUCR417	74.9	1	75.7
G6	GI-ME-N_amplified	TUCR417	74.9	1	75.6
E5	GI-ME-N_not amplified	TUCR417	74.9	1	75.6
E6	GI-ME-N_not amplified	TUCR417	74.9	1	75.5
H5	LAN5_amplified	TUCR417	74.9	1	75.4
H6	LAN5_amplified	TUCR417	74.9	1	73.5
F6	LAN5_not amplified	TUCR417	74.9	0	
F5	LAN5_not amplified	TUCR417	74.9	1	75.5
G7	GI-ME-N_amplified	TUCR418	79.2	1	78.6
G8	GI-ME-N_amplified	TUCR418	79.2	1	78.7
E7	GI-ME-N_not amplified	TUCR418	79.2	1	78.6
E8	GI-ME-N_not amplified	TUCR418	79.2	1	78.6
H7	LAN5_amplified	TUCR418	79.2	1	78.9
H8	LAN5_amplified	TUCR418	79.2	1	78.9
F7	LAN5_not amplified	TUCR418	79.2	1	78.6
F8	LAN5_not amplified	TUCR418	79.2	1	78.6
G10	GI-ME-N_amplified	TUCR419	83.3	1	81.9
G9	GI-ME-N_amplified	TUCR419	83.3	1	82
E10	GI-ME-N_not amplified	TUCR419	83.3	1	81.8
E9	GI-ME-N_not amplified	TUCR419	83.3	1	82
H10	LAN5_amplified	TUCR419	83.3	1	82
H9	LAN5_amplified	TUCR419	83.3	1	82.2
F10	LAN5_not amplified	TUCR419	83.3	1	81.7
F9	LAN5_not amplified	TUCR419	83.3	1	81.9
C9	GI-ME-N_amplified	TUCR42	80.5	2	74.2, 79.8
C10	GI-ME-N_amplified	TUCR42	80.5	3	
A10	GI-ME-N_not amplified	TUCR42	80.5	0	
A9	GI-ME-N_not amplified	TUCR42	80.5	0	
D10	LAN5_amplified	TUCR42	80.5	4	
D9	LAN5_amplified	TUCR42	80.5	6	
B10	LAN5_not amplified	TUCR42	80.5	0	
B9	LAN5_not amplified	TUCR42	80.5	0	
G11	GI-ME-N_amplified	TUCR420	79.7	1	79.1
G12	GI-ME-N_amplified	TUCR420	79.7	1	79.4
E11	GI-ME-N_not amplified	TUCR420	79.7	1	79.1
E12	GI-ME-N_not amplified	TUCR420	79.7	1	79.3
H11	LAN5_amplified	TUCR420	79.7	1	79.4
H12	LAN5_amplified	TUCR420	79.7	1	79.5
F11	LAN5_not amplified	TUCR420	79.7	1	79
F12	LAN5_not amplified	TUCR420	79.7	1	79.3
C1	GI-ME-N_amplified	TUCR421	87	1	85.1
C2	GI-ME-N_amplified	TUCR421	87	1	84.9
A1	GI-ME-N_not amplified	TUCR421	87	1	85.4
A2	GI-ME-N_not amplified	TUCR421	87	1	85.2
D1	LAN5_amplified	TUCR421	87	1	85.3
D2	LAN5_amplified	TUCR421	87	1	85
B1	LAN5_not amplified	TUCR421	87	1	85.2
B2	LAN5_not amplified	TUCR421	87	1	85
C3	GI-ME-N_amplified	TUCR422	87.1	1	85
C4	GI-ME-N_amplified	TUCR422	87.1	1	85.1
A3	GI-ME-N_not amplified	TUCR422	87.1	1	85.3
A4	GI-ME-N_not amplified	TUCR422	87.1	1	85.5
D3	LAN5_amplified	TUCR422	87.1	1	85.1
D4	LAN5_amplified	TUCR422	87.1	1	85.1

B3	LAN5_not amplified	TUCR422	87.1	1	85
B4	LAN5_not amplified	TUCR422	87.1	1	85.2
C5	GI-ME-N_amplified	TUCR423	79.8	1	78.5
C6	GI-ME-N_amplified	TUCR423	79.8	1	78.3
A5	GI-ME-N_not amplified	TUCR423	79.8	1	78.8
A6	GI-ME-N_not amplified	TUCR423	79.8	1	78.7
D5	LAN5_amplified	TUCR423	79.8	1	78.5
D6	LAN5_amplified	TUCR423	79.8	1	78.4
B5	LAN5_not amplified	TUCR423	79.8	1	78.7
B6	LAN5_not amplified	TUCR423	79.8	1	78.4
C7	GI-ME-N_amplified	TUCR424	79.7	1	78.9
C8	GI-ME-N_amplified	TUCR424	79.7	1	79
A7	GI-ME-N_not amplified	TUCR424	79.7	1	79.2
A8	GI-ME-N_not amplified	TUCR424	79.7	1	79.3
D7	LAN5_amplified	TUCR424	79.7	1	78.9
D8	LAN5_amplified	TUCR424	79.7	1	78.9
B7	LAN5_not amplified	TUCR424	79.7	1	79
B8	LAN5_not amplified	TUCR424	79.7	1	79.1
C10	GI-ME-N_amplified	TUCR425	83.6	1	81.6
C9	GI-ME-N_amplified	TUCR425	83.6	1	81.7
A10	GI-ME-N_not amplified	TUCR425	83.6	1	81.9
A9	GI-ME-N_not amplified	TUCR425	83.6	1	82
D10	LAN5_amplified	TUCR425	83.6	1	81.5
D9	LAN5_amplified	TUCR425	83.6	1	81.7
B10	LAN5_not amplified	TUCR425	83.6	1	81.7
B9	LAN5_not amplified	TUCR425	83.6	1	81.9
C11	GI-ME-N_amplified	TUCR426	82.1	1	80.7
C12	GI-ME-N_amplified	TUCR426	82.1	1	81
A11	GI-ME-N_not amplified	TUCR426	82.1	1	81.2
A12	GI-ME-N_not amplified	TUCR426	82.1	1	81.3
D11	LAN5_amplified	TUCR426	82.1	1	80.7
D12	LAN5_amplified	TUCR426	82.1	1	81
B11	LAN5_not amplified	TUCR426	82.1	1	81
B12	LAN5_not amplified	TUCR426	82.1	1	81.1
G1	GI-ME-N_amplified	TUCR427	78.9	1	79.4
G2	GI-ME-N_amplified	TUCR427	78.9	1	79.2
E1	GI-ME-N_not amplified	TUCR427	78.9	1	79.1
E2	GI-ME-N_not amplified	TUCR427	78.9	1	78.8
F1	LAN5_amplified	TUCR427	78.9	1	79.2
F2	LAN5_amplified	TUCR427	78.9	1	79
H1	LAN5_not amplified	TUCR427	78.9	1	79.5
H2	LAN5_not amplified	TUCR427	78.9	1	79.4
G3	GI-ME-N_amplified	TUCR428	83.5	1	83
G4	GI-ME-N_amplified	TUCR428	83.5	1	83
E3	GI-ME-N_not amplified	TUCR428	83.5	1	82.7
E4	GI-ME-N_not amplified	TUCR428	83.5	1	82.6
F3	LAN5_amplified	TUCR428	83.5	1	82.7
F4	LAN5_amplified	TUCR428	83.5	1	78.9
H3	LAN5_not amplified	TUCR428	83.5	1	83.2
H4	LAN5_not amplified	TUCR428	83.5	1	82.8
G5	GI-ME-N_amplified	TUCR429	76.2	1	76.8
G6	GI-ME-N_amplified	TUCR429	76.2	1	76.7
E5	GI-ME-N_not amplified	TUCR429	76.2	1	76.6
E6	GI-ME-N_not amplified	TUCR429	76.2	1	76.5
H5	LAN5_amplified	TUCR429	76.2	1	77
H6	LAN5_amplified	TUCR429	76.2	1	76.8
F5	LAN5_not amplified	TUCR429	76.2	1	76.7
F6	LAN5_not amplified	TUCR429	76.2	1	76.6
C11	GI-ME-N_amplified	TUCR43	81.2	1	79.2
C12	GI-ME-N_amplified	TUCR43	81.2	1	79.4
A11	GI-ME-N_not amplified	TUCR43	81.2	1	79.7
A12	GI-ME-N_not amplified	TUCR43	81.2	1	79.7
D11	LAN5_amplified	TUCR43	81.2	1	79.3
D12	LAN5_amplified	TUCR43	81.2	1	79.3
B11	LAN5_not amplified	TUCR43	81.2	1	79.3

B12	LAN5_not amplified	TUCR43	81.2	1	79.5
G7	GI-ME-N_amplified	TUCR430	79.1	1	79.4
G8	GI-ME-N_amplified	TUCR430	79.1	1	79.5
E7	GI-ME-N_not amplified	TUCR430	79.1	1	79.3
E8	GI-ME-N_not amplified	TUCR430	79.1	1	79.3
H7	LAN5_amplified	TUCR430	79.1	1	79.6
H8	LAN5_amplified	TUCR430	79.1	1	79.6
F7	LAN5_not amplified	TUCR430	79.1	1	79.3
F8	LAN5_not amplified	TUCR430	79.1	1	79.3
G10	GI-ME-N_amplified	TUCR431	73.2	1	73.1
G9	GI-ME-N_amplified	TUCR431	73.2	1	73.2
E10	GI-ME-N_not amplified	TUCR431	73.2	1	73
E9	GI-ME-N_not amplified	TUCR431	73.2	1	73.1
H10	LAN5_amplified	TUCR431	73.2	1	73.1
H9	LAN5_amplified	TUCR431	73.2	1	73.3
F10	LAN5_not amplified	TUCR431	73.2	1	72.9
F9	LAN5_not amplified	TUCR431	73.2	1	73.2
G11	GI-ME-N_amplified	TUCR432	76	1	75.8
G12	GI-ME-N_amplified	TUCR432	76	1	75.7
E11	GI-ME-N_not amplified	TUCR432	76	1	75.6
E12	GI-ME-N_not amplified	TUCR432	76	1	75.7
H12	LAN5_amplified	TUCR432	76	0	
H11	LAN5_amplified	TUCR432	76	1	71.6
F12	LAN5_not amplified	TUCR432	76	0	
F11	LAN5_not amplified	TUCR432	76	1	74.8
C1	GI-ME-N_amplified	TUCR433	74.2	0	
C2	GI-ME-N_amplified	TUCR433	74.2	0	
A1	GI-ME-N_not amplified	TUCR433	74.2	1	73.5
A2	GI-ME-N_not amplified	TUCR433	74.2	1	73.6
D2	LAN5_amplified	TUCR433	74.2	0	
D1	LAN5_amplified	TUCR433	74.2	1	74.1
B1	LAN5_not amplified	TUCR433	74.2	0	
B2	LAN5_not amplified	TUCR433	74.2	0	
C3	GI-ME-N_amplified	TUCR434	72.8	1	72.9
C4	GI-ME-N_amplified	TUCR434	72.8	1	72.4
A4	GI-ME-N_not amplified	TUCR434	72.8	0	
A3	GI-ME-N_not amplified	TUCR434	72.8	1	72.1
D4	LAN5_amplified	TUCR434	72.8	0	
D3	LAN5_amplified	TUCR434	72.8	1	72.6
B3	LAN5_not amplified	TUCR434	72.8	0	
B4	LAN5_not amplified	TUCR434	72.8	0	
C5	GI-ME-N_amplified	TUCR435	76.4	1	77.2
C6	GI-ME-N_amplified	TUCR435	76.4	1	77.2
A5	GI-ME-N_not amplified	TUCR435	76.4	1	77.6
A6	GI-ME-N_not amplified	TUCR435	76.4	1	77.5
D5	LAN5_amplified	TUCR435	76.4	1	77.1
D6	LAN5_amplified	TUCR435	76.4	1	77
B5	LAN5_not amplified	TUCR435	76.4	1	77.4
B6	LAN5_not amplified	TUCR435	76.4	1	77.3
C7	GI-ME-N_amplified	TUCR436	78.4	1	78.7
C8	GI-ME-N_amplified	TUCR436	78.4	1	78.8
A7	GI-ME-N_not amplified	TUCR436	78.4	1	78.9
A8	GI-ME-N_not amplified	TUCR436	78.4	1	78.7
D7	LAN5_amplified	TUCR436	78.4	1	78.7
D8	LAN5_amplified	TUCR436	78.4	1	78.7
B7	LAN5_not amplified	TUCR436	76.4	1	77.2
B8	LAN5_not amplified	TUCR436	78.4	1	78.9
C10	GI-ME-N_amplified	TUCR437	75.7	1	76.1
C9	GI-ME-N_amplified	TUCR437	75.7	1	75.8
A10	GI-ME-N_not amplified	TUCR437	75.7	1	76.4
A9	GI-ME-N_not amplified	TUCR437	75.7	1	76.6
D10	LAN5_amplified	TUCR437	75.7	1	76
D9	LAN5_amplified	TUCR437	75.7	1	76.1
B10	LAN5_not amplified	TUCR437	75.7	1	76.1
B9	LAN5_not amplified	TUCR437	75.7	1	76.4

C11	GI-ME-N_amplified	TUCR438	77.9	1	76.8
C12	GI-ME-N_amplified	TUCR438	77.9	1	77
A11	GI-ME-N_not amplified	TUCR438	77.9	1	77.2
A12	GI-ME-N_not amplified	TUCR438	77.9	1	77.3
D11	LAN5_amplified	TUCR438	77.9	1	76.8
D12	LAN5_amplified	TUCR438	77.9	1	76.7
B11	LAN5_not amplified	TUCR438	77.9	1	76.9
B12	LAN5_not amplified	TUCR438	77.9	1	77.2
G1	GI-ME-N_amplified	TUCR439	76.5	1	76.4
G2	GI-ME-N_amplified	TUCR439	76.5	1	76.2
E1	GI-ME-N_not amplified	TUCR439	76.5	1	76.2
E2	GI-ME-N_not amplified	TUCR439	76.5	1	75.9
F1	LAN5_amplified	TUCR439	76.5	1	76.2
F2	LAN5_amplified	TUCR439	76.5	1	76.1
H1	LAN5_not amplified	TUCR439	76.5	1	76.5
H2	LAN5_not amplified	TUCR439	76.5	1	76.4
G1	GI-ME-N_amplified	TUCR44	83.4	1	82.6
G2	GI-ME-N_amplified	TUCR44	83.4	1	82.4
E1	GI-ME-N_not amplified	TUCR44	83.4	1	82.4
E2	GI-ME-N_not amplified	TUCR44	83.4	1	82.6
F1	LAN5_amplified	TUCR44	83.4	1	82.4
F2	LAN5_amplified	TUCR44	83.4	1	82.3
H1	LAN5_not amplified	TUCR44	83.4	1	82.8
H2	LAN5_not amplified	TUCR44	83.4	1	82.6
G3	GI-ME-N_amplified	TUCR440	81.3	1	80.6
G4	GI-ME-N_amplified	TUCR440	81.3	1	80.7
E3	GI-ME-N_not amplified	TUCR440	81.3	1	80.4
E4	GI-ME-N_not amplified	TUCR440	81.3	1	80.4
F3	LAN5_amplified	TUCR440	81.3	1	80.5
F4	LAN5_amplified	TUCR440	81.3	1	80.5
H3	LAN5_not amplified	TUCR440	81.3	1	80.9
H4	LAN5_not amplified	TUCR440	81.3	1	80.9
G5	GI-ME-N_amplified	TUCR441	75.8	1	75.2
G6	GI-ME-N_amplified	TUCR441	75.8	1	75
E5	GI-ME-N_not amplified	TUCR441	75.8	1	75
E6	GI-ME-N_not amplified	TUCR441	75.8	1	74.9
H5	LAN5_amplified	TUCR441	75.8	1	75.3
H6	LAN5_amplified	TUCR441	75.8	1	75.1
F5	LAN5_not amplified	TUCR441	75.8	1	75.1
F6	LAN5_not amplified	TUCR441	75.8	1	75
G7	GI-ME-N_amplified	TUCR442	78.8	1	78.2
G8	GI-ME-N_amplified	TUCR442	78.8	1	78.3
E7	GI-ME-N_not amplified	TUCR442	78.8	1	78.2
E8	GI-ME-N_not amplified	TUCR442	78.8	1	78
H7	LAN5_amplified	TUCR442	78.8	1	78.4
H8	LAN5_amplified	TUCR442	78.8	1	78.5
F7	LAN5_not amplified	TUCR442	78.8	1	78.2
F8	LAN5_not amplified	TUCR442	78.8	1	78.2
G10	GI-ME-N_amplified	TUCR443	80.1	1	79.5
G9	GI-ME-N_amplified	TUCR443	80.1	1	79.7
E10	GI-ME-N_not amplified	TUCR443	80.1	1	79.5
E9	GI-ME-N_not amplified	TUCR443	80.1	1	79.6
H10	LAN5_amplified	TUCR443	80.1	1	79.7
H9	LAN5_amplified	TUCR443	80.1	1	79.9
F10	LAN5_not amplified	TUCR443	80.1	1	79.5
F9	LAN5_not amplified	TUCR443	80.1	1	79.5
G11	GI-ME-N_amplified	TUCR444	75.6	1	76
G12	GI-ME-N_amplified	TUCR444	75.6	1	75.9
E11	GI-ME-N_not amplified	TUCR444	75.6	1	76
E12	GI-ME-N_not amplified	TUCR444	75.6	1	76
H11	LAN5_amplified	TUCR444	75.6	1	76
H12	LAN5_amplified	TUCR444	75.6	1	76.4
F11	LAN5_not amplified	TUCR444	75.6	1	76
F12	LAN5_not amplified	TUCR444	75.6	1	75.9
C1	GI-ME-N_amplified	TUCR445	75.5	1	75.9

C2	GI-ME-N_amplified	TUCR445	75.5	1	75.7
A1	GI-ME-N_not amplified	TUCR445	75.5	1	76.3
A2	GI-ME-N_not amplified	TUCR445	75.5	1	76.1
D1	LAN5_amplified	TUCR445	75.5	1	76
D2	LAN5_amplified	TUCR445	75.5	1	72
B1	LAN5_not amplified	TUCR445	75.5	1	76.1
B2	LAN5_not amplified	TUCR445	75.5	1	75.9
C3	GI-ME-N_amplified	TUCR446	79.2	1	77.7
C4	GI-ME-N_amplified	TUCR446	79.2	1	77.9
A3	GI-ME-N_not amplified	TUCR446	79.2	1	78
A4	GI-ME-N_not amplified	TUCR446	79.2	1	78.2
D3	LAN5_amplified	TUCR446	79.2	1	77.8
D4	LAN5_amplified	TUCR446	79.2	1	77.6
B3	LAN5_not amplified	TUCR446	79.2	1	77.8
B4	LAN5_not amplified	TUCR446	79.2	1	78
C5	GI-ME-N_amplified	TUCR447	80.4	1	79.3
C6	GI-ME-N_amplified	TUCR447	80.4	1	79.2
A5	GI-ME-N_not amplified	TUCR447	80.4	1	79.6
A6	GI-ME-N_not amplified	TUCR447	80.4	1	79.5
D5	LAN5_amplified	TUCR447	80.4	1	79.3
D6	LAN5_amplified	TUCR447	80.4	1	79.2
B5	LAN5_not amplified	TUCR447	80.4	1	79.4
B6	LAN5_not amplified	TUCR447	80.4	1	79.3
C7	GI-ME-N_amplified	TUCR448	77.4	1	77.1
C8	GI-ME-N_amplified	TUCR448	77.4	1	77.2
A7	GI-ME-N_not amplified	TUCR448	77.4	1	77.5
A8	GI-ME-N_not amplified	TUCR448	77.4	1	77.6
D7	LAN5_amplified	TUCR448	77.4	1	77.2
D8	LAN5_amplified	TUCR448	77.4	1	77.1
B7	LAN5_not amplified	TUCR448	77.4	1	77.2
B8	LAN5_not amplified	TUCR448	77.4	1	77.3
C10	GI-ME-N_amplified	TUCR449	79.1	1	79.4
C9	GI-ME-N_amplified	TUCR449	79.1	1	79.6
A10	GI-ME-N_not amplified	TUCR449	79.1	1	79.8
A9	GI-ME-N_not amplified	TUCR449	79.1	1	80
D10	LAN5_amplified	TUCR449	79.1	1	79.4
D9	LAN5_amplified	TUCR449	79.1	1	79.5
B10	LAN5_not amplified	TUCR449	79.1	1	79.6
B9	LAN5_not amplified	TUCR449	79.1	1	79.8
G3	GI-ME-N_amplified	TUCR45	74.9	1	75.6
G4	GI-ME-N_amplified	TUCR45	74.9	1	75.7
E3	GI-ME-N_not amplified	TUCR45	74.9	1	75.4
E4	GI-ME-N_not amplified	TUCR45	74.9	1	75.5
F3	LAN5_amplified	TUCR45	74.9	1	75.4
F4	LAN5_amplified	TUCR45	74.9	1	75.4
H3	LAN5_not amplified	TUCR45	74.9	1	75.9
H4	LAN5_not amplified	TUCR45	74.9	1	75.9
C11	GI-ME-N_amplified	TUCR450	79.2	0	
C12	GI-ME-N_amplified	TUCR450	79.2	1	76
A12	GI-ME-N_not amplified	TUCR450	79.2	0	
A11	GI-ME-N_not amplified	TUCR450	79.2	1	76.5
D11	LAN5_amplified	TUCR450	79.2	0	
D12	LAN5_amplified	TUCR450	79.2	0	
B12	LAN5_not amplified	TUCR450	79.2	0	
B11	LAN5_not amplified	TUCR450	79.2	1	77.8
G1	GI-ME-N_amplified	TUCR451	76	1	77.5
G2	GI-ME-N_amplified	TUCR451	76	1	77.3
E1	GI-ME-N_not amplified	TUCR451	76	1	77.2
E2	GI-ME-N_not amplified	TUCR451	76	1	77
F1	LAN5_amplified	TUCR451	76	1	77.3
F2	LAN5_amplified	TUCR451	76	1	77.2
H1	LAN5_not amplified	TUCR451	76	1	77.7
H2	LAN5_not amplified	TUCR451	76	1	77.5
G3	GI-ME-N_amplified	TUCR452	80.9	1	81.3
G4	GI-ME-N_amplified	TUCR452	80.9	1	81.3

E3	GI-ME-N_not amplified	TUCR452	80.9	1	81
E4	GI-ME-N_not amplified	TUCR452	80.9	1	80.9
F3	LAN5_amplified	TUCR452	80.9	1	81.1
F4	LAN5_amplified	TUCR452	80.9	1	81.1
H3	LAN5_not amplified	TUCR452	80.9	1	81.4
H4	LAN5_not amplified	TUCR452	80.9	1	81.5
G5	GI-ME-N_amplified	TUCR453	79.5	1	78.7
G6	GI-ME-N_amplified	TUCR453	79.5	1	78.5
E5	GI-ME-N_not amplified	TUCR453	79.5	1	78.5
E6	GI-ME-N_not amplified	TUCR453	79.5	1	78.4
H5	LAN5_amplified	TUCR453	79.5	1	78.7
H6	LAN5_amplified	TUCR453	79.5	1	78.5
F5	LAN5_not amplified	TUCR453	79.5	1	78.6
F6	LAN5_not amplified	TUCR453	79.5	1	78.4
G7	GI-ME-N_amplified	TUCR454	83.9	1	83.5
G8	GI-ME-N_amplified	TUCR454	83.9	1	83.5
E7	GI-ME-N_not amplified	TUCR454	83.9	1	83.4
E8	GI-ME-N_not amplified	TUCR454	83.9	1	83.4
H7	LAN5_amplified	TUCR454	83.9	1	83.6
H8	LAN5_amplified	TUCR454	83.9	1	83.7
F7	LAN5_not amplified	TUCR454	83.9	1	83.4
F8	LAN5_not amplified	TUCR454	83.9	1	83.5
G10	GI-ME-N_amplified	TUCR455	81.4	1	79.8
G9	GI-ME-N_amplified	TUCR455	81.4	1	79.9
E10	GI-ME-N_not amplified	TUCR455	81.4	1	79.8
E9	GI-ME-N_not amplified	TUCR455	81.4	1	79.8
H10	LAN5_amplified	TUCR455	81.4	1	80
H9	LAN5_amplified	TUCR455	81.4	1	80.2
F10	LAN5_not amplified	TUCR455	81.4	1	79.7
F9	LAN5_not amplified	TUCR455	81.4	1	79.8
G11	GI-ME-N_amplified	TUCR456	82.7	1	82.8
G12	GI-ME-N_amplified	TUCR456	82.7	1	83
E11	GI-ME-N_not amplified	TUCR456	82.7	1	82.7
E12	GI-ME-N_not amplified	TUCR456	82.7	1	82.9
H11	LAN5_amplified	TUCR456	82.7	1	83.1
H12	LAN5_amplified	TUCR456	82.7	1	83.2
F11	LAN5_not amplified	TUCR456	82.7	1	82.7
F12	LAN5_not amplified	TUCR456	82.7	1	82.9
C1	GI-ME-N_amplified	TUCR457	78	1	77.6
C2	GI-ME-N_amplified	TUCR457	78	1	77.3
A1	GI-ME-N_not amplified	TUCR457	78	1	77.9
A2	GI-ME-N_not amplified	TUCR457	78	1	77.8
D1	LAN5_amplified	TUCR457	78	1	77.8
D2	LAN5_amplified	TUCR457	78	1	77.5
B1	LAN5_not amplified	TUCR457	78	1	77.7
B2	LAN5_not amplified	TUCR457	78	1	77.5
C3	GI-ME-N_amplified	TUCR458	81.6	1	80.4
C4	GI-ME-N_amplified	TUCR458	81.6	1	80.6
A3	GI-ME-N_not amplified	TUCR458	81.6	1	80.7
A4	GI-ME-N_not amplified	TUCR458	81.6	1	80.9
D3	LAN5_amplified	TUCR458	81.6	1	80.5
D4	LAN5_amplified	TUCR458	81.6	1	80.4
B3	LAN5_not amplified	TUCR458	81.6	1	80.5
B4	LAN5_not amplified	TUCR458	81.6	1	80.7
C5	GI-ME-N_amplified	TUCR459	83	1	81.8
C6	GI-ME-N_amplified	TUCR459	83	1	81.7
A5	GI-ME-N_not amplified	TUCR459	83	1	82.1
A6	GI-ME-N_not amplified	TUCR459	83	1	82
D5	LAN5_amplified	TUCR459	83	1	81.8
D6	LAN5_amplified	TUCR459	83	1	81.7
B5	LAN5_not amplified	TUCR459	83	1	81.8
B6	LAN5_not amplified	TUCR459	83	1	81.8
G5	GI-ME-N_amplified	TUCR46	75	1	75.3
G6	GI-ME-N_amplified	TUCR46	75	1	75.1
E5	GI-ME-N_not amplified	TUCR46	75	1	75.4

E6	GI-ME-N_not amplified	TUCR46	75	1	75.2
H5	LAN5_amplified	TUCR46	75	1	75.3
H6	LAN5_amplified	TUCR46	75	1	75.3
F5	LAN5_not amplified	TUCR46	75	1	75.3
F6	LAN5_not amplified	TUCR46	75	1	75.1
C7	GI-ME-N_amplified	TUCR460	75.4	1	75.1
C8	GI-ME-N_amplified	TUCR460	75.4	1	75.3
A7	GI-ME-N_not amplified	TUCR460	75.4	1	75.4
A8	GI-ME-N_not amplified	TUCR460	75.4	1	75.4
D7	LAN5_amplified	TUCR460	75.4	1	75.2
D8	LAN5_amplified	TUCR460	75.4	1	75.1
B7	LAN5_not amplified	TUCR460	75.4	1	75.3
B8	LAN5_not amplified	TUCR460	75.4	1	75.4
C10	GI-ME-N_amplified	TUCR461	78.6	1	78.5
C9	GI-ME-N_amplified	TUCR461	78.6	1	78.6
A10	GI-ME-N_not amplified	TUCR461	78.6	1	78.8
A9	GI-ME-N_not amplified	TUCR461	78.6	1	79
D10	LAN5_amplified	TUCR461	78.6	1	78.5
D9	LAN5_amplified	TUCR461	78.6	1	78.5
B10	LAN5_not amplified	TUCR461	78.6	1	78.6
B9	LAN5_not amplified	TUCR461	78.6	1	78.8
C11	GI-ME-N_amplified	TUCR462	76.3	1	76.9
C12	GI-ME-N_amplified	TUCR462	76.3	1	77
A11	GI-ME-N_not amplified	TUCR462	76.3	1	77.2
A12	GI-ME-N_not amplified	TUCR462	76.3	1	77.3
D11	LAN5_amplified	TUCR462	76.3	1	76.9
D12	LAN5_amplified	TUCR462	76.3	1	77
B11	LAN5_not amplified	TUCR462	76.3	1	77
B12	LAN5_not amplified	TUCR462	76.3	1	77
G1	GI-ME-N_amplified	TUCR463	79.3	1	79.4
G2	GI-ME-N_amplified	TUCR463	79.3	1	79.1
E1	GI-ME-N_not amplified	TUCR463	79.3	1	78.6
E2	GI-ME-N_not amplified	TUCR463	79.3	1	78.8
F1	LAN5_amplified	TUCR463	79.3	1	79.1
F2	LAN5_amplified	TUCR463	79.3	1	79
H1	LAN5_not amplified	TUCR463	79.3	1	79.5
H2	LAN5_not amplified	TUCR463	79.3	1	79.3
G3	GI-ME-N_amplified	TUCR464	79.4	1	80.2
G4	GI-ME-N_amplified	TUCR464	79.4	1	80.2
E3	GI-ME-N_not amplified	TUCR464	79.4	1	80
E4	GI-ME-N_not amplified	TUCR464	79.4	1	80
F4	LAN5_amplified	TUCR464	79.4	0	
F3	LAN5_amplified	TUCR464	79.4	1	80.1
H3	LAN5_not amplified	TUCR464	79.4	1	80.4
H4	LAN5_not amplified	TUCR464	79.4	1	80.4
G5	GI-ME-N_amplified	TUCR465	78	1	79.1
G6	GI-ME-N_amplified	TUCR465	78	1	79
E5	GI-ME-N_not amplified	TUCR465	78	1	78.9
E6	GI-ME-N_not amplified	TUCR465	78	1	78.8
H5	LAN5_amplified	TUCR465	78	1	79.4
H6	LAN5_amplified	TUCR465	78	1	79.2
F5	LAN5_not amplified	TUCR465	78	1	79
F6	LAN5_not amplified	TUCR465	78	1	78.7
G7	GI-ME-N_amplified	TUCR466	77.3	1	76.6
G8	GI-ME-N_amplified	TUCR466	77.3	1	76.7
E7	GI-ME-N_not amplified	TUCR466	77.3	1	76.5
E8	GI-ME-N_not amplified	TUCR466	77.3	1	76.5
H7	LAN5_amplified	TUCR466	77.3	1	76.9
H8	LAN5_amplified	TUCR466	77.3	1	76.9
F7	LAN5_not amplified	TUCR466	77.3	1	76.4
F8	LAN5_not amplified	TUCR466	77.3	1	76.6
G10	GI-ME-N_amplified	TUCR467	81.3	1	80.4
G9	GI-ME-N_amplified	TUCR467	81.3	1	80.6
E10	GI-ME-N_not amplified	TUCR467	81.3	1	80.4
E9	GI-ME-N_not amplified	TUCR467	81.3	1	80.5

H10	LAN5_amplified	TUCR467	81.3	1	80.6
H9	LAN5_amplified	TUCR467	81.3	1	80.8
F10	LAN5_not amplified	TUCR467	81.3	1	80.4
F9	LAN5_not amplified	TUCR467	81.3	1	80.5
G11	GI-ME-N_amplified	TUCR468	80.6	1	79.4
G12	GI-ME-N_amplified	TUCR468	80.6	1	79.5
E11	GI-ME-N_not amplified	TUCR468	80.6	1	79.3
E12	GI-ME-N_not amplified	TUCR468	80.6	1	79.4
H11	LAN5_amplified	TUCR468	80.6	1	79.5
H12	LAN5_amplified	TUCR468	80.6	1	79.7
F11	LAN5_not amplified	TUCR468	80.6	1	79.2
F12	LAN5_not amplified	TUCR468	80.6	1	79.4
C1	GI-ME-N_amplified	TUCR469	81	1	79.9
C2	GI-ME-N_amplified	TUCR469	81	1	79.6
A1	GI-ME-N_not amplified	TUCR469	81	1	80.1
A2	GI-ME-N_not amplified	TUCR469	81	1	79.9
D1	LAN5_amplified	TUCR469	81	1	80
D2	LAN5_amplified	TUCR469	81	1	79.5
B1	LAN5_not amplified	TUCR469	81	1	79.9
B2	LAN5_not amplified	TUCR469	81	1	79.7
G7	GI-ME-N_amplified	TUCR47	81.5	1	77.5
G8	GI-ME-N_amplified	TUCR47	81.5	1	78
E7	GI-ME-N_not amplified	TUCR47	78.4	1	78.2
E8	GI-ME-N_not amplified	TUCR47	81.5	1	78.2
H8	LAN5_amplified	TUCR47	81.5	0	
H7	LAN5_amplified	TUCR47	81.5	1	78
F7	LAN5_not amplified	TUCR47	81.5	1	78.2
F8	LAN5_not amplified	TUCR47	81.5	1	74.2
C3	GI-ME-N_amplified	TUCR470	80	1	78.8
C4	GI-ME-N_amplified	TUCR470	80	1	79
A3	GI-ME-N_not amplified	TUCR470	80	1	79.3
A4	GI-ME-N_not amplified	TUCR470	80	1	79.4
D3	LAN5_amplified	TUCR470	80	1	79.2
D4	LAN5_amplified	TUCR470	80	2	76.7, 79.1
B4	LAN5_not amplified	TUCR470	80	0	
B3	LAN5_not amplified	TUCR470	80	1	79
C5	GI-ME-N_amplified	TUCR471	78.8	1	78
C6	GI-ME-N_amplified	TUCR471	78.8	1	77.9
A5	GI-ME-N_not amplified	TUCR471	78.8	1	78.3
A6	GI-ME-N_not amplified	TUCR471	78.8	1	78.2
D5	LAN5_amplified	TUCR471	78.8	1	78
D6	LAN5_amplified	TUCR471	78.8	1	77.9
B5	LAN5_not amplified	TUCR471	78.8	1	78.1
B6	LAN5_not amplified	TUCR471	78.8	1	77.9
C7	GI-ME-N_amplified	TUCR472	75.4	1	76.2
C8	GI-ME-N_amplified	TUCR472	75.4	1	76.3
A7	GI-ME-N_not amplified	TUCR472	75.4	1	76.5
A8	GI-ME-N_not amplified	TUCR472	75.4	1	76.7
D7	LAN5_amplified	TUCR472	75.4	1	76.3
D8	LAN5_amplified	TUCR472	75.4	1	76.3
B7	LAN5_not amplified	TUCR472	75.4	1	76.3
B8	LAN5_not amplified	TUCR472	75.4	1	76.4
C10	GI-ME-N_amplified	TUCR473	84.8	1	83.6
C9	GI-ME-N_amplified	TUCR473	84.8	1	83.7
A10	GI-ME-N_not amplified	TUCR473	84.8	1	84
A9	GI-ME-N_not amplified	TUCR473	84.8	1	84.2
D10	LAN5_amplified	TUCR473	84.8	1	83.5
D9	LAN5_amplified	TUCR473	84.8	1	83.6
B10	LAN5_not amplified	TUCR473	84.8	1	83.7
B9	LAN5_not amplified	TUCR473	84.8	1	83.8
C11	GI-ME-N_amplified	TUCR474	83.7	1	82.1
C12	GI-ME-N_amplified	TUCR474	83.7	1	82.3
A11	GI-ME-N_not amplified	TUCR474	83.7	1	82.7
A12	GI-ME-N_not amplified	TUCR474	83.7	1	82.7
D11	LAN5_amplified	TUCR474	83.7	1	82.1

D12	LAN5_amplified	TUCR474	83.7	1	82.3
B11	LAN5_not amplified	TUCR474	83.7	1	82.3
B12	LAN5_not amplified	TUCR474	83.7	1	82.5
G1	GI-ME-N_amplified	TUCR475	80.2	1	80.2
G2	GI-ME-N_amplified	TUCR475	80.2	1	80
E1	GI-ME-N_not amplified	TUCR475	80.2	1	80
E2	GI-ME-N_not amplified	TUCR475	80.2	1	79.8
F1	LAN5_amplified	TUCR475	80.2	1	80
F2	LAN5_amplified	TUCR475	80.2	1	79.9
H1	LAN5_not amplified	TUCR475	80.2	1	80.5
H2	LAN5_not amplified	TUCR475	80.2	1	80.2
G3	GI-ME-N_amplified	TUCR476	81	1	76.5
G4	GI-ME-N_amplified	TUCR476	81	1	74.9
E3	GI-ME-N_not amplified	TUCR476	81	1	74.8
E4	GI-ME-N_not amplified	TUCR476	81	1	73.8
F3	LAN5_amplified	TUCR476	81	1	74.7
F4	LAN5_amplified	TUCR476	81	2	76.1, 80.7
H3	LAN5_not amplified	TUCR476	81	1	74.9
H4	LAN5_not amplified	TUCR476	81	1	75
G5	GI-ME-N_amplified	TUCR477	88.3	1	86.7
G6	GI-ME-N_amplified	TUCR477	88.3	1	86.5
E5	GI-ME-N_not amplified	TUCR477	88.3	1	86.5
E6	GI-ME-N_not amplified	TUCR477	88.3	1	86.4
H5	LAN5_amplified	TUCR477	88.3	1	86.9
H6	LAN5_amplified	TUCR477	88.3	1	86.7
F5	LAN5_not amplified	TUCR477	88.3	1	86.6
F6	LAN5_not amplified	TUCR477	88.3	1	86.4
G7	GI-ME-N_amplified	TUCR478	78.7	1	78.9
G8	GI-ME-N_amplified	TUCR478	78.7	1	79
E7	GI-ME-N_not amplified	TUCR478	78.7	1	78.8
E8	GI-ME-N_not amplified	TUCR478	78.7	1	78.9
H7	LAN5_amplified	TUCR478	78.7	1	79.1
H8	LAN5_amplified	TUCR478	78.7	1	79.1
F7	LAN5_not amplified	TUCR478	78.7	1	78.8
F8	LAN5_not amplified	TUCR478	78.7	1	78.9
G10	GI-ME-N_amplified	TUCR479	79.2	1	79.2
G9	GI-ME-N_amplified	TUCR479	79.2	1	79.4
E10	GI-ME-N_not amplified	TUCR479	79.2	1	80.7
E9	GI-ME-N_not amplified	TUCR479	79.2	2	78.4, 80.6
H10	LAN5_amplified	TUCR479	79.2	1	78.9
H9	LAN5_amplified	TUCR479	79.2	1	79.1
F10	LAN5_not amplified	TUCR479	79.2	1	78.7
F9	LAN5_not amplified	TUCR479	79.2	1	78.8
G10	GI-ME-N_amplified	TUCR48	81.5	1	82
G9	GI-ME-N_amplified	TUCR48	81.5	1	82.1
E10	GI-ME-N_not amplified	TUCR48	81.5	1	81.8
E9	GI-ME-N_not amplified	TUCR48	81.5	1	82
H10	LAN5_amplified	TUCR48	81.5	1	82.1
H9	LAN5_amplified	TUCR48	81.5	1	82.3
F10	LAN5_not amplified	TUCR48	81.5	1	81.8
F9	LAN5_not amplified	TUCR48	81.5	1	82
G11	GI-ME-N_amplified	TUCR480	71.6	1	71.1
G12	GI-ME-N_amplified	TUCR480	71.6	1	71.2
E11	GI-ME-N_not amplified	TUCR480	71.6	1	70.8
E12	GI-ME-N_not amplified	TUCR480	71.6	1	71
H11	LAN5_amplified	TUCR480	71.6	1	71.4
H12	LAN5_amplified	TUCR480	71.6	1	71.5
F11	LAN5_not amplified	TUCR480	71.6	1	70.9
F12	LAN5_not amplified	TUCR480	71.6	1	71.2
C1	GI-ME-N_amplified	TUCR481	75.9	1	75.2
C2	GI-ME-N_amplified	TUCR481	75.9	1	75
A1	GI-ME-N_not amplified	TUCR481	75.9	1	75.6
A2	GI-ME-N_not amplified	TUCR481	75.9	1	75.3
D1	LAN5_amplified	TUCR481	75.9	1	75.2
D2	LAN5_amplified	TUCR481	75.9	1	75

B1	LAN5_not amplified	TUCR481	75.9	1	75.2
B2	LAN5_not amplified	TUCR481	75.9	1	75.1
C3	GI-ME-N_amplified	TUCR482	77.6	0	
C4	GI-ME-N_amplified	TUCR482	77.6	1	80.2
A3	GI-ME-N_not amplified	TUCR482	77.6	0	
A4	GI-ME-N_not amplified	TUCR482	77.6	0	
D3	LAN5_amplified	TUCR482	77.6	0	
D4	LAN5_amplified	TUCR482	77.6	0	
B3	LAN5_not amplified	TUCR482	77.6	0	
B4	LAN5_not amplified	TUCR482	77.6	0	
C5	GI-ME-N_amplified	TUCR483	75.6	1	75.8
C6	GI-ME-N_amplified	TUCR483	75.6	1	75.6
A5	GI-ME-N_not amplified	TUCR483	75.6	1	75.9
A6	GI-ME-N_not amplified	TUCR483	75.6	1	75.9
D5	LAN5_amplified	TUCR483	75.6	1	75.8
D6	LAN5_amplified	TUCR483	75.6	1	75.6
B5	LAN5_not amplified	TUCR483	75.6	1	75.8
B6	LAN5_not amplified	TUCR483	75.6	1	75.7
G11	GI-ME-N_amplified	TUCR49	81.5	1	80.8
G12	GI-ME-N_amplified	TUCR49	81.5	1	81
E11	GI-ME-N_not amplified	TUCR49	81.5	1	81
E12	GI-ME-N_not amplified	TUCR49	81.5	1	80.9
H11	LAN5_amplified	TUCR49	81.5	1	81.1
H12	LAN5_amplified	TUCR49	81.5	1	81.2
F11	LAN5_not amplified	TUCR49	81.5	1	80.8
F12	LAN5_not amplified	TUCR49	81.5	1	80.9
C11	GI-ME-N_amplified	TUCR5	80.2	1	78.9
C12	GI-ME-N_amplified	TUCR5	80.2	1	79.1
A11	GI-ME-N_not amplified	TUCR5	80.2	1	79.3
A12	GI-ME-N_not amplified	TUCR5	80.2	1	79.4
D12	LAN5_amplified	TUCR5	80.2	0	
D11	LAN5_amplified	TUCR5	80.2	1	78.9
B11	LAN5_not amplified	TUCR5	80.2	1	79.1
B12	LAN5_not amplified	TUCR5	80.2	1	79.3
C1	GI-ME-N_amplified	TUCR50	78.5	1	79.9
C2	GI-ME-N_amplified	TUCR50	78.5	1	79.8
A1	GI-ME-N_not amplified	TUCR50	78.5	1	80.2
A2	GI-ME-N_not amplified	TUCR50	78.5	1	80.1
D1	LAN5_amplified	TUCR50	78.5	1	80.2
D2	LAN5_amplified	TUCR50	78.5	1	80.1
B1	LAN5_not amplified	TUCR50	78.5	1	80
B2	LAN5_not amplified	TUCR50	78.5	1	79.8
C3	GI-ME-N_amplified	TUCR51	78.9	1	77.5
C4	GI-ME-N_amplified	TUCR51	78.9	1	77.7
A3	GI-ME-N_not amplified	TUCR51	78.9	1	77.9
A4	GI-ME-N_not amplified	TUCR51	78.9	1	78
D4	LAN5_amplified	TUCR51	78.9	0	
D3	LAN5_amplified	TUCR51	78.9	1	77.6
B4	LAN5_not amplified	TUCR51	78.9	0	
B3	LAN5_not amplified	TUCR51	78.9	1	77.5
C5	GI-ME-N_amplified	TUCR52	78.4	1	77.7
C6	GI-ME-N_amplified	TUCR52	78.4	1	77.6
A5	GI-ME-N_not amplified	TUCR52	78.4	1	78
A6	GI-ME-N_not amplified	TUCR52	78.4	1	77.9
D5	LAN5_amplified	TUCR52	78.4	1	77.7
D6	LAN5_amplified	TUCR52	78.4	1	77.4
B5	LAN5_not amplified	TUCR52	78.4	1	78
B6	LAN5_not amplified	TUCR52	78.4	1	77.8
C7	GI-ME-N_amplified	TUCR53	77.6	1	76.9
C8	GI-ME-N_amplified	TUCR53	77.6	1	77
A7	GI-ME-N_not amplified	TUCR53	77.6	1	77.2
A8	GI-ME-N_not amplified	TUCR53	77.6	1	77.3
D7	LAN5_amplified	TUCR53	77.6	1	77
D8	LAN5_amplified	TUCR53	77.6	1	76.7
B7	LAN5_not amplified	TUCR53	77.6	1	76.9

B8	LAN5_not amplified	TUCR53	77.6	1	77.1
C10	GI-ME-N_amplified	TUCR54	81.4	1	76.8
C9	GI-ME-N_amplified	TUCR54	81.4	1	81.5
A10	GI-ME-N_not amplified	TUCR54	81.4	1	81.7
A9	GI-ME-N_not amplified	TUCR54	81.4	1	81.9
D10	LAN5_amplified	TUCR54	81.4	0	
D9	LAN5_amplified	TUCR54	81.4	0	
B10	LAN5_not amplified	TUCR54	81.4	0	
B9	LAN5_not amplified	TUCR54	81.4	0	
C11	GI-ME-N_amplified	TUCR55	73.2	1	74
C12	GI-ME-N_amplified	TUCR55	73.2	1	74.3
A11	GI-ME-N_not amplified	TUCR55	73.2	1	73.7
A12	GI-ME-N_not amplified	TUCR55	73.2	1	73.4
D11	LAN5_amplified	TUCR55	73.2	1	74.2
D12	LAN5_amplified	TUCR55	73.2	1	74.7
B12	LAN5_not amplified	TUCR55	73.2	0	
B11	LAN5_not amplified	TUCR55	73.2	1	74.3
G1	GI-ME-N_amplified	TUCR56	80.3	1	80.1
G2	GI-ME-N_amplified	TUCR56	80.3	1	79.9
E1	GI-ME-N_not amplified	TUCR56	80.3	1	79.8
E2	GI-ME-N_not amplified	TUCR56	80.3	1	79.8
F1	LAN5_amplified	TUCR56	80.3	0	
F2	LAN5_amplified	TUCR56	80.3	0	
H1	LAN5_not amplified	TUCR56	80.3	0	
H2	LAN5_not amplified	TUCR56	80.3	0	
G3	GI-ME-N_amplified	TUCR57	79.6	1	79.9
G4	GI-ME-N_amplified	TUCR57	79.6	1	80
E3	GI-ME-N_not amplified	TUCR57	79.6	1	76.1
E4	GI-ME-N_not amplified	TUCR57	79.6	1	79.6
F3	LAN5_amplified	TUCR57	79.6	1	76.2
F4	LAN5_amplified	TUCR57	79.6	1	76.3
H3	LAN5_not amplified	TUCR57	79.6	1	80.7
H4	LAN5_not amplified	TUCR57	79.6	1	80.6
G5	GI-ME-N_amplified	TUCR58	78.5	1	77.8
G6	GI-ME-N_amplified	TUCR58	78.5	1	77.6
E5	GI-ME-N_not amplified	TUCR58	78.5	1	77.6
E6	GI-ME-N_not amplified	TUCR58	78.5	1	77.6
H6	LAN5_amplified	TUCR58	78.5	0	
H5	LAN5_amplified	TUCR58	78.5	1	77.7
F5	LAN5_not amplified	TUCR58	78.5	0	
F6	LAN5_not amplified	TUCR58	78.5	0	
G7	GI-ME-N_amplified	TUCR59	77.3	1	78
G8	GI-ME-N_amplified	TUCR59	77.3	1	78
E8	GI-ME-N_not amplified	TUCR59	77.3	0	
E7	GI-ME-N_not amplified	TUCR59	77.3	1	77.7
H8	LAN5_amplified	TUCR59	77.3	0	
H7	LAN5_amplified	TUCR59	77.3	1	77.6
F7	LAN5_not amplified	TUCR59	77.3	0	
F8	LAN5_not amplified	TUCR59	77.3	0	
G1	GI-ME-N_amplified	TUCR6	80.3	1	80.5
G2	GI-ME-N_amplified	TUCR6	80.3	1	80.4
E1	GI-ME-N_not amplified	TUCR6	80.3	1	80.4
E2	GI-ME-N_not amplified	TUCR6	80.3	1	80.1
F1	LAN5_amplified	TUCR6	80.3	1	80.4
F2	LAN5_amplified	TUCR6	80.3	1	80.2
H1	LAN5_not amplified	TUCR6	80.3	1	80.7
H2	LAN5_not amplified	TUCR6	80.3	1	80.5
G10	GI-ME-N_amplified	TUCR60	76	1	76.2
G9	GI-ME-N_amplified	TUCR60	76	1	76.4
E9	GI-ME-N_not amplified	TUCR60	76	0	
E10	GI-ME-N_not amplified	TUCR60	76	1	79.8
H10	LAN5_amplified	TUCR60	76	0	
H9	LAN5_amplified	TUCR60	76	0	
F10	LAN5_not amplified	TUCR60	76	0	
F9	LAN5_not amplified	TUCR60	76	0	

G11	GI-ME-N_amplified	TUCR61	83.7	1	83.4
G12	GI-ME-N_amplified	TUCR61	83.7	1	83.6
E11	GI-ME-N_not amplified	TUCR61	83.7	1	83.3
E12	GI-ME-N_not amplified	TUCR61	83.7	1	83.5
H11	LAN5_amplified	TUCR61	83.7	1	83.6
H12	LAN5_amplified	TUCR61	83.7	1	83.8
F11	LAN5_not amplified	TUCR61	83.7	1	83.2
F12	LAN5_not amplified	TUCR61	83.7	1	83.4
C1	GI-ME-N_amplified	TUCR62	86.6	1	84
C2	GI-ME-N_amplified	TUCR62	86.6	1	83.8
A1	GI-ME-N_not amplified	TUCR62	86.6	1	84.3
A2	GI-ME-N_not amplified	TUCR62	86.6	1	84.2
D1	LAN5_amplified	TUCR62	86.6	1	84.2
D2	LAN5_amplified	TUCR62	86.6	1	84
B1	LAN5_not amplified	TUCR62	86.6	1	84.1
B2	LAN5_not amplified	TUCR62	86.6	1	83.9
C3	GI-ME-N_amplified	TUCR63	77.1	1	76.2
C4	GI-ME-N_amplified	TUCR63	77.1	1	76.4
A3	GI-ME-N_not amplified	TUCR63	77.1	1	76.4
A4	GI-ME-N_not amplified	TUCR63	77.1	1	76.8
D3	LAN5_amplified	TUCR63	77.1	1	76.6
D4	LAN5_amplified	TUCR63	77.1	1	76.5
B3	LAN5_not amplified	TUCR63	77.1	1	76.4
B4	LAN5_not amplified	TUCR63	77.1	1	76.6
C5	GI-ME-N_amplified	TUCR64	80.3	1	79.2
C6	GI-ME-N_amplified	TUCR64	80.3	1	79.1
A5	GI-ME-N_not amplified	TUCR64	80.3	1	79.5
A6	GI-ME-N_not amplified	TUCR64	80.3	1	79.4
D5	LAN5_amplified	TUCR64	80.3	1	79.2
D6	LAN5_amplified	TUCR64	80.3	1	79.1
B5	LAN5_not amplified	TUCR64	80.3	1	79.3
B6	LAN5_not amplified	TUCR64	80.3	1	79.2
C8	GI-ME-N_amplified	TUCR65	75.3	1	74.3
C7	GI-ME-N_amplified	TUCR65	75.3	2	70.5, 74.3
A7	GI-ME-N_not amplified	TUCR65	75.3	1	74.6
A8	GI-ME-N_not amplified	TUCR65	75.3	1	74.8
D7	LAN5_amplified	TUCR65	75.3	2	70.9, 74.1
D8	LAN5_amplified	TUCR65	75.3	2	70.9, 73.9
B7	LAN5_not amplified	TUCR65	75.3	2	71.2, 74.3
B8	LAN5_not amplified	TUCR65	75.3	2	70.9, 74.4
C10	GI-ME-N_amplified	TUCR66	76.9	1	76.8
C9	GI-ME-N_amplified	TUCR66	76.9	1	76.8
A10	GI-ME-N_not amplified	TUCR66	76.9	1	77.2
A9	GI-ME-N_not amplified	TUCR66	76.9	1	77.4
D10	LAN5_amplified	TUCR66	76.9	0	
D9	LAN5_amplified	TUCR66	76.9	1	77
B10	LAN5_not amplified	TUCR66	76.9	1	76.9
B9	LAN5_not amplified	TUCR66	76.9	1	77.2
C11	GI-ME-N_amplified	TUCR67	80.7	1	79.7
C12	GI-ME-N_amplified	TUCR67	80.7	1	79.7
A11	GI-ME-N_not amplified	TUCR67	80.7	1	80
A12	GI-ME-N_not amplified	TUCR67	80.7	1	80.1
D12	LAN5_amplified	TUCR67	80.7	0	
D11	LAN5_amplified	TUCR67	80.7	1	79.5
B11	LAN5_not amplified	TUCR67	80.7	1	79.8
B12	LAN5_not amplified	TUCR67	80.7	1	79.9
G1	GI-ME-N_amplified	TUCR68	75.9	1	77.3
G2	GI-ME-N_amplified	TUCR68	75.9	1	77
E1	GI-ME-N_not amplified	TUCR68	75.9	1	77
E2	GI-ME-N_not amplified	TUCR68	75.9	1	77
F1	LAN5_amplified	TUCR68	75.9	1	75.8
F2	LAN5_amplified	TUCR68	75.9	1	75.7
H1	LAN5_not amplified	TUCR68	75.9	1	73.4
H2	LAN5_not amplified	TUCR68	75.9	1	73.3
G3	GI-ME-N_amplified	TUCR69	77.5	1	77.4

G4	GI-ME-N_amplified	TUCR69	77.5	1	77.5
E3	GI-ME-N_not amplified	TUCR69	77.5	1	77.2
E4	GI-ME-N_not amplified	TUCR69	77.5	1	77.3
F3	LAN5_amplified	TUCR69	77.5	1	77.6
F4	LAN5_amplified	TUCR69	77.5	1	77.5
H3	LAN5_not amplified	TUCR69	77.5	0	
H4	LAN5_not amplified	TUCR69	77.5	0	
G3	GI-ME-N_amplified	TUCR7	77.8	1	77.2
G4	GI-ME-N_amplified	TUCR7	77.8	1	77.3
E3	GI-ME-N_not amplified	TUCR7	77.8	1	76.9
E4	GI-ME-N_not amplified	TUCR7	77.8	1	76.9
F3	LAN5_amplified	TUCR7	77.8	1	77
F4	LAN5_amplified	TUCR7	77.8	1	77
H3	LAN5_not amplified	TUCR7	77.8	1	77.4
H4	LAN5_not amplified	TUCR7	77.8	1	77.4
G5	GI-ME-N_amplified	TUCR70	79.7	1	79.5
G6	GI-ME-N_amplified	TUCR70	79.7	1	79.3
E5	GI-ME-N_not amplified	TUCR70	79.7	1	79.6
E6	GI-ME-N_not amplified	TUCR70	79.7	1	79.2
H5	LAN5_amplified	TUCR70	79.7	1	79.6
H6	LAN5_amplified	TUCR70	79.7	1	79.4
F6	LAN5_not amplified	TUCR70	79.7	0	
F5	LAN5_not amplified	TUCR70	79.7	1	79.3
G7	GI-ME-N_amplified	TUCR71	76.4	1	76
G8	GI-ME-N_amplified	TUCR71	76.4	1	76
E7	GI-ME-N_not amplified	TUCR71	76.4	1	75.8
E8	GI-ME-N_not amplified	TUCR71	76.4	1	75.9
H7	LAN5_amplified	TUCR71	76.4	1	76.2
H8	LAN5_amplified	TUCR71	76.4	1	76
F7	LAN5_not amplified	TUCR71	76.4	1	75.8
F8	LAN5_not amplified	TUCR71	76.4	1	75.9
G10	GI-ME-N_amplified	TUCR72	83.8	1	83.4
G9	GI-ME-N_amplified	TUCR72	83.8	1	83.6
E10	GI-ME-N_not amplified	TUCR72	83.8	1	83.3
E9	GI-ME-N_not amplified	TUCR72	83.8	1	83.4
H10	LAN5_amplified	TUCR72	83.8	1	83.7
H9	LAN5_amplified	TUCR72	83.8	1	83.8
F10	LAN5_not amplified	TUCR72	83.8	1	83.2
F9	LAN5_not amplified	TUCR72	83.8	1	83.5
G11	GI-ME-N_amplified	TUCR73	83.3	1	82
G12	GI-ME-N_amplified	TUCR73	83.3	1	82.2
E11	GI-ME-N_not amplified	TUCR73	83.3	1	81.8
E12	GI-ME-N_not amplified	TUCR73	83.3	1	82
H11	LAN5_amplified	TUCR73	83.3	1	82.2
H12	LAN5_amplified	TUCR73	83.3	1	82.4
F11	LAN5_not amplified	TUCR73	83.3	1	81.7
F12	LAN5_not amplified	TUCR73	83.3	1	82
C1	GI-ME-N_amplified	TUCR74	78.4	1	77.8
C2	GI-ME-N_amplified	TUCR74	78.4	1	77.5
A1	GI-ME-N_not amplified	TUCR74	78.4	1	78
A2	GI-ME-N_not amplified	TUCR74	78.4	1	77.9
D1	LAN5_amplified	TUCR74	78.4	1	77.9
D2	LAN5_amplified	TUCR74	78.4	1	77.7
B1	LAN5_not amplified	TUCR74	78.4	1	77.8
B2	LAN5_not amplified	TUCR74	78.4	1	77.6
C3	GI-ME-N_amplified	TUCR75	76.4	1	75.1
C4	GI-ME-N_amplified	TUCR75	76.4	1	75.3
A3	GI-ME-N_not amplified	TUCR75	76.4	1	75.5
A4	GI-ME-N_not amplified	TUCR75	76.4	1	75.7
D4	LAN5_amplified	TUCR75	76.4	0	
D3	LAN5_amplified	TUCR75	76.4	1	75.2
B3	LAN5_not amplified	TUCR75	76.4	1	75.4
B4	LAN5_not amplified	TUCR75	76.4	1	75.5
C5	GI-ME-N_amplified	TUCR76	73.3	1	74.4
C6	GI-ME-N_amplified	TUCR76	73.3	1	74.3

A5	GI-ME-N_not amplified	TUCR76	73.3	1	74.8
A6	GI-ME-N_not amplified	TUCR76	73.3	1	74.7
D5	LAN5_amplified	TUCR76	73.3	1	74.4
D6	LAN5_amplified	TUCR76	73.3	1	74.4
B5	LAN5_not amplified	TUCR76	73.3	1	74.7
B6	LAN5_not amplified	TUCR76	73.3	1	74.5
C7	GI-ME-N_amplified	TUCR77	78.8	1	78.5
C8	GI-ME-N_amplified	TUCR77	78.8	1	78.6
A7	GI-ME-N_not amplified	TUCR77	78.8	1	78.7
A8	GI-ME-N_not amplified	TUCR77	78.8	1	78.8
D7	LAN5_amplified	TUCR77	78.8	1	78.5
D8	LAN5_amplified	TUCR77	78.8	1	78.5
B7	LAN5_not amplified	TUCR77	78.8	1	78.6
B8	LAN5_not amplified	TUCR77	78.8	1	78.6
C10	GI-ME-N_amplified	TUCR78	82.5	1	81.6
C9	GI-ME-N_amplified	TUCR78	82.5	1	81.4
A10	GI-ME-N_not amplified	TUCR78	82.5	1	82
A9	GI-ME-N_not amplified	TUCR78	82.5	1	81.9
D10	LAN5_amplified	TUCR78	82.5	1	81.6
D9	LAN5_amplified	TUCR78	82.5	1	81.4
B10	LAN5_not amplified	TUCR78	82.5	1	81.8
B9	LAN5_not amplified	TUCR78	82.5	1	81.6
C11	GI-ME-N_amplified	TUCR79	74.2	1	74
C12	GI-ME-N_amplified	TUCR79	74.2	1	74.3
A11	GI-ME-N_not amplified	TUCR79	74.2	1	74.6
A12	GI-ME-N_not amplified	TUCR79	74.2	1	74.7
D11	LAN5_amplified	TUCR79	74.2	1	74
D12	LAN5_amplified	TUCR79	74.2	1	74.3
B11	LAN5_not amplified	TUCR79	74.2	1	74.4
B12	LAN5_not amplified	TUCR79	74.2	1	74.6
G5	GI-ME-N_amplified	TUCR8	78.8	1	77.8
G6	GI-ME-N_amplified	TUCR8	78.8	1	77.6
E5	GI-ME-N_not amplified	TUCR8	78.8	1	77.6
E6	GI-ME-N_not amplified	TUCR8	78.8	1	77.5
H5	LAN5_amplified	TUCR8	78.8	1	78.1
H6	LAN5_amplified	TUCR8	78.8	1	77.9
F5	LAN5_not amplified	TUCR8	78.8	1	77.6
F6	LAN5_not amplified	TUCR8	78.8	1	77.5
G1	GI-ME-N_amplified	TUCR80	74.2	1	75.4
G2	GI-ME-N_amplified	TUCR80	74.2	1	75.3
E1	GI-ME-N_not amplified	TUCR80	74.2	1	75.2
E2	GI-ME-N_not amplified	TUCR80	74.2	1	75
F1	LAN5_amplified	TUCR80	74.2	1	75.2
F2	LAN5_amplified	TUCR80	74.2	1	75.1
H1	LAN5_not amplified	TUCR80	74.2	1	75.6
H2	LAN5_not amplified	TUCR80	74.2	1	75.3
G3	GI-ME-N_amplified	TUCR81	74.3	1	74
G4	GI-ME-N_amplified	TUCR81	74.3	1	74.2
E3	GI-ME-N_not amplified	TUCR81	74.3	1	73.9
E4	GI-ME-N_not amplified	TUCR81	74.3	1	73.9
F3	LAN5_amplified	TUCR81	74.3	1	74
F4	LAN5_amplified	TUCR81	74.3	1	74
H3	LAN5_not amplified	TUCR81	74.3	1	74.2
H4	LAN5_not amplified	TUCR81	74.3	1	74.3
G6	GI-ME-N_amplified	TUCR82	74.9	10	
G5	GI-ME-N_amplified	TUCR82	74.9	11	
E5	GI-ME-N_not amplified	TUCR82	74.9	2	63.7, 65.6
E6	GI-ME-N_not amplified	TUCR82	74.9	2	61.4, 62.6
H5	LAN5_amplified	TUCR82	74.9	8	
H6	LAN5_amplified	TUCR82	74.9	11	
F5	LAN5_not amplified	TUCR82	74.9	1	61.5
F6	LAN5_not amplified	TUCR82	74.9	4	
G7	GI-ME-N_amplified	TUCR83	79.1	1	77.8
G8	GI-ME-N_amplified	TUCR83	79.1	1	77.8
E7	GI-ME-N_not amplified	TUCR83	79.1	1	77.8

E8	GI-ME-N_not amplified	TUCR83	79.1	1	77.7
H7	LAN5_amplified	TUCR83	79.1	0	
H8	LAN5_amplified	TUCR83	79.1	1	79.3
F8	LAN5_not amplified	TUCR83	79.1	0	
F7	LAN5_not amplified	TUCR83	79.1	1	74.7
G10	GI-ME-N_amplified	TUCR84	77.2	1	76.6
G9	GI-ME-N_amplified	TUCR84	77.2	1	76.8
E10	GI-ME-N_not amplified	TUCR84	77.2	1	76.8
E9	GI-ME-N_not amplified	TUCR84	77.2	1	76.9
H9	LAN5_amplified	TUCR84	77.2	0	
H10	LAN5_amplified	TUCR84	77.2	1	74.5
F10	LAN5_not amplified	TUCR84	77.2	0	
F9	LAN5_not amplified	TUCR84	77.2	0	
G11	GI-ME-N_amplified	TUCR85	79.6	1	79.1
G12	GI-ME-N_amplified	TUCR85	79.6	1	79.2
E11	GI-ME-N_not amplified	TUCR85	79.6	1	79.1
E12	GI-ME-N_not amplified	TUCR85	79.6	1	79.2
H11	LAN5_amplified	TUCR85	79.6	1	78.7
H12	LAN5_amplified	TUCR85	79.6	1	79.2
F11	LAN5_not amplified	TUCR85	79.6	1	75.5
F12	LAN5_not amplified	TUCR85	79.6	2	78.6, 81.6
C1	GI-ME-N_amplified	TUCR86	79.7	1	78.7
C2	GI-ME-N_amplified	TUCR86	79.7	1	78.5
A1	GI-ME-N_not amplified	TUCR86	79.7	1	79
A2	GI-ME-N_not amplified	TUCR86	79.7	1	79
D2	LAN5_amplified	TUCR86	79.7	0	
D1	LAN5_amplified	TUCR86	79.7	1	78.7
B1	LAN5_not amplified	TUCR86	79.7	0	
B2	LAN5_not amplified	TUCR86	79.7	0	
C3	GI-ME-N_amplified	TUCR87	75.5	1	76.2
C4	GI-ME-N_amplified	TUCR87	75.5	1	76.3
A3	GI-ME-N_not amplified	TUCR87	75.5	1	76.4
A4	GI-ME-N_not amplified	TUCR87	75.5	1	76.7
D4	LAN5_amplified	TUCR87	75.5	0	
D3	LAN5_amplified	TUCR87	75.5	1	75.7
B3	LAN5_not amplified	TUCR87	75.5	1	75.9
B4	LAN5_not amplified	TUCR87	75.5	1	75.8
C5	GI-ME-N_amplified	TUCR88	89	1	87
C6	GI-ME-N_amplified	TUCR88	89	1	87.1
A5	GI-ME-N_not amplified	TUCR88	89	1	87.3
A6	GI-ME-N_not amplified	TUCR88	89	1	87.2
D6	LAN5_amplified	TUCR88	89	0	
D5	LAN5_amplified	TUCR88	89	1	87
B5	LAN5_not amplified	TUCR88	89	2	77.8, 82.6
B6	LAN5_not amplified	TUCR88	89	2	82.5, 86.8
C7	GI-ME-N_amplified	TUCR89	78.9	1	78.8
C8	GI-ME-N_amplified	TUCR89	78.9	1	78.9
A7	GI-ME-N_not amplified	TUCR89	78.9	1	79.2
A8	GI-ME-N_not amplified	TUCR89	78.9	1	79.3
D7	LAN5_amplified	TUCR89	78.9	1	78.8
D8	LAN5_amplified	TUCR89	78.9	1	78.8
B7	LAN5_not amplified	TUCR89	78.9	1	79
B8	LAN5_not amplified	TUCR89	78.9	1	79.1
G7	GI-ME-N_amplified	TUCR9	78.5	1	77.1
G8	GI-ME-N_amplified	TUCR9	78.5	1	77.2
E7	GI-ME-N_not amplified	TUCR9	78.5	1	77.1
E8	GI-ME-N_not amplified	TUCR9	78.5	1	77.1
H7	LAN5_amplified	TUCR9	78.5	0	
H8	LAN5_amplified	TUCR9	78.5	0	
F7	LAN5_not amplified	TUCR9	78.5	1	77.1
F8	LAN5_not amplified	TUCR9	78.5	2	73.0, 77.3
C10	GI-ME-N_amplified	TUCR90	82.5	1	80.3
C9	GI-ME-N_amplified	TUCR90	82.5	1	80
A10	GI-ME-N_not amplified	TUCR90	82.5	1	80.8
A9	GI-ME-N_not amplified	TUCR90	82.5	1	80.6

D10	LAN5_amplified	TUCR90	82.5	1	80.2
D9	LAN5_amplified	TUCR90	82.5	1	80.1
B10	LAN5_not amplified	TUCR90	82.5	1	80.5
B9	LAN5_not amplified	TUCR90	82.5	1	80.4
C11	GI-ME-N_amplified	TUCR91	77.3	1	73.7
C12	GI-ME-N_amplified	TUCR91	77.3	1	73.7
A11	GI-ME-N_not amplified	TUCR91	77.3	2	72.7, 77.4
A12	GI-ME-N_not amplified	TUCR91	77.3	2	72.7, 77.6
D11	LAN5_amplified	TUCR91	77.3	1	73.3
D12	LAN5_amplified	TUCR91	77.3	1	73.5
B11	LAN5_not amplified	TUCR91	77.3	1	73.6
B12	LAN5_not amplified	TUCR91	77.3	1	73.7
G1	GI-ME-N_amplified	TUCR92	83.9	1	83.6
G2	GI-ME-N_amplified	TUCR92	83.9	1	83.4
E1	GI-ME-N_not amplified	TUCR92	83.9	1	83.3
E2	GI-ME-N_not amplified	TUCR92	83.9	1	83.1
F1	LAN5_amplified	TUCR92	83.9	1	83.5
F2	LAN5_amplified	TUCR92	83.9	1	83.2
H1	LAN5_not amplified	TUCR92	83.9	1	83.7
H2	LAN5_not amplified	TUCR92	83.9	1	83.6
G3	GI-ME-N_amplified	TUCR93	78.4	1	77.7
G4	GI-ME-N_amplified	TUCR93	78.4	1	80.2
E3	GI-ME-N_not amplified	TUCR93	78.4	0	
E4	GI-ME-N_not amplified	TUCR93	78.4	1	81.4
F3	LAN5_amplified	TUCR93	78.4	0	
F4	LAN5_amplified	TUCR93	78.4	0	
H4	LAN5_not amplified	TUCR93	78.4	0	
H3	LAN5_not amplified	TUCR93	78.4	1	78
G6	GI-ME-N_amplified	TUCR94	79.4	10	
G5	GI-ME-N_amplified	TUCR94	79.4	11	
E5	GI-ME-N_not amplified	TUCR94	79.4	1	62.7
E6	GI-ME-N_not amplified	TUCR94	79.4	3	
H5	LAN5_amplified	TUCR94	79.4	11	
H6	LAN5_amplified	TUCR94	79.4	12	
F6	LAN5_not amplified	TUCR94	79.4	0	
F5	LAN5_not amplified	TUCR94	79.4	1	61.1
G7	GI-ME-N_amplified	TUCR95	80.9	1	76.2
G8	GI-ME-N_amplified	TUCR95	80.9	1	76.4
E8	GI-ME-N_not amplified	TUCR95	80.9	1	78.7
E7	GI-ME-N_not amplified	TUCR95	80.9	3	
H7	LAN5_amplified	TUCR95	80.9	1	76.4
H8	LAN5_amplified	TUCR95	80.9	1	76.4
F8	LAN5_not amplified	TUCR95	80.9	1	78.7
F7	LAN5_not amplified	TUCR95	80.9	2	78.6, 81.7
G10	GI-ME-N_amplified	TUCR96	80.8	1	79.4
G9	GI-ME-N_amplified	TUCR96	80.8	1	79.5
E10	GI-ME-N_not amplified	TUCR96	80.8	1	79.4
E9	GI-ME-N_not amplified	TUCR96	80.8	1	79.5
H10	LAN5_amplified	TUCR96	80.8	1	79.5
H9	LAN5_amplified	TUCR96	80.8	1	79.7
F10	LAN5_not amplified	TUCR96	80.8	1	79.4
F9	LAN5_not amplified	TUCR96	80.8	1	79.5
G11	GI-ME-N_amplified	TUCR97	74.9	1	75.9
G12	GI-ME-N_amplified	TUCR97	74.9	1	76.2
E11	GI-ME-N_not amplified	TUCR97	74.9	1	75.7
E12	GI-ME-N_not amplified	TUCR97	74.9	1	76
H11	LAN5_amplified	TUCR97	74.9	1	75.7
H12	LAN5_amplified	TUCR97	74.9	1	75.9
F11	LAN5_not amplified	TUCR97	74.9	1	75.7
F12	LAN5_not amplified	TUCR97	74.9	1	75.8
G10	GI-ME-N_amplified	TUCR98	79.9	1	78.8
G9	GI-ME-N_amplified	TUCR98	79.9	1	78.7
E10	GI-ME-N_not amplified	TUCR98	79.9	1	77.1
E9	GI-ME-N_not amplified	TUCR98	79.9	2	74.9, 80.5
D2	LAN5_amplified	TUCR98	79.9	0	

D1	LAN5_amplified	TUCR98	79.9	1	75.3
B1	LAN5_not amplified	TUCR98	79.9	1	75.9
B2	LAN5_not amplified	TUCR98	79.9	1	78.8
D3	GI-ME-N_amplified	TUCR99	79.3	0	
D4	GI-ME-N_amplified	TUCR99	79.3	0	
C3	GI-ME-N_not amplified	TUCR99	79.3	0	
C4	GI-ME-N_not amplified	TUCR99	79.3	0	
B3	LAN5_amplified	TUCR99	79.3	0	
B4	LAN5_amplified	TUCR99	79.3	0	
A3	LAN5_not amplified	TUCR99	79.3	0	
A4	LAN5_not amplified	TUCR99	79.3	1	78.4
