# Supplementary material for: Transcribed-ultra conserved region expression profiling from low-input total RNA
Source: BMC Genomics. 2010 Mar 3;11:149. doi: 10.1186/1471-2164-11-149 (PMC2838852; doi:10.1186/1471-2164-11-149)
Supplement: Additional file 3 — MIQE checklist. Checklist according to the Minimum Information for Publication of Quantitative Real-Time PCR Experiments. [file 1471-2164-11-149-S3.PDF]

Item to check	Importance	
Experimental design		
Definition of experimental and control groups	E	EXPERIMENTAL SAMPLES: Pre-amplified RNAs from LAN-5 and GI-ME-N neuroblastoma cell lines.<br>CONTROL SAMPLES: Non-amplified RNAs from LAN-5 and GI-ME-N neuroblastoma cell lines
Number within each group	E	2
Assay carried out by core lab or investigator's lab?	D	Assays were carried out by investigator's lab
Acknowledgement of authors' contributions	D	
Sample		
Description	E	LAN-5 and GI-ME-N neuroblastoma cell lines provided by Interlab Cell Line Collection (ICLC), http://www.iclc.it)
Volume/mass of sample processed	D	9x10^6 million of cells
Microdissection or macrodissection	E	Not applicable: samples are cell cultures
Processing procedure	E	Cells were cultured at 37°C and 5% CO_2 in RPMI 1640 medium (Lonza, Basel, Switzerland), supplemented with 2mM Lglutamine, 1% non-essential amino acids, and 10% foetal bovine serum. Medium were removed from each plate. Trypsin/EDTA solution was added and incubated until cells release. Cell suspension was transferred to a clean tube and centrifuged at 1,000 x g for 2 minutes to pellet the cells.
If frozen - how and how quickly?	E	Not frozen
If fixed - with what, how quickly?	E	Not fixed
Sample storage conditions and duration (especially for FFPE samples)	E	After trypsinization, cells were placed on ice and immediately processed for RNA isolation
Nucleic acid extraction		
Procedure and/or instrumentation	E	Total RNA was extracted by using the PerfectPure™ RNA Cell Kit (5Prime, Hamburg, Germany), following manufacture's protocol. Homogenization of samples was performed by vortex method. Briefly, after adding Lysis solution to the cell pellet, the pellet was dislodged from the bottom of the tube by using a pipet tip. Sample was vortex vigorously to resuspend the pellet until there were no visible cell clumps in the lysate. Centrifugation steps were performed by using Centrifuge 5424 (Eppendorf AG, Hamburg, Germany).<br>PRINCIPLE: Total RNA is purified by first adding cultured cells to a detergent/salt solution (a chaotropic guanidium isothiocyanate solution) to lyse the cells and eliminate endogenous RNase activity. Lysis and homogenization disrupts the cell membranes releasing RNA into the lysing solution, and shears the genomic DNA, decreasing the viscosity of the lysate. Next, the lysates are applied to the purification column to bind the RNA and wash away proteins, DNA, and other contaminants. Residual DNA is removed by on-column DNase tratment, and the DNA fragments and DNase are removed by subsequent washing steps. Finally, the purified RNA is eluted with DEPC-treated water.
Name of kit and details of any modifications	E	PerfectPure™ RNA Cell Kit (5Prime, Hamburg, Germany). We exactly followed manufacture's protocol. The only modification was in the Lysis step: the incubation time was extended for 30 minutes.
Source of additional reagents used	D	2-mercaptoethanol 98% (Acros Organics, NJ, USA); Absolute ethanol (Carlo Erba Reactifs, France); RNase-free Dnase I (Ambion, TX, USA)
Details of DNase or RNase treatment	E	On column RNase-free DNase I treatment: after Wash 1, 50 µl of DNase solution (25 Units of DNase in 10X DNase I Buffer) were added to the Purification Column and incubated at room temperature for 20 minutes. Then 200 µl DNase Wash Solution (component of the PerfectPure kit) were added to the column. The column was centrifuged at 13,000 x g for 1 minute in a Centrifuge 5424 (Eppendorf AG, Hamburg, Germany). Additional 200 µl DNase Wash Solution were added and the colum was centrifuged at 13,000 x g for 2 minutes. The column was transfer to a new collection tube and we proceed with Wash 2 and elutions as specified in the protocol.
Contamination assessment (DNA or RNA)	E	No-reverse transcription controls was used to assess absence of DNA for both RNA targets. With this purpose, amplification of 18S rRNA was performed in RNAs isolated from LAN-5 and GI-ME-N cells. We used VIC-labeled TaqMan Gene Expression assay (Applied Biosystems, Foster City, CA) in a total volume of 10 µl, containing 10 ng of RNA, 2.5x RealMaster Mix Probe (5Prime, Hamburg, Germany) and 20x primer/probe mix. Reactions were setup in Real-time white tubes (Eppendorf, Hamburg, Germany) and they were run on the Mastercycler® epRealPlex4 S system (Eppendorf). Cycling conditions were as follows: 95°C for 2 minutes, 40 cycles at 95°C for 15 seconds and at 60°C for 1 minutes. The signal of the amplification plot was very late (Cq>40).
Nucleic acid quantification	E	RNA quantification was assessed by using microfluidic analysis (Agilent Technologies' Bioanalyzer).
Instrument and method	E	Total RNA and small RNAs were quantified by RNA 6000 Nano® and Small RNA® assays, respectively, on the 2100 Bioanalyzer (Agilent Technologies, Santa Clara, CA), following manufacture's protocols.
Purity (A260/A280)	D	
Yield	D	
RNA integrity method/instrument	E	2100 Bioanalyzer (Agilent Technologies, Santa Clara, CA)
RIN/RQI or Cq of 3' and 5' transcripts	E	LAN-5: RIN=9.2; GI-ME-N: RIN= 9.2
Electrophoresis traces	D	Not performed
Inhibition testing (Cq dilutions, spike or other)	E	Not performed by using dilutions of samples or universal inhibition assays. The amplification product for the reference gene assay obtained in each cDNA target has been considered sufficient to rule out the presence of inhibitors of reverse-transcription activity or PCR, also taking into account the high quality of starting RNAs.
Reverse transcription		
Complete reaction conditions	E	REVERSE TRANSCRIPTION OF TOTAL RNA. RNA was reverse transcribed using 20 pmoles of random hexamers (Eppendorf, Hamburg, Germany) and 200 U of SuperScript II enzyme (Invitrogen Life Technologies, Carlsbad, CA) in a total reaction volume of 20 µl. The mixture was incubated in a Mastercycler® epGradient S (Eppendorf) at 25°C for 10 minutes, 42°C for 60 minutes, and 85°C for 5 minutes to stop the reaction.<br><br>AMPLIFICATION AND REVERSE TRANSCRIPTION OF TOTAL RNA. RNA was amplified and reverse transcribed by the WT-Ovation™ RNA Amplification System kit (NuGEN Technologies, San Carlos, CA) following manufacture's protocol and using the Mastercycler® epGradient S (Eppendorf).
Amount of RNA and reaction volume	E	REVERSE TRANSCRIPTION OF TOTAL RNA. Amount of RNA: 1 µg; Reaction volume: 20 µl<br><br>AMPLIFICATION AND REVERSE TRANSCRIPTION OF TOTAL RNA. Amount of RNA: 50 ng; Reaction volume: 42 µl
Priming oligonucleotide (if using GSP) and concentration	E	
Reverse transcriptase and concentration	E	REVERSE TRANSCRIPTION OF TOTAL RNA: 200 U of SuperScript II enzyme (Invitrogen Life Technologies, Carlsbad, CA)<br><br>AMPLIFICATION AND REVERSE TRANSCRIPTION OF TOTAL RNA: WT-Ovation™ RNA Amplification System kit (NuGEN Technologies, San Carlos, CA) components
Temperature and time	E	REVERSE TRANSCRIPTION OF TOTAL RNA: 25°C for 10 minutes, 42°C for 60 minutes, and 85°C for 5 minutes.<br><br>AMPLIFICATION AND REVERSE TRANSCRIPTION OF TOTAL RNA. Primer annealing. 65°C for 5 minutes; First strand synthesis: 4°C for 1 minute, 25°C for 10 minutes, 42°C for 10 minutes, 70°C for 15 minutes; Second strand synthesis: 4°C for 1 minute, 25°C for 10 minutes, 50°C for 30 minutes, 70°C for 5 minutes; Post second strand enhancement: 4°C for 1 minute, 37°C for 15 minutes, 80°C for 20 minutes; SPIA™ amplification: 4°C for 1 minute, 47°C for 60 minutes, 95°C for 5 minutes.
Manufacturer of reagents and catalogue numbers	D	
Cqs with and without RT	D*	Samples have been validated as DNA free by performing a no–reverse transcription control when first extracting RNA.
Storage conditions of cDNA	D	-20°C

**qPCR target information**		
If multiplex, efficiency and LOD of each assay	E	Not applicable since we performed monoplex qPCRs
Sequence accession number	E	T-UCR assays (Catalogue no: 'T-UCR-all', Primer Design Ltd): see "UCR sequences" below. Data from: Bejerano G, Pheasant M, Makunin I, Stephen S, Kent WJ, Mattick JS, Haussler D. Ultraconserved Elements in the Human Genome. Science, 304(5675), pp. 1321-1325 (2004).<br>Eukaryotic 18S rRNA TaqMan® Gene Expression Assay (P/N 4319413E, Applied Biosystems): X03205.1
Location of amplicon	D	
Amplicon length	E	T-UCR assays (Catalogue no: 'T-UCR-all', Primer Design Ltd): not available due to intellectual property of Primer Design Ltd. Approximately 200 bases<br><br>Eukaryotic 18S rRNA TaqMan® Gene Expression Assay (P/N 4319413E, Applied Biosystems): 187 bases
In silico specificity screen (BLAST, etc)	E	Primers for specific detection of T-UCRs were supplied pre-optimized by PrimerDesign Ltd, UK. Each T-UCR assay was individually validated and shown to be 100% specific and close to 100% efficient.<br>Primers for specific detection of 18S rRNA were supplied pre-optimized by Applied Biosystems, Foster City, CA. All TaqMan® Gene Expression Assays have been designed through Applied Biosystems' validated bioinformatics pipeline.
Pseudogenes, retropseudogenes or other homologs?	D	
Sequence alignment	D	
Secondary structure analysis of amplicon	D	
Location of each primer by exon or intron (if applicable)	E	Not applicable for T-UCR assays<br>Eukaryotic 18S rRNA assay location: 609
What splice variants are targeted?	E	Not applicable
**qPCR oligonucleotides**		
Primer sequences	E	Not available due to intellectual property of Primer Design Ltd and Applied Biosystems
RTPrimerDB identification number	D	
Probe sequences	D**	Not applicable for T-UCR assays (SYBR® green detection chemistry)<br>18S rRNA: not available due to intellectual property of Applied Biosystems
Location and identity of any modifications	E	T-UCR assays: no modifications (SYBR® green detection chemistry)<br>18S rRNA: VIC™ dye-labeled TaqMan® MGB probe
Manufacturer of oligonucleotides	D	PrimerDesign Ltd, Hants, UK
Purification method	D	
**qPCR protocol**		
Complete reaction conditions	E	T-UCRs were quantified by Transcribed Ultra Conserved Regions real-time PCR assays (PrimerDesign Ltd, Hants, UK) using SYBR® green detection chemistry. qPCR reactions were carried out in a total volume of 10 µl, containing 2 µl of diluted cDNA, 2.5x RealMaster Mix SYBR ROX (5Prime, Hamburg, Germany) and 150 nM of the specific T-UCR primer mix. Amplification of Eukaryotic 18S rRNA, used as reference gene, was performed by using VIC-labeled TaqMan Gene Expression assay (Applied Biosystems, Foster City, CA) in a total volume of 10 µl, containing 2 µl of diluted cDNA, 2.5x RealMaster Mix Probe (5Prime) and 20x primer/probe mix. Reactions were setup in 96-white-well Twin.tec® real-time plates (Eppendorf) by means of EpMotion 5070 Liquid Handling Workstation (Eppendorf, Hamburg, Germany). All reactions were performed in duplicate on the Mastercycler® epRealPlex4 S system (Eppendorf). Cycling conditions were as follows: 95°C for 2 minutes, 40 cycles at 95°C for 15 seconds and at 60°C for 1 minutes, followed by a melting curve (ramping from 60°C to 95°C in 20 minutes) to ensure the presence of the specific amplicon.
Reaction volume and amount of cDNA/DNA	E	Reaction volume: 10 µl; Amount of cDNA: 10 ng
Primer, (probe), Mg++ and dNTP concentrations	E	T-UCR primer mix: 150 nM/ 10 µl reaction; 18S rRNA primers: 900 nM each in the 20x pre-formulated mix; 18S rRNA hydrolysis probe: 250 nM in the 20x pre-formulated mix; 12.5 mM Magnesium acetate; 1.0 mM dNTPs with dUTP in the 2.5x masterMix
Polymerase identity and concentration	E	HotMaster Taq DNA Polymerase (5Prime, Hamburg, Germany). Polymerase concentration: 0.1 U/µl in the 2.5x MasterMix
Buffer/kit identity and manufacturer	E	2.5x RealMaster Mix SYBR ROX for T-UCR assays / 2.5x RealMaster Mix Probe for 18S rRNA assay (5Prime, Hamburg, Germany).
Exact chemical constitution of the buffer	D	
Additives (SYBR Green I, DMSO, etc.)	E	T-UCR assays: SYBR Green I (5Prime, Hamburg, Germany)
Manufacturer of plates/tubes and catalog number	D	96-white-well Twin.tec® real-time plates (Eppendorf, Hamburg, Germany, catalog number: 951022055) with heat sealing films (Eppendorf, Hamburg, Germany, catalog number: 951023060)
Complete thermocycling parameters	E	95°C for 2 minutes, 40 cycles at 95°C for 15 seconds and at 60°C for 1 minutes
Reaction setup (manual/robotic)	D	Reactions were setup by means of EpMotion 5070 Liquid Handling Workstation (Eppendorf, Hamburg, Germany)
Manufacturer of qPCR instrument	E	Mastercycler® epRealPlex4 S system (Eppendorf, Hamburg, Germany).
**qPCR validation**		
Evidence of optimisation (from gradients)	D	
Specificity (gel, sequence, melt, or digest)	E	T-UCR assays: melting curve analysis (ramping from 60°C to 95°C in 20 minutes. Fluorescence data are measured continously).<br>Predicted and measured melting temperature values are provided as Supplementary data.
For SYBR Green I, Cq of the NCT	E	The signal of the amplification plot was very late (Cq>35) and therefore there was a very high Cq value difference between the negative control and all the cDNA sample results.
Standard curves with slope and y-intercept	E	Primers for specific detection of T-UCRs were supplied pre-optimized by PrimerDesign Ltd, UK. Each T-UCR assay was individually validated and shown to be 100% specific and close to 100% efficient.<br><br>Primers/probe for specific detection of Eukaryotic 18S rRNA were supplied pre-optimized by Applied Biosystems, Foster City, CA. TaqMan® Gene Expression Assays were used as the Gold Standard in the MicroArray Quality Control (MAQC) Project, which compared data from seven microarray platforms (Nature Biotechnology, September 2006). TaqMan® Assays have the highest specificity, highest sensitivity and the largest dynamic range of any gene expression technology.
PCR efficiency calculated from slope	E	
Confidence interval for PCR efficiency or standard error	D	
r2 of standard curve	E	
Linear dynamic range	E	
Cq variation at lower limit	E	
Confidence intervals throughout range	D	
Evidence for limit of detection	E	
If multiplex, efficiency and LOD of each assay	E	Not applicable since we performed monoplex qPCRs
**Data analysis**		
qPCR analysis program (source, version)	E	RealPlex software v. 2.0 (Eppendorf, Hamburg, Germany)
Cq method determination	E	The threshold is used to specify Cq values of samples. The Cq value is the cycle in which the fluorescence signal intersects with the threshold. The threshold is determined using the Noiseband method: the threshold is specified so that it is significantly (10 times the standard deviation) above the noise of the baseline. The baseline is automatically calculated for every sample individually.
Outlier identification and disposition	E	Runs were performed in duplicate as instrument and liquid handling variations were shown to be minimal (mean SD for non-amplified RNA: GI-ME-N= 0.28, LAN-5= 0.26; mean SD for pre-amplified RNA:GI-ME-N= 0.12, LAN-5= 0.12). Single runs were excluded when the melting curve analysis revealed unintended amplification products: melting curves with more than one peak, or one single peak but with a melt temperature different from the expected one (calculated by PrimerDesign's design software, accounting salt conditions of the mastermix).
Results of NTCs	E	In each plate we excluded false positives and unintended amplification products (e.g., primer dimers) by performing melting curve analysis after each T-UCR amplification.
Justification of number and choice of reference genes	E	18S rRNA has been used as reference gene. This choice was based on:<br>i) Establishment of the optimal reference genes by the geNorm™ Housekeeping Gene Selection Kit and software (v. 3.4) (PrimerDesign Ltd, UK) using PerfectProbe™ detection chemistry. Briefly, we analyzed 10 neuroblastoma cell lines (ACN, GI-CA-N, GI-ME-N, HTLA, IMR32, LAN-5, SH-SY-5Y, SK-N-BE(2), SK-N-BE(2)C, SK-N-SH) to determine which control genes are most stable (among GAPDH, EIF4A2, CYC1, YWHAZ, UBC, TOP1, SDHA, RPL13A, B2M, ATP5B, ACTB, 18S). The output results showed that the 18S is one of the 3 most stable genes for human neuroblastoma cell lines, together with TOP1 and ATP5B, with an average expression stability value M = 0.56.<br>Moreover, RT-qPCR tests performed in our laboratory confirmed that 18S rRNA level is invariant among a panel of neuroblastoma cell lines and neuroblastoma tumors. According to such selection, 18S has been successfully used as reference genes in previous papers (i.e. Longo L. et al. Int. J. Oncology, 33: 985-91, 2008).<br>ii) The invariability of the 18S rRNA assay among samples used in the present study is shown by the small difference of standard deviations for the Cq variance when the test is performed in LAN-5 and GI-ME-N RNA: 0.17 for non-amplified RNAs and 0.18 for pre-amplified RNAs.
Description of normalisation method	E	Data normalization has been carried out against 18S rRNA as endogenous unregolated reference gene. For each cDNA, the duplicate T-UCR Cq values were averaged, and the normalized Cq was calculated by subtracting the mean Cq value for 18S rRNA from each T-UCR mean Cq value.
Number and concordance of biological replicates	D	
Number and stage (RT or qPCR) of technical replicates	E	qPCR reactions were performed in duplicate
Repeatability (intra-assay variation)	E	For each sample, standard deviation (SD) for the Cq variance between replicates has been used to express intra-assay variation.<br>In LAN-5 the percentage of T-UCR runs with a SD ≤ 1 is 95% in unamplified RNA and 99.7% in amplified RNA.<br>In GI-ME-N the percentage of T-UCR runs with a SD ≤ 1 is 97% in unamplified RNA, and 99.7% in amplified RNA.
Reproducibility (inter-assay variation, %CV)	D	
Power analysis	D	
Statistical methods for result significance	E	Fisher's exact test; linear correlation analysis
Software (source, version)	E	RealPlex software v. 2.0 (Eppendorf, Hamburg, Germany); MedCalc® software v. 10.0.1.0 (Mariakerke, Belgium); Microsoft Office Excel 2003
Cq or raw data submission using RDML	D	Yes, in Supplementary data

UCR sequences	
**name**	**Build 34 (hg16) coords**
uc.1	chr1:10307243-10307449
uc.2	chr1:10442089-10442295
uc.3	chr1:10460711-10460935
uc.4	chr1:10467795-10468153
uc.5	chr1:10490897-10491110
uc.6	chr1:10504667-10504967
uc.7	chr1:10545679-10545934
uc.8	chr1:10561364-10561579
uc.9	chr1:10634956-10635157
uc.10	chr1:10675120-10675394
uc.12	chr1:35077889-35078089
uc.13	chr1:35786852-35787088
uc.14	chr1:37908298-37908510
uc.15	chr1:37974370-37974602
uc.16	chr1:38041493-38041703
uc.17	chr1:38215445-38215681
uc.18	chr1:44128955-44129192
uc.19	chr1:44403606-44403861
uc.20	chr1:44415666-44415934
uc.21	chr1:48482903-48483137
uc.22	chr1:50376149-50376456
uc.23	chr1:50405695-50405923
uc.24	chr1:50469063-50469398
uc.25	chr1:50535952-50536186
uc.26	chr1:62739563-62739774
uc.27	chr1:62739797-62740086
uc.28	chr1:70066629-70066983
uc.29	chr1:87244976-87245194
uc.30	chr1:87451593-87451835
uc.31	chr1:88395168-88395420
uc.33	chr1:96743538-96743849
uc.34	chr1:96751973-96752180
uc.35	chr1:97465205-97465409
uc.36	chr1:108587040-108587303
uc.37	chr1:114578780-114578981
uc.38	chr1:161127332-161127555
uc.39	chr1:161210912-161211267
uc.40	chr1:161825339-161825585
uc.41	chr1:210655265-210655480
uc.42	chr1:212945530-212945795
uc.43	chr1:240842759-240843015
uc.44	chr1:241164431-241164660
uc.45	chr1:241963410-241963612
uc.46	chr1:241964327-241964543
uc.47	chr2:7796390-7796616
uc.48	chr2:20462845-20463142
uc.49	chr2:33787944-33788150
uc.50	chr2:38950836-38951057
uc.51	chr2:57947093-57947299
uc.52	chr2:59082720-59082993
uc.53	chr2:59107845-59108076
uc.54	chr2:59173937-59174145
uc.55	chr2:59721112-59721351
uc.56	chr2:59922365-59922566

uc.57	chr2:60113774-60114019
uc.58	chr2:60272497-60272699
uc.59	chr2:60272917-60273136
uc.60	chr2:60416094-60416310
uc.61	chr2:60662107-60662432
uc.62	chr2:60755216-60755449
uc.63	chr2:61727035-61727312
uc.64	chr2:63168625-63168869
uc.65	chr2:66273125-66273336
uc.66	chr2:73149541-73149787
uc.67	chr2:104358126-104358342
uc.68	chr2:144125491-144125745
uc.69	chr2:144322879-144323179
uc.70	chr2:144648108-144648344
uc.71	chr2:144923625-144923872
uc.72	chr2:144925741-144926147
uc.73	chr2:144973082-144973282
uc.74	chr2:145036732-145037269
uc.75	chr2:145356619-145356854
uc.76	chr2:145371902-145372236
uc.77	chr2:145396534-145396829
uc.78	chr2:145399123-145399370
uc.79	chr2:145407946-145408240
uc.80	chr2:145411621-145411914
uc.81	chr2:147344834-147345044
uc.82	chr2:156929644-156929853
uc.83	chr2:157194172-157194467
uc.84	chr2:157397251-157397459
uc.85	chr2:157753959-157754206
uc.86	chr2:157862655-157862994
uc.87	chr2:158102814-158103103
uc.88	chr2:162297586-162297897
uc.89	chr2:162441217-162441523
uc.90	chr2:162475571-162475776
uc.91	chr2:163247566-163247772
uc.92	chr2:164653223-164653531
uc.93	chr2:164864451-164864713
uc.94	chr2:165046714-165046913
uc.95	chr2:171774074-171774324
uc.96	chr2:173023218-173023478
uc.97	chr2:173025175-173025616
uc.98	chr2:173159062-173159299
uc.99	chr2:173160925-173161322
uc.100	chr2:174317324-174317530
uc.101	chr2:174977025-174977278
uc.102	chr2:175148953-175149290
uc.103	chr2:175172216-175172448
uc.104	chr2:175189478-175189693
uc.105	chr2:175192331-175192553
uc.106	chr2:175227967-175228172
uc.107	chr2:175410152-175410388
uc.108	chr2:177142901-177143274
uc.109	chr2:177705882-177706105
uc.110	chr2:237358132-237358374
uc.111	chr3:9446461-9446756
uc.483	chr3:17567733-17568134

uc.112	chr3:18144568-18144913
uc.113	chr3:18651408-18651654
uc.114	chr3:18819454-18819747
uc.115	chr3:19009162-19009380
uc.116	chr3:70499494-70499699
uc.117	chr3:70792683-70792933
uc.118	chr3:70792935-70793153
uc.119	chr3:115754368-115754668
uc.120	chr3:115755940-115756209
uc.121	chr3:115896375-115896667
uc.122	chr3:115932792-115933006
uc.123	chr3:138304453-138304944
uc.124	chr3:138369353-138369639
uc.125	chr3:138389226-138389490
uc.126	chr3:138446557-138446827
uc.127	chr3:148351617-148351888
uc.128	chr3:148370547-148370845
uc.129	chr3:153485296-153485507
uc.130	chr3:159097487-159097710
uc.131	chr3:159310953-159311159
uc.132	chr3:159347072-159347279
uc.133	chr3:159347391-159347667
uc.134	chr3:159566817-159567027
uc.135	chr3:170155196-170155396
uc.136	chr3:170514865-170515211
uc.137	chr3:181757770-181758154
uc.138	chr3:186970209-186970627
uc.139	chr4:4587982-4588319
uc.140	chr4:12760753-12760975
uc.141	chr4:24280045-24280339
uc.142	chr4:41665611-41665869
uc.143	chr4:77037519-77037736
uc.144	chr4:83805060-83805264
uc.145	chr4:105805133-105805380
uc.146	chr4:112375296-112375509
uc.147	chr4:151814010-151814317
uc.148	chr4:152071579-152071818
uc.149	chr4:152071820-152072023
uc.150	chr5:3565359-3565620
uc.151	chr5:32425638-32425851
uc.152	chr5:50351523-50351723
uc.153	chr5:72279759-72279998
uc.154	chr5:72294088-72294290
uc.155	chr5:77018437-77018643
uc.156	chr5:77019492-77019704
uc.157	chr5:77025234-77025440
uc.158	chr5:77224326-77224549
uc.159	chr5:77232005-77232476
uc.160	chr5:77352917-77353238
uc.161	chr5:77442602-77442879
uc.162	chr5:81231434-81231651
uc.163	chr5:87252696-87253071
uc.164	chr5:87324478-87324680
uc.165	chr5:87777006-87777229
uc.166	chr5:88045878-88046187
uc.167	chr5:88263697-88263897

uc.168	chr5:91012866-91013085
uc.169	chr5:92995090-92995293
uc.170	chr5:93301743-93302052
uc.171	chr5:93649920-93650127
uc.172	chr5:93724792-93725009
uc.173	chr5:133802376-133802651
uc.174	chr5:138719870-138720129
uc.175	chr5:158322733-158322982
uc.176	chr5:167313590-167313835
uc.177	chr5:170398551-170398807
uc.178	chr5:170398920-170399168
uc.179	chr5:170609134-170609352
uc.180	chr5:170609411-170609635
uc.181	chr5:170610401-170610678
uc.182	chr5:170684001-170684239
uc.183	chr5:171365442-171365677
uc.184	chr5:173366215-173366444
uc.185	chr5:178157908-178158318
uc.186	chr5:179155889-179156193
uc.187	chr6:10502663-10502874
uc.188	chr6:16407363-16407577
uc.189	chr6:36614372-36614944
uc.190	chr6:41570295-41570494
uc.191	chr6:51123630-51123837
uc.192	chr6:51195833-51196076
uc.193	chr6:86317282-86317600
uc.194	chr6:93964662-93964862
uc.195	chr6:97708954-97709226
uc.196	chr6:98162138-98162358
uc.197	chr6:98408397-98408620
uc.198	chr6:98765487-98765793
uc.199	chr6:98859453-98859708
uc.200	chr6:99041131-99041384
uc.201	chr6:100097582-100097821
uc.202	chr6:101019581-101019810
uc.203	chr6:163900992-163901194
uc.204	chr7:1010242-1010443
uc.205	chr7:20574105-20574356
uc.206	chr7:20748081-20748579
uc.207	chr7:21555809-21556038
uc.208	chr7:23303942-23304159
uc.209	chr7:23304160-23304409
uc.210	chr7:26439350-26439606
uc.211	chr7:26471744-26472034
uc.212	chr7:26884210-26884414
uc.213	chr7:26925404-26925604
uc.214	chr7:31144670-31144912
uc.215	chr7:41933365-41933626
uc.216	chr7:50102955-50103266
uc.217	chr7:54378405-54378625
uc.218	chr7:69215092-69215377
uc.219	chr7:69392897-69393106
uc.220	chr7:96245647-96245903
uc.221	chr7:96253032-96253380
uc.222	chr7:113611674-113611874
uc.223	chr7:113612688-113612955

uc.224	chr7:113617522-113617816
uc.225	chr7:113627358-113627558
uc.226	chr7:113763821-113764025
uc.227	chr7:113849819-113850049
uc.228	chr7:114671200-114671464
uc.229	chr7:114689148-114689443
uc.230	chr7:114873964-114874201
uc.231	chr7:115136620-115136843
uc.232	chr7:121499109-121499355
uc.233	chr7:150220055-150220320
uc.234	chr7:156229240-156229511
uc.235	chr8:25797831-25798057
uc.236	chr8:37307753-37308019
uc.237	chr8:53187862-53188329
uc.238	chr8:53217077-53217434
uc.239	chr8:59992334-59992633
uc.240	chr8:65542500-65542705
uc.241	chr8:65547091-65547292
uc.242	chr8:66199258-66199522
uc.243	chr8:77740924-77741139
uc.244	chr8:105918932-105919252
uc.245	chr8:106290415-106290753
uc.246	chr8:119079806-119080089
uc.247	chr9:959154-959514
uc.248	chr9:964189-964410
uc.249	chr9:8085728-8085987
uc.250	chr9:13929910-13930118
uc.251	chr9:15864309-15864521
uc.252	chr9:16700753-16700983
uc.253	chr9:17322212-17322433
uc.254	chr9:23486725-23487003
uc.255	chr9:23681768-23681999
uc.256	chr9:23682234-23682439
uc.257	chr9:37205204-37205467
uc.258	chr9:37314424-37314624
uc.259	chr9:75084998-75085305
uc.260	chr9:76929543-76929773
uc.261	chr9:77328693-77329003
uc.262	chr9:79184841-79185095
uc.263	chr9:82047403-82047609
uc.264	chr9:82047611-82047877
uc.265	chr9:103498309-103498525
uc.266	chr9:104758130-104758372
uc.267	chr9:120429935-120430137
uc.268	chr9:120982873-120983123
uc.269	chr9:121913982-121914198
uc.270	chr9:123680114-123680391
uc.271	chr9:123680397-123680607
uc.272	chr9:123808633-123808845
uc.273	chr9:123893643-123893963
uc.274	chr9:123897916-123898242
uc.275	chr9:123960161-123960415
uc.276	chr9:123981857-123982288
uc.277	chr9:123983755-123984030
uc.278	chr9:124022210-124022446
uc.279	chr9:124048604-124048939

uc.280	chr9:124054051-124054270
uc.281	chr9:130771570-130771807
uc.282	chr9:135399783-135399989
uc.283	chr10:49949360-49949636
uc.284	chr10:49951459-49951667
uc.285	chr10:69860594-69860825
uc.286	chr10:76483627-76483878
uc.287	chr10:76840546-76840802
uc.288	chr10:77071786-77072008
uc.289	chr10:77335142-77335395
uc.290	chr10:77386885-77387090
uc.291	chr10:77628227-77628457
uc.292	chr10:98380042-98380258
uc.293	chr10:102037256-102037498
uc.294	chr10:102038204-102038647
uc.295	chr10:102039687-102039895
uc.296	chr10:102079693-102080153
uc.297	chr10:102083807-102084170
uc.298	chr10:102112245-102112603
uc.299	chr10:102174022-102174231
uc.300	chr10:102211705-102211912
uc.301	chr10:102232378-102232661
uc.302	chr10:102643767-102644107
uc.303	chr10:102717014-102717285
uc.304	chr10:102747091-102747362
uc.305	chr10:102876022-102876326
uc.306	chr10:102876626-102876849
uc.307	chr10:102908570-102908801
uc.308	chr10:102910399-102910675
uc.309	chr10:102931618-102931885
uc.310	chr10:114068810-114069038
uc.311	chr10:119738989-119739207
uc.312	chr10:119741124-119741445
uc.313	chr10:121004761-121004991
uc.314	chr10:124392535-124392736
uc.315	chr10:124392947-124393181
uc.316	chr10:126479965-126480204
uc.317	chr10:130920738-130920955
uc.318	chr10:131165986-131166306
uc.319	chr11:8269004-8269319
uc.320	chr11:8282143-8282477
uc.321	chr11:15588733-15588936
uc.322	chr11:16280660-16280882
uc.323	chr11:16439645-16439844
uc.324	chr11:30521830-30522054
uc.325	chr11:31649953-31650187
uc.326	chr11:31749989-31750303
uc.327	chr11:31750593-31750860
uc.328	chr11:31789972-31790202
uc.329	chr11:32162301-32162607
uc.330	chr11:66169256-66169462
uc.331	chr11:82921467-82921684
uc.332	chr11:115770342-115770678
uc.333	chr11:124182299-124182568
uc.334	chr11:131405597-131405818
uc.335	chr12:16606681-16606894

uc.336	chr12:24183273-24183523
uc.337	chr12:40035446-40035663
uc.338	chr12:52144756-52144978
uc.339	chr12:52357363-52357614
uc.340	chr12:52377099-52377357
uc.341	chr12:52669185-52669498
uc.342	chr12:52696761-52696987
uc.343	chr12:52708708-52709095
uc.344	chr12:52713153-52713406
uc.345	chr12:52733867-52734167
uc.346	chr12:105478977-105479178
uc.347	chr13:69591989-69592197
uc.348	chr13:69861358-69861597
uc.349	chr13:69919303-69919505
uc.350	chr13:70054101-70054340
uc.351	chr13:70466901-70467155
uc.352	chr13:70492166-70492365
uc.353	chr13:70569554-70569876
uc.354	chr13:76774830-76775064
uc.355	chr13:93316883-93317110
uc.356	chr13:95706821-95707071
uc.357	chr13:110664338-110664579
uc.358	chr14:24368162-24368387
uc.359	chr14:24905096-24905419
uc.360	chr14:24905510-24905796
uc.361	chr14:27223174-27223440
uc.362	chr14:27338791-27339029
uc.363	chr14:27851358-27851622
uc.364	chr14:28702798-28703004
uc.365	chr14:28732401-28732678
uc.366	chr14:29372746-29372947
uc.367	chr14:31834502-31834799
uc.368	chr14:32058615-32058842
uc.369	chr14:32112656-32112868
uc.370	chr14:32192610-32193002
uc.371	chr14:34010228-34010523
uc.372	chr14:34033074-34033350
uc.373	chr14:34571846-34572239
uc.374	chr14:35705952-35706175
uc.375	chr14:35767259-35767558
uc.376	chr14:43555788-43556077
uc.377	chr14:43569061-43569277
uc.378	chr14:78317518-78317768
uc.379	chr14:95421409-95421660
uc.380	chr14:95752635-95752866
uc.381	chr14:95869331-95869568
uc.382	chr15:33634968-33635167
uc.383	chr15:34535983-34536251
uc.384	chr15:34681449-34681714
uc.385	chr15:34901726-34901934
uc.386	chr15:35238065-35238267
uc.387	chr15:39748110-39748347
uc.388	chr15:55141581-55141878
uc.389	chr15:65381203-65381473
uc.390	chr15:65593988-65594192
uc.391	chr15:65756122-65756432

uc.392	chr15:68107952-68108207
uc.393	chr15:72630059-72630333
uc.394	chr15:94965868-94966069
uc.395	chr16:24545554-24545802
uc.396	chr16:48872692-48872899
uc.397	chr16:49513876-49514186
uc.398	chr16:49668919-49669240
uc.399	chr16:50627024-50627237
uc.400	chr16:51450088-51450293
uc.401	chr16:52273300-52273549
uc.402	chr16:53432987-53433231
uc.403	chr16:54102457-54102662
uc.404	chr16:55001959-55002193
uc.405	chr16:59350792-59351001
uc.406	chr16:69456552-69456762
uc.407	chr16:69457343-69457668
uc.408	chr16:72597321-72597572
uc.409	chr16:72869147-72869390
uc.410	chr17:35207780-35207998
uc.411	chr17:35525169-35525397
uc.412	chr17:35532036-35532303
uc.413	chr17:37941482-37941753
uc.414	chr17:38624137-38624382
uc.415	chr17:47138544-47138750
uc.416	chr17:47145525-47145810
uc.417	chr17:47156950-47157171
uc.418	chr17:56556866-56557082
uc.419	chr17:56557353-56557641
uc.420	chr17:63046872-63047104
uc.421	chr18:20945142-20945486
uc.422	chr18:21000175-21000400
uc.423	chr18:21008342-21008564
uc.424	chr18:21019766-21019980
uc.425	chr18:21117194-21117518
uc.426	chr18:22167579-22167840
uc.427	chr18:22489186-22489400
uc.428	chr18:28605196-28605434
uc.429	chr18:32732528-32732765
uc.430	chr18:33315637-33315849
uc.431	chr18:33430587-33430816
uc.432	chr18:33816919-33817129
uc.433	chr18:34315619-34315824
uc.434	chr18:43022775-43023023
uc.435	chr18:51238918-51239144
uc.436	chr18:51403228-51403437
uc.437	chr18:70484635-70484849
uc.438	chr18:70484851-70485091
uc.439	chr18:70490008-70490270
uc.440	chr18:70490342-70490670
uc.441	chr18:70695569-70695816
uc.442	chr18:70719689-70719937
uc.443	chr19:8433269-8433507
uc.444	chr19:35186619-35187006
uc.445	chr19:35258275-35258584
uc.446	chr19:35439694-35439965
uc.447	chr19:35459621-35459893

uc.448	chr19:35533370-35533601
uc.449	chr19:35695381-35695670
uc.450	chr19:36279466-36279676
uc.451	chr19:36498460-36498684
uc.452	chr19:36519787-36519990
uc.453	chr19:47129157-47129481
uc.454	chr20:4861440-4861647
uc.455	chr20:35043808-35044052
uc.456	chr20:42773185-42773504
uc.457	chr22:17770463-17770673
uc.458	chr22:34420305-34420508
uc.459	chrX:20895986-20896240
uc.460	chrX:24184937-24185211
uc.461	chrX:24226223-24226619
uc.462	chrX:24256252-24257030
uc.463	chrX:24277308-24277582
uc.464	chrX:24277584-24278353
uc.465	chrX:24278907-24279216
uc.466	chrX:24307884-24308232
uc.467	chrX:24369780-24370510
uc.468	chrX:24378989-24379477
uc.469	chrX:24379479-24379700
uc.470	chrX:24762642-24762982
uc.471	chrX:40239309-40239547
uc.472	chrX:40410244-40410445
uc.473	chrX:69240015-69240236
uc.474	chrX:69335634-69335843
uc.475	chrX:69632846-69633242
uc.476	chrX:80545473-80545710
uc.477	chrX:101813348-101813556
uc.478	chrX:121297212-121297463
uc.479	chrX:121311506-121311807
uc.480	chrX:121933027-121933228
uc.481	chrX:121933230-121933433
uc.483	chrX:137876095-137876389
