# Supplementary figures and images for: Transcribed-ultra conserved region expression profiling from low-input total RNA
Source: BMC Genomics. 2010 Mar 3;11:149. doi: 10.1186/1471-2164-11-149 (PMC2838852; doi:10.1186/1471-2164-11-149)

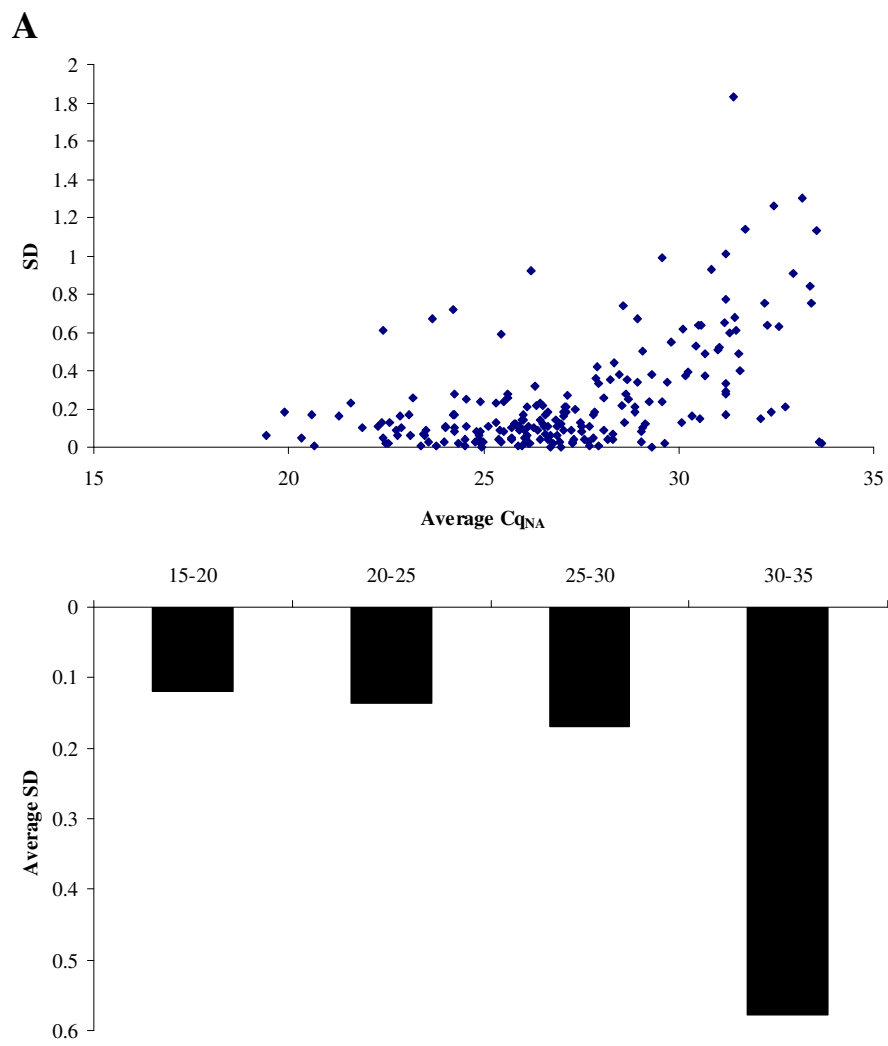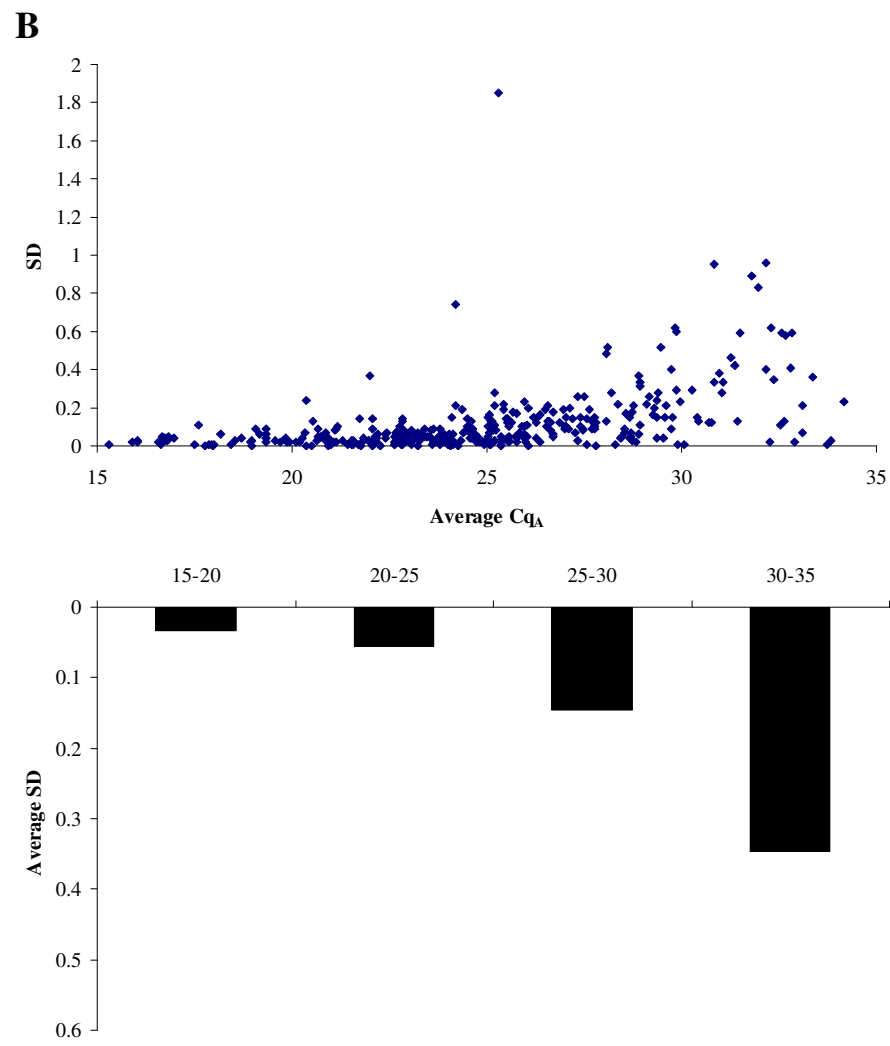

Supplement: Additional file 4 — Figure S1. Plots showing the correlation between the average Cq value and the standard deviation for T-UCRs in GI-ME-N cell line, using reverse transcriptase of total RNA (A) and amplification system (B). Bar plots display the mean SD value for T-UCRs with an average Cq value ranging between 15-20, >20-25, >25-30, and >30-35 cycles. [file 1471-2164-11-149-S4.PDF]
